# Abstract Proceedings of the 7th International Congress of the Molecular Biology Association of Turkey: İstanbul, Turkey. 27-29 September 2019.

**Published:** 2019-10-14

Abstract Proceedings of the 7th International Congress of the Molecular Biology Association of Turkey, İstanbul, Turkey. 27-29 September 2019.

