# Supplementary material for: Abstract Proceedings of the 7th International Congress of the Molecular Biology Association of Turkey: İstanbul, Turkey. 27-29 September 2019
Source: Turk J Biol. 2019 Oct 14;43(5 Suppl):1–132. (PMC6925586)
Supplement: Related texts

# Abstract: Regulation of sphingolipid metabolizing enzymes and BCR-ABL in human Ph+ acute lymphoblastic leukemia by resveratrol

**Authors:** Osman OGUZ, Aysun ADAN

**Affiliations:** Abdullah Gul University, Kayseri, Turkey

## Background/aim

Bioactive sphingolipids such as ceramide, sphingosine-1-phosphate (S1P) and glucosyl ceramide (GC), have significant roles in the regulation of cell division, growth, metastasis and apoptosis. Apoptotic ceramide produced through de novo synthesis pathway (Serine Palmitoyl Transferase (SPT), a key enzyme) is a central molecule in sphingolipid metabolism. The conversion of ceramide into S1P by sphingosine kinase 1 (SK-1) and into GC by glucosyl ceramide synthase (GCS) induces the proliferation of cancer cells. It is aimed to investigate therapeutic potential of resveratrol on Ph + ALL cells and to identify potential mechanisms behind resveratrol-mediated apoptosis in association with targeting of ceramide metabolism and regulation of BCR-ABL.

## Materials and methods

The antiproliferative, cytostatic and apoptotic effects of resveratrol, SPT inhibitor (myriocin), SK-1 inhibitor (SKI II), GCS inhibitor (PDMP) resveratrol:SKI II, resveratrol:PDMP and resveratrol: myriocin combinations on SUP-B15 ve SD-1 cells are investigated by cell proliferation assay, PI and annexin-V/PI staining by flow cytometry. Caspase-3 and PARP cleavage, cytochrome c release, BCR-ABL, SK-1, GCS and SPT expressions are checked by western blot.

## Results

Resveratrol and its combinations significantly induced apoptosis except combinations with myriocin and also caused cell cycle arrest at different phases and modulated the expression of BCR- ABL. Resveratrol modulated sphingolipid metabolism by altering the expression of key enzymes based on the cell type studied.

## Conclusion

Resveratrol modulated the expression of key sphingolipid enzymes involved in leukemia development and BCR-ABL. Combination of resveratrol with the inhibitors of sphingolipid enzymes could be a new approach for the treatment of Ph+ ALL.

## Keywords

Ph+ ALL,sphingosine kinase 1,apoptosis,combination therapy

## Acknowledgement

This project is supported by TUBITAK (project number: 315S248)

# Abstract: New long-form prolactin receptor isoforms: E10Δ50 and its variants

**Authors:** Mesut Cihan AYDEMIR, Mehmet Akif KILIC

**Affiliations:** Akdeniz University, Antalya, Turkey

## Background/aim

The prolactin receptor (PRLR) is a cytokine receptor found in vertebrates. It is associated with 300 functions. The reasons behind functional diversity are expressed in different periods of time, in different cells and in different forms. So far, 11 PRLRs have been identified in humans, 4 in mice and 2 in pigs and rats. There are data which indicates that there are undefined forms or 3'UTR isoforms in rats. Based on this information, a postgraduate thesis project aimed at recognizing new PRLR forms was carried out and following this, new forms of PRLR were determined through analysis of transcriptomic data sets.

## Materials and methods

In this study, over 100 transcriptomicdata sets were analyzed. Transcriptomic sequences were downloaded via NCBI's Sequence Read Archive. The CLC Genomics Workbench program was used to analyze downloaded sequences.

## Results

Two long form PRLR (LF) variants, which we call E10Δ50 forms, have been found. E10Δ50 forms are almost identical to the PRLR-LF, but correspond to a protein that differs from the last 15 amino acids of the PRLR-LF. In E10Δ50 forms, 50 nucleotides are introduced before the stop codon of the long form specific exon. Subsequently, the E10Δ50 forms are generated by exon splayzing either in the 96 nucleotide downstream area or specific to the short form. With the 50 nucleotides removed from E10Δ50 forms, the Y580 residue, which is functional in PRLR-LF, is lost.

## Conclusion

In previous function studies, it was found that replacing the Y580 at the PRLR-LF with a point mutation reduced the efficacy of the JAK/STAT pathway by 30%. Therefore, the main function of E10Δ50 forms is to reduce the effectiveness of the JAK/STAT pathway initiated by the PRLR-LF. It is even possible that forms E10Δ50 can activate different signaling pathways.

## Keywords

prolactin receptor,molecular biology,transcriptomics

# Abstract: Characterization of plasmodium and neospora OTU-like proteins and development of novel inhibitors OTU inhibitors

**Authors:** Sezer AKGOL, Pinar SIYAH, Fatih KOCABAS

**Affiliations:** Yeditepe University, Istanbul, Turkey

## Background/aim

OTU deubiquitinases (DUB) are key proteins to antagonize cellular defense pathways post entry of intracellular obligatory parasites into the host cell. Intriguingly, parasitic Plasmodium species (*P. falciparum, P.vivax, P.yoelii*) and *neospora caninum* pose previously uncharacterized proteins similar to viral OTU proteins due to similarity of conserved amino acid residues in their catalytic and inhibition pockets with viral OTU protein. However, structure, inhibitors or function of Plasmodium and Neospora OTU-like proteins have not been previously determined.

## Materials and methods

We have performed homology modeling, orphan drug analysis, recombinant protein expression and purification, DUB assays to determine OTU activity, potent inhibitors and their IC50 values. In addition, western blot and gene expression analysis conducted to determine intracellular deubiquitinase activity and characterize potent inhibitors of OTU-like proteins.

## Results

We have shown for the first time DUB activity of viral OTU-like proteins by fluorescent UB- AMC deconjugation assay. We determined potent inhibitors of malarial OTU proteins by screening of pathogen box compounds, viral OTU inhibitors, ZINC database, and a library of anti-malarial small molecules. MMV676605, MMV031011, and MMV011903 inhibited malarial OTU DUB activity in vitro. We developed novel OTU inhibitors that inhibit malarial (IC50 as 0.1 μM) and neospora (IC50 as 0.2 μM) OTU. Upon the small molecules treatments, we have observed regression in mono- and polyubiquitination cleavage levels. In addition, these molecules enabled gene expression levels to return levels of control by the increasing CASP1, NF-κB, IFNA1 and MX1 expression.

## Conclusion

These findings suggest that targeting plasmodium and neospora OTU proteins is a plausible strategy to develop new anti-malarial and anti-neosporosis therapies.

## Keywords

Malaria,plasmodium,neospora,OTU-like DUBs

## Acknowledgement

We like to thank to the support by The Scientific and Technological Research Council of Turkey (TUBITAK) ARDEB 1001 [# 215Z069] program and Bill & Melinda Gates foundation MMV Pathogenbox Award.

# Abstract: Development of fluorescence imaging tools for investigation of myelin mechanics

**Authors:** Zeynep ALADAG, Bilal E. KERMAN

**Affiliations:** Istanbul Medipol University, Istanbul, Turkey

## Background/aim

Oligodendrocytes found in the central nervous system are responsible for the formation of myelin sheath, which provides trophic support to neurons and facilitates transmission of their electrical signals. Therefore, myelin sheath plays an essential role in the function of the nervous system. When the myelin sheath is lost or damaged, electrical signal transmission is disrupted, and neurons become atrophied causing several diseases such as multiple sclerosis (MS), Pelizaeus- Merzbacher disease (PMD), and adrenoleukodystrophy. Myelination mechanism is well studied by immunohistochemistry and microscopical methods. However, because myelin sheath is a dynamic structure, live imaging is key in understanding the mechanism of myelin formation. Our main aim is to understand the detailed mechanism of myelin and neural membrane interactions by visualizing myelination using fluorescent proteins.

## Materials and methods

Neuronal membrane protein Caspr and its partner on the oligodendrocyte membrane, NF155, bind to each other at early stages of and stay bound together during myelin formation. To visualize membrane-membrane interactions during myelination, we cloned fluorescent tagged versions of NF155 and Caspr proteins under oligodendrocyte and neuron specific promoters, myelin basic protein (MBP) and synapsin (Syn), respectively. The lentiviral constructs of MBP:NF155- mCherry and Syn:Caspr-GFP were transfected in HEK293T cells to test their expression.

## Results

Our preliminary results showed that MBP:NF155-mCherry and Syn:Caspr-GFP constructs were packaged into lentiviral particles and were transduced mammalian cells. More, NF155-mCherry and Caspr-GFP proteins were expressed by mammalian cells.

## Conclusion

Understanding the mechanism and dynamics of myelination will improve our basic knowledge of the nervous system and will also elucidate mechanisms of myelin diseases such as MS. MBP:NF155-mCherry and Syn:Caspr-GFP are promising tools to investigate interaction between oligodendrocyte and neuron membranes.

## Keywords

Myelin,Oligodendrocyte,Multiple Sclerosis,Live Imaging

## Acknowledgement

This project is supported by The Scientific and Technological Research Council of Turkey (TUBITAK) with the project number: 316S026.

# Abstract: The effect of curcumin on apoptosis in human glioma cells

**Authors:** Seniz ASLAN, Ozge ANAC, Evren ONAY UCAR

**Affiliations:** Istanbul University, Istanbul, Turkey

## Background/aim

Glioma is the most malign and aggressive primary brain tumor. New therapies are urgently needed against standard glioma therapy because of the drug resistance of glioma cells. In this case, the results obtained from plant-based approaches are very important. Curcumin which is found in the rhizome of turmeric (*Curcuma longa*) and gives yellow-orange color to turmeric, is an example of these approaches. Antioxidant, anti-angiogenic, antimutagenic features of curcumin were found in the chemical structure of polyphenol. These features make a good agent for cancer studies.

## Materials and methods

Cytotoxicity test was performed with MTT to determine the toxic effect of curcumin on U87-MG human glioma cells. The effects of curcumin (2.5 and 5 μM) were evaluated on the expressions of caspase-3 and Bax protein by Western Blot analysis. Differences among groups were tested by one-way ANOVA followed by Tukey post-hoc-test.

## Results

As a result of cytotoxicity analysis, it was observed that curcumin was effective even at low doses. Western Blot analysis showed that curcumin caused upregulation of caspase-3 expression and downregulation of Bax expression in glioma cells.

## Conclusion

Considering all studies, it has been concluded that curcumin could be a good agent for glioma therapy. Future studies are needed to clarify how the other pro-apoptotic and anti-apoptotic proteins will be effected under the different concentrations of curcumin in glioma cells.

## Keywords

Curcumin,glioma,apoptosis

## Acknowledgement

This research supported by Istanbul University Scientific Research Project Unit with project number FLO-2019-32922.

# Abstract: Class IA PI3K isoforms lead to differential dignaling downstream of PKB/AKT

**Authors:** Hazal Beril ATALAK, Onur CIZMECIOGLU

**Affiliations:** Ihsan Dogramaci Bilkent University, Ankara, Turkey

## Background/aim

PI3K pathway has been deregulated in one third of human cancers. All ClassIA PI3Ks, which are composed of catalytic and regulatory subunits, catalyze conversion of PIP2 to PIP3 on plasma membrane. The catalytic subunits of ClassIA - p110α, p110β, and p110δ -, are found to be mutated/amplified in cancers. As inhibiting all ClassIA catalytic isoforms lead to widespread toxicity, identification of isoform specific targets of especially p110α and p110β have the potential to transform targeted therapy for PI3K deregulated cancers.

## Materials and methods

Isogenic mouse embryonic fibroblasts (MEFs) were used as they constitute a genuine model for signaling experiments with their genetically stable, and non-transformed phenotype. MEFs were engineered to have their first exons of p110α and β floxed, enabling their knock out in a temporally regulated manner, which allowed us to study their isoform specific targets. Myristoylation (Myr), a lipidation signal anchors proteins to the plasma membrane, leads to constitutive activation of kinases. We tagged p110s with Myr signal and transfected MEFs with them to study their targets. Proliferation assays, pharmacological inhibition, Western Blots are used to elucidate the unique targets of p110 isoforms.

## Results

Myristoylated p110s result in activation of unique as well as common Akt substrates. These unique targets were highlighted in proliferation experiments where MEFs were treated either with Doxorubicin, a chemotherapeutic that is a topoisomerase II inhibitor, to induce apoptosis, or MK2206, a pan - Akt inhibitor. mTORC1 inhibition by Rapamycin, mTORC1 and mTORC2 inhibition by Everolimus, and Rac1 inhibition by EHT1864 translate differentially to the corresponding downstream targets in p110s.

## Conclusion

This study suggests differential regulation of translation, metabolism as well as survival signalling downstream of unique p110 isoforms.

## Keywords

Phosphoinositide 3-kinase,Protein Kinase B,Mammalian Target of Rapamycin,Ras- related Botulinum Toxin Substrate 1

# Abstract

**Authors:** Salih KUMRU, Safak KALINDAMAR, Jochen BLOM, Selcuk HAZIR

**Affiliations:** 1 Recep Tayyip Erdogan University, Rize, Turkey; 2 Ordu University, Ordu, Turkey; 3 Justus-Liebig-University Giessen, Giessen, Germany; 4 Aydin Adnan Menderes University, Aydin, Turkey

## Background/aim

The *Photorhabdus* genus consists of bioluminescent Gram-negative bacteria, which are members of the *Enterobacteriaceae* family. *Photorhabdus* are lethal insect pathogens that are mutualistic partner of entomopathogenic nematodes in the genus *Heterorhabditis*. One species of *Photorhabdus* (*P. asymbiotica*) is associated with human skin infection. *Photorhabdus* species secrete toxins and exoenzymes that target the cellular branch of innate immunity of insects. However, identification of these secretion systems in *Photorhabdus* have been missing. Thus, we conducted a comparative analysis of *Photorhabdus* genomes to determine secretion systems, and a phylogeny analysis of *Photorhabdus* genomes to understand phylogenetic relationship among the *Photorhabdus* strains.

## Materials and methods

The genome sequences of 45 *Photorhabdus* strains were downloaded from NCBI (National Center for Biotechnology Information). By EDGAR, a phylogenetic tree was built from the core genomes of 45 *Photorhabdus* strains. Average Nucleotide Identity (ANI) value was calculated, and Pan-Core genome development plot was created. To identify the secretion systems of *Photorhabdus* genomes, MacSyFinder was used.

## Results

*Photorhabdus* asymbiotica strain ATCC43949, bioluminescent clinical isolate, was the only plasmid-carrying genome from evaluated *Photorhabdus* genomes. The phylogenetic tree was built from the 1025 core gene sets of 45 genomes, and it was supported by ANI value. Our results indicated that all genomes had Type I (T1SS), Type III (T3SS), Type V (T5SS), Type VI (T6SS) secretion systems and flagellum genes. All genomes had partial of Type IV pilus genes. There were variations in the number of T1SS and T6SS genes among the *Photorhabdus* genomes.

## Conclusion

We expected to improve the phylogeny of *Photorhabdus* and reclassification of strains. This is a recent report of *Photorhabdus* secretion systems and comparative genomics approaches to understand the secretion systems and phylogeny.

## Keywords

*Photorhabdus*,entomopathogens,comparative genomics,bacterial secretion systems

# Abstract: Investigation of thymoquinone usability on wound healing

**Authors:** Nihan Sultan MILAT, Aslihan SENGELEN, Murat PEKMEZ

**Affiliations:** Istanbul University, Istanbul, Turkey

## Background/aim

Thymoquinone (TQ), a well-known active constituent of *Nigella sativa*, has been extensively investigated for its pharmacological activity such as anti-inflammatory, antioxidant and antimicrobial effects. These properties make *Nigella sativa* and TQ an important potential agent for wound healing activity. Although there have been numerous studies on wound healing effect of *Nigella sativa*, studies on thymoquinone are still insufficient. In this study, we aimed to investigate the in vitro wound healing potential of TQ.

## Materials and methods

We used NIH/3T3 mouse embryonic fibroblast cells to evaluate the wound healing effect of TQ. MTT assay was used to measure its cytotoxic and proliferative effects. 0.1, 1 and 10 μM TQ were applied to the cells. Its effect on wound healing after 18- and 24- hours recovery was examined by in vitro scratch assay. Also, the level of β-catenin, an effective protein in wound healing processes was determined by Western blot assay. The statistical evaluation was performed with two- way ANOVA followed by Tukey post-hoc-test. The criterion for statistical significance was P < 0.05.

## Results

MTT analysis revealed that low doses of TQ had a proliferative effect on the cells. In vitro scratch assay data showed that treatment with 1 and 10 μM TQ resulted in a statistically significant recovery (91.35% and 90.84%, respectively) in wound area compared to control. Furthermore, we observed statistically significant increase of β-catenin protein level which is supported our data.

## Conclusion

All these results highlight that thymoquinone has an important value to investigate its usability on wound healing as an active substance.

## Keywords

Thymoquinone (TQ),NIH/3T3,wound healing

## Acknowledgement

This study was supported by TÜBITAK (Scientific and Technological Research Council of Turkey) 2209-B (Project No:1139B411801008) and Istanbul University BAP (Project No: FLO-2018-32851).

# Abstract: Anti-inflammatory Effects of UA and OPC Isolated from *Capparis ovata*

**Authors:** Elif KALE, Ozden OZGUN ACAR, Hajarat ABILO ALFA, Isil GAZIOGLU, Gulacti TOPÇU, Alaattin SEN

**Affiliations:** 1 Pamukkale University, Denizli, Turkey; 2 Bezmialem Vakif University, Istanbul, Turkey; 3 Abdullah Gül University, Kayseri, Turkey

## Background/aim

Macrophage-derived inducible nitric oxide synthase enzyme (iNOS) is respon- sible for excess NO production in damaged tissues during inflammation process. Inhibition of iNOS enzyme activity plays an essential role in the treatment of many diseases. This study aims to investi- gate the effect of ursolic acid (UA), olean-12-en-28-ol, 3β pentacosanoate (OPC) and rutin isolated from *Capparis ovata* on the NO synthesis by the iNOS.

## Materials and methods

RAW 264.7 cells were cultured in RPMI-1640 medium. Cells were treated with varying concentrations of compounds and LPS for 24 h at 37 C. During this process, cell viability was assessed using crystal violet staining. The most effective dose of LPS for stimulation of the cells and non-toxic doses for compounds were determined. After stimulation with LPS, the determined doses of compounds were applied. The nitrite levels in LPS-induced macrophage cells were measured according to the Griess method in extracellular supernatants to investigate the effect of compounds on NO synthesis by the iNOS

## Results

A significant reduction was measured in the amount of nitrite detected in extracellular supernatants following the application of compound compared to the amount of nitrite measured only in LPS-induced cells.

## Conclusion

The data suggested that these compounds exhibit anti-inflammatory properties, via inhibiting the synthesis of NO by iNOS in LPS-induced cells. Also, the determined doses of these compounds have no adverse effecton cell survival during incubation as an anti-inflammatory agent.

## Keywords

*Capparis ovata*,Inflammation,Nitric oxide,iNOS

## Acknowledgement

This work supported by Pamukkale University PAUBAP-2017FEBE060

# Abstract: Cladribine induced apoptosis in glioblastoma cells

**Authors:** Gurbet ÇELIK TURGUT, Alaattin SEN

**Affiliations:** 1 Pamukkale University, Denizli, Turkey; 2 Abdullah Gül University, Kayseri, Turkey

## Background/aim

Cladribine is antineoplastic agents that act as an inhibitor of DNA synthesis and repairs and induce cell death. It is used in the treatment of lymphatic diseases and multiple sclerosis. There have been many studies showed the effect of cladribine on lymphatic system cells, but no reports on the apoptotic effects of cladribine in human glioblastoma cell line have been investigated. Our study aimed to investigate whether cladribine has an apoptotic effect on glioblastoma cells.

## Materials and methods

For this purpose, we first determined the cytotoxicity of cladribine in U-118 MG cells by crystal violet staining assay. The expression analysis of apoptotic genes was detected by qRT-PCR after cladribine treatment at non-toxic dose. Then, we have followed the induction of apoptosis by cladribine in U-118 MG cells applying DNA fragmentation assay.

## Results

It was found that 0.035 mM cladribine was the approximately 10% non-toxic dose for U-118 MG cells. mRNA levels of BCL-2, MAPK, RAF and JAK were decreased 80%, 66%, 73% and 69% by the cladribine treatment, respectively. On the other hand, P53, BAX, FASLG, ATM, ATR, ATRIP, BRCA1, H2AFX and PARP1 mRNA level increased 85%, 69%, 65%, 90%, 78%, 66%, 60%, 77% and 57% as a result of the cladribine treatment. Unlike these mRNA levels, P73, JUN and MYC mRNAs were not altered by the cladribine. Moreover, the induction of DNA fragmentation assay indicates that cladribine induces the apoptosis in U 118 MG cells.

## Conclusion

These results suggested that cladribine induces apoptosis by through the intrinsic pathway and DNA strand breaks in the glioblastoma cells.

## Keywords

cladribine,apoptosis,glioblastoma cells,DNA fragmentation

# Abstract: Rare skeletal dysplasia: two sisters diagnosed with Pycnodysostosis

**Authors:** Ahmet ARMAN, Ceren ALAVANDA, Hamza POLAT, Bilgen Bilge GECKINLI, Esra ARSLAN ATES, Kadir Sinan ARSLAN, Mehmet Ali SOYLEMEZ

**Affiliations:** Marmara University, Istanbul, Turkey

## Background/aim

Pycnodysostosis isanautosomal recessive inherited skeletal dysplasiacharacterized by increased bone density, short stature, open and wide anterior fontanelles and acroosteolysis. Mutations in the *CTSK* gene are responsible for the phenotype of disease.

## Materials and methods

Patient: A 5-year-old female patient was consulted to our clinic for open fontanelles and short stature. Developmental stages were normal and short hand fingers were detected. The patient had no history of fracture. The bone radiographs showed increased bone density and the patient's bone age was one year behind the calendar age. Although the patient had a similar phenotype in her 1-year-old sister, there was no similar history in the family. After DNA isolation from peripheral blood, 8 exons and exon-intron boundaries of *CTSK* gene from the patient, her parents and sister were amplified and sequenced by Sanger method.

## Results

Karyotype of the patient is 46, XX. Sequence analysis showed that homozygote c.721C> T (p.R241X) mutation was detected on *CTSK* gene. This mutation is pathogenic based on silico analysis and HGMD database. Segregation analysis showed that her mother and father were heterozygote but her sister is homozygote for this mutation.

## Conclusion

Nonsense mutation was detected in Pycnodysostosis patient. Genetic analysis is important and necessary for screening individuals at risk in the family and providing appropriate genetic counseling.

## Keywords

Molecular biology,genome,biomedicine

## Acknowledgement

This project was supported by GEN ERA Diagnostic.

# Abstract: Generation of induced pluripotent stem cell (iPSC)-based models of Friedreich's Ataxia

**Authors:** Simge KELEKÇI, Esra BÖRKLİ YÜCEL, Tamer ÖNDER

**Affiliations:** Koç University, Istanbul, Turkey

## Background/aim

Induced pluripotent stem cells (iPSCs), which are reprogrammed from differentiated cells back to an embryonic-like state, can be used to generate disease specific cell types to study neurodegenerative diseases. Friedreich's ataxia (FRDA) is a hereditary neurodegenerative disease characterized by progressive ataxia of limbs, dysarthria, sensory neuropathy and cardiomyopathy. FRDA results in reduced Frataxin protein levels. However, link between reduced Frataxin level and disease-associated pathologies remains unclear. In this study, our aim is to generate an in vitro model of FRDA and thus identify pathways, genes and/or proteins involved in FRDA and validate the effects of candidate drugs on in vitro FRDA models.

## Materials and methods

Fibroblasts collected from 3 FRDA patients and 2 healthy individuals were reprogrammed to iPSCs by episomal vector nucleofection. Integration of episomal vectors was assessed with a PCR-based detection method. Expression of pluripotency genes in iPSCs was tested by real time PCR analysis of *OCT4, SOX2, NANOG, LIN28A* and by immunofluorescence staining using antibodies against OCT4, NANOG, SSEA4. Ability of iPSCs to differentiate into all three germ layers was assessed by formation of embryoid bodies.

## Results

We derived iPSCs from FRDA patients and healthy individuals with goal of differentiating them into neurons and cardiomyocytes. The resulting iPSCs were characterized with respect to expressions of pluripotency markers, and ability to differentiate into all three germ layers with normal karyotypes along with lack of genomic integration of episomal vectors.

## Conclusion

After characterization, iPSCs will be converted to cardiomyocytes and neurons. Disease- specific mechanisms such as iron accumulation and mitochondrial dysfunction upon oxidative stress will be examined in these cardiomyocytes and neurons. Finally, to reverse any potential abnormalities in the in vitro FRDA models, we will test therapeutic strategies to examine the effects of candidate drugs on the disease phenotypes.

## Keywords

Induced pluripotent stem cells,Friedreich's ataxia,characterization

# Abstract: Investigation of cytotoxic activity of cerulenin and vitamin E combination in U87 glioma cell line

**Authors:** Naz UZUNALIOGLU, Elif MERTOGLU, Murat PEKMEZ

**Affiliations:** Istanbul University, Istanbul, Turkey

## Background/aim

Vitamins have been suggested to be an effective antioxidant to protect against carcinogenesis, and metabolic differences between tumors and normal cells are used as a potential treatment in cancer. In this study, a combination of Cerulenin and Vitamin E (α-TOS) was applied to the U87 glioma cell line to determine the concentration that causes 50% cell death.

## Materials and methods

The viability of the test materials and controls were determined using the MTT Test. After determining the cytotoxic doses (CD_50_) of test materials separately, the concentration of Cerulenin was kept constant and experimental groups were generated as in four different pairs.

## Results

CD_50_ causing 50% death of U87 glioma cells were found 5.2 μg/ml for Cerulenin and 47 μM for α-TOS. It was observed that the dual mixture containing the CD_50_ dose of both test materials killed 70% of the cells and the viability gradually increased as the α-TOS concentration was reduced in the rest of trials. Among the other combinations in the experimental groups, the dose affecting the cell proliferation closest to 50% was determined as 33 μM α-TOS + 5.2 μg/ml Cerulenin

## Conclusion

Glioblastoma multiform (GBM) is still the most difficult cancer to treat in clinical oncology. α-TOS has been found to induce apoptosis specifically in tumor cells without damaging normal cells. Overexpression of Fatty acid synthase (FAS) has been observed in many types of cancer compared to normal tissues. However, it is thought that α-TOS suppresses the mechanism of action of Cerulenin in these dual combinations. Since no similar studies have been conducted on glioma in previous years, these findings have been presented for the first time with this study.

## Keywords

U87 cell line,Cerulenin,α-TOS,MTT Assay

## Acknowledgement

This research is supported by Istanbul University Scientific Research Projects Unit (BAP) project number 32826.

# Abstract: Determination of therapeutic effects of nilotinib on breast cancer cells

**Authors:** Polen YUNUS, Gizem Tugce ULU, Muharrem Sevki PAZARCEVIREN, Yagmur KIRAZ, Yusuf BARAN

**Affiliations:** Izmir Institute of Technology, Izmir, Turkey

## Background/aim

Breast cancer is subcategorized into three as follows: estrogen, progesterone and human epidermal growth factor receptor 2 (Her2) positive. Her2 is known by its regulatory effects on cell proliferation, growth, invagination, and migration. Increased ABL1 and ABL2 activation were observed in invasive breast cancer types. Also, ABL kinase is a downstream target of Her2. It was suggested that targeting PDGF/Abl signaling pathway could be applied against resistance to aromatic inhibition. Nilotinib is a tyrosine kinase inhibitor which inhibits BCR-ABL1, ABL1, ABL2 and PDGFR activities. The aim of the study is targeting Her2+ breast cancer cells by using an Abl inhibitor, nilotinib to test its potential cytotoxic effects and exploring the mechanism.

## Materials and methods

Antiproliferative effects of nilotinib on MDA-MB-231 (Her2 negative) and SKBR-3 (Her2+) cells was determined by using MTT Cell Proliferation Assay and Trypan Blue Staining. Annexin V/PI Double Staining by using fluorescence microscopy was applied for detecting apoptosis and necrosis. Finally, wound healing assay was performed to see the effect of nilotinib on cell migration.

## Results

MTT and Trypan Blue Staining Assay results indicated that nilotinib showed cytotoxic effects on Her2+ SKBR-3 cells more than Her2- cells. IC50 value of nilotinib was detected as 13.5 μM and more than 20 μM for SKBR-3 and MDA-MB-231 cells, respectively. Nilotinib at its IC50 concentration, induced more apoptosis in SKBR-3 cells and more necrosis in MDA-MB-231 cells. However, increasing nilotinib concentration induced necrosis on SKBR-3 cells. Besides, there was no specific effect of nilotinib on cell migration.

## Conclusion

This study shows that Abl inhibitor, nilotinib is more effective on Her2+ cells than Her2- cells. Further experiments will be performed to determine cytostatic and genotoxic effects of nilotinib on breast cancer cells. Also, molecular regulation between Her2 and Abl will be determined.

## Keywords

Breast cancer,Her2,Nilotinib

# Abstract: Heterodimerization, secretion and expression patterns of DEK isoforms 1 and 2

**Authors:** Emrah ÖZÇELIK, Büsra CELIK, Ahmet KALAYCI, Ayten KANDILCI

**Affiliations:** Gebze Technical University, Gebze, Kocaeli, Turkey

## Background/aim

DEK has two known isoforms (DEK1 and DEK2), locates mainly in the nucleus and it is (DEK1) released from macrophages. DEK2 is the alternatively spliced shorter form of DEK and it encodes a 341 amino acid protein lacking the residues 49-82 in DEK1. The data in the literature mainly focus on DEK1, the abundant isoform in the tissues. It has been shown that DEK1 is overexpressed in several cancer, it multimerizes and functions in a variety of cellular processes including the immune response, differentiation, proliferation, and DNA repair. Here we investigated whether DEK2 has similar characteristics with DEK1 in terms of dimerization, secretion and expression patterns in cancerous tissues.

## Materials and methods

We stably expressed the flag or myc tagged DEK isoforms to determine DEK1 and DEK2 in the medium of the cells and analyzed the medium using Western blot. myc or flag tagged DEK isoforms were also co-expressed and their interaction was investigated by co- immunoprecipitation. Finally, tissue cDNA array (RT-qPCR) was used to check the expression level of DEK1 and DEK2 in 8 different cancer tissues.

## Results

We found for the first time that DEK1 and DEK2 do not heterodimerize (in 293 cells) and both DEK1 and DEK2 are secreted from the bone marrow stromal cells HS27A. Both isoforms were expressed in both normal and cancerous tissue of colon, kidney, liver, lung, thyroid, prostate and ovaries. It was the same for the breast cancer whereas the normal breast tissue did not show DEK1 and DEK2 expression.

## Conclusion

Our results suggest that further functional studies focusing on the DEK2 might give insights into its distinct and/or DEK1-overlapping roles in cellular processes.

## Keywords

DEK isoforms,secretion,expression profile

## Acknowledgement

This study is supported by the Scientific and Technological Research Council of Turkey (TÜBITAK) Grants (216Z006 and 118Z765).

# Abstract: Metformin increases chronological aging by complicated mechanisms in Schizosaccharomyces pombe

**Authors:** Ceren SEYLAN, Irem KOÇ, Çagatay TARHAN

**Affiliations:** Istanbul University, Istanbul, Turkey

## Background/aim

Metformin is the most commonly used agent in the treatment of type-2 diabetes because of its potential to lower blood glucose. Besides, it has considerable anti-aging properties and increase longevity in various organisms and protects against heart diseases and cancer. The mechanism of action in these multifaceted effects is not clear and under debate. Therefore, it is important to clarify which genes and cellular pathways are affected by metformin when exerting its anti-aging effects. We aimed to find out some cellular mechanisms that may contribute to relationship between metformin and cell aging by using wild type and mutants of Schizosaccharomyces pombe.

## Materials and methods

After treatment of different doses of metformin with the cells, the effective dose was determined and the effects on chronological aging process, cell proliferation, intracellular oxidation, and other certain cellular parameters such as glucose consumption level were investigated. Besides, the effects of metformin on 6 different **S.pombe** mutants that are defective in DNA repair process were investigated.

## Results

Metformin increased the longevity of S.pombe cells by many folds in different conditions. Metformin treatment prevented lipid peroxidation by 5.86 times lower than the control group. Metformin-treated cells displayed a significantly lower extracellular glucose concentration compared to the control case. In addition to wild type, metformin restored the decreased-lifespan of 6 different *S.pombe* DNA repair mutants.

## Conclusion

According to our preliminary results metformin exerts its supportive effects via different cellular mechanisms including sugar metabolism, free radical balance and DNA repair systems. Further works are still in progress

## Keywords

Aging,Metformin,DNA repair, Schizosaccharomyces pombe

## Acknowledgement

This research is supported by Tubitak(The Scientific and Technological Research Council of Turkey) project number 1919B011804358.

# Abstract: Characterization of some probiotic bacteria in traditional and commercial pickle and kefir samples

**Authors:** Sevim KARAKAS, Yunus AKSÜT, Gürhan ÇIFTÇIOGLU, N. Özlem SANLI, Nazli ARDA

**Affiliations:** Istanbul University, Istanbul, Turkey

## Background/aim

Probiotics are a part of natural microbiota of fermented food, and used internationally in food and dairy products for many decades. Recently, studies on using the probiotics in preventing and treatment of specific diseases attract great attention. Probiotics have been tested in a large number of clinical studies for the treatment of various diseases. It is known that probiotics are abundantly found in various dairy and fermented products. Especially pickles and kefir are becoming more popular as probiotic sources in many countries. Although, there are several studies for the characterization of probiotic bacteria found in pickles or kefir around the world, studies on local ones are quite limited in our country. The aim of this study was to determine the probiotics in some traditional and commercial pickle and kefir samples.

## Materials and methods

*Lactobacillus plantarum*, *L. curvatus* and *L. brevis* have been targeted as common probiotic microorganisms, and MRS-agar has been used as selective medium in cultivation of these microorganisms. To identify probiotics in 3 kefir and 8 pickle samples, PCR method was performed with species-specific primers on the isolates grown in MRS-agar. DNA samples obtained from reference *Lactobacillus* species, and PCR products were analyzed qualitatively using agarose gel electrophoresis.

## Results

Considering the PCR results, *L. brevis* has been found in all kefir samples, in one commercial okra pickle and in one traditional cabbage pickle. *L. plantarum* has been found in commercial okra pickle and cabbage pickle. *L. curvatus* has been found in only one traditional cabbage pickle.

## Conclusion

This study exhibited that Turkish okra and cabbage pickles and kefir contain common probiotics, and would be considered as good sources for these selected bacteria.

## Keywords

Lactic acid bacteria,pickle,kefir,probiotic

## Acknowledgement

This study was funded by Scientific Research Projects Coordination Unit of Istanbul University, Project Number: 33011.

# Abstract: Analysis of RNA methylation proteins in different cancer types

**Authors:** Azime AKCAOZ, Ayse Hale GUCKIR, Bünyamin AKGUL

**Affiliations:** Izmir Institute of Technology, Izmir, Turkey

## Background/aim

Recent studies have shown that RNAs are subject to various chemical modifications, called epitranscriptomics, similar to epigenetic changes in DNA. Minor chemical modifications on RNA molecules have a huge impact on the fate of the modified RNA, leading to various molecular outputs, such as initiation and progression of cancer. Anumber of proteins are involved in this dynamic process, in addition to many yet to be identified. The aim of this study is to investigate the expression profiles of several RNA methylation proteins in various cancer types, such as breast, lung and colon cancer.

## Materials and methods

We first screened the RNA-seq profile of apoptotic HeLa cells treated with different drugs (cisplatin, TNF-a, doxorubicin and anti-FAS) to find out the relative expression levels of epitranscriptome-related genes. To confirm this data, cisplatin-induced HeLa cells were subsequently subjected to qPCR analysis. We repeated the same analyses in MCF7 (breast), A549 (lung) and CaCo2 (colon) cells to examine the expression of candidate genes across non-metastatic cancer cells of different origins. We further extended our analyses to healthy and metastatic cells of each cancer type to investigate the potential role of RNA modification proteins in cancer biology.

## Results

Interestingly, RBM15 was highly expressed in metastatic and non-metastatic cancer cell lines compared to healthy counterparts.

## Conclusion

These data suggest that RBM15 could be a potential biomarker or a target for monitoring cancer biology.

## Keywords

Epitranscriptomics,cancer progression,apoptosis

## Acknowledgement

This study was funded by TUBITAK (The Scientific and Technological Research Council of Turkey) (Project no: 217Z234)

# Abstract: A19 CRISPR-Cas9 Mediated Knock-out Screens in Childhood Brain Cancers

**Authors:** Göktug KARABIYIK, Ezgi Yagmur KALA, Alisan KAYABOLEN, Tunc MOROVA, Firat UYULUR, Nathan LACK, Tamer ONDER, Tugba BAGCI-ONDER

**Affiliations:** Koç University, Istanbul, Turkey

## Background/aim

Medulloblastoma is an embryonic cancer type and one of the leading causes of death for children among all cancer types. While chemotherapy and radiotherapy are commonly used treatment methods, survival rate is fairly low. Previously, mutated epigenetic regulators were identified for specific subtypes of medulloblastoma including demethylases, acetyltransferases and nucleosome remodelers. Identification of novel epigenetic signatures and vulnerabilities of medulloblastomas are important for understanding of the disease. To examine roles of epigenetic modifiers in medulloblastoma, we developed a CRISPR/Cas9-based Epigenetic Knock-Out Library (EpiKOL V2). This study mainly aims to identify epigenetic components, which are essential to medulloblastoma cells by using EpiKOL V2, which may lead to new therapeutic approaches.

## Materials and methods

EpiKOL V2 targets 800 epigenetic modifiers and for each gene, 10 gRNAs are present in the library in addition to essential genes and non-targeting gRNAs as controls. Targets included DNA-modifying proteins such as histone methyltransferases (HMTs), histone demethylases (HDMs), histone acetyl transferases (HATs), histone deacetylases (HDACs), and DNA methyl transferases (DNMTs). We performed a knock-out library screen with 1000X representation on a medulloblastoma cell line, Daoy with EpiKOL V2 to show essential epigenetic modifiers for Vincristine sensitive cells in addition to low dose Vincristine treated cells.

## Results

By next generation sequencing, we have shown that, gRNAs in EpiKOL V2 library are normally represented over cell populations. Following that, essentiality screen for control cells was completed and low dose and high dose drug screens for Vincristine sensitive Daoy cells were conducted.

## Conclusion

Identification of essential epigenetic modifiers for medulloblastoma may help us understand the disease and epigenetic mechanisms better. Similar screen will be conducted for other drug sensitive or resistant brain cancer types and ultimately novel therapeutic approaches can be developed.

## Keywords

Epigenetics,CRISPR/Cas9,Cancer

# Abstract: Association between NKILA and HSR in breast cancer cells

**Authors:** Mehmet Ali TIBATAN, Evren ONAY UCAR

**Affiliations:** Istanbul University, Istanbul, Turkey

## Background/aim

Heat shock proteins (HPSs) have vital role in breast cancer cell proliferation and some non-coding RNAs directly associates with these HSPs. A long non-coding RNA (lncRNA) NKILA (NF-κB interacting long non-coding RNA) negatively regulates NF-κB. Today, relation between HSPs and NF-κB is well known but role of NKILA in HSP/NF-κB association has not been shown yet. This possible NKILA-HSP/NF-κB association might involve some key aspects to reveal molecular mechanism of the heat shock response (HSR) in breast cancer. In this study, we aimed to show possible relation between NKILA and HSR in breast cancer cell lines.

## Materials and methods

Human breast cancer cell lines MCF-7 and MDA-MB-231 were used in this study. To inhibit the NKILA, cells were transfected with NKILA-siRNA by using Lipofectamine 2000^TM^. NKILA expression levels were measured with RT-qPCR, then HSP90α, HSP70, HSF1, NF- kB, β-catenin protein levels were determined by immunoblotting assay.

## Results

NKILA-siRNA silenced NKILA expression levels as 50% percentage. In NKILA silenced cells, HSP90α, HSP70 and HSF1 levels were decreased. Additionally, β-catenin and p50 also were altered in NKILA-siRNA group. We found an alteration in p50 and β-catenin levels with the HSPs.

## Conclusion

p50 alters HSF1, as a result this effects HSP70 and HSP90α expressions. Decrease in NF-κB and proteins of HSR proteins might be due to suggested correlation between NF-κB and HSPs. In other words, this alteration might result from cross-talk between HSR and NF-κB. Possible translocation of p65 which is effect p50 levels in cytosol has shown before and this can explain down-regulation of HSF1 under the exposure of NKILA-siRNA. Future studies are needed to clearly understand this pivotal role of NKILA in HSR.

## Keywords

Heat shock response,NF-κB interacting long non-coding RNA,breast cancer

## Acknowledgement

This study is supported by the Scientific Research Project Coordination of Istanbul University (Project-no: FDK-2017-25162).

# Abstract: Serotonergic neurons in abdominal ganglia control copulation in *Drosophila*

**Authors:** Yasemin YILMAZER, Baris Can MANDACI, Arzu CELIK FUSS, Masayuki KOGANEZAWA, Daisuke YAMAMOTO

**Affiliations:** 1 Bogazici University, Istanbul, Turkey; 2 Graduate School of Life Sciences, Sendai, Japan; 3 National Institute of Information and Communications Technology, Kobe, Japan

## Background/aim

Mating behavior of Drosophila melanogaster males comprise several discrete actions directed toward a potential mate, i.e., orientation, following, tapping, wing extension and vibration, attempted copulation and copulation. These stereotypic series of actions do not require learning, and are thus considered to be genetically programmed innate behaviors. P1 neurons in the brain initiate early parts of this mating behavior, such as following and wing display. However, relatively little is known about the neural mechanism controlling the late stage of mating behavior. Our group isolated a mutant, *platonic* (plt) that are characterized by the lack of copulation.

## Materials and methods

To further characterize the *platonic* mutant, we mapped *platonic* to the *scribbler* (*sbb*) locus by genetic complementation tests with several deficiency chromosomes, followed by molecular identification of the genomic region at which the mutagenic P-element insertion took place. To explore *sbb* gene function for mating behavior, RNA interference (RNAi) experiments were carried out by expressing genetically encoded RNAi constructs using the GAL4-UAS system. In this system, a GAL4 line that exhibits a desired expression pattern is used to activate a target gene specifically in a particular cell or tissue.

## Results

We focused on the male-specific circuitry composed of cells that express *doublesex* (*dsx*). The *sbb* knockdown by RNAi as driven by *dsx*-GAL4 led to a block in copulation. In order to narrow down the critical neurons, intersectional *sbb* knockdown experiments were performed by employing *dsx^FLP^* in combination with different GAL4 drivers that are specified by neurotransmitters. Mating success was markedly decreased in flies with a *Trh-GAL4* driver that specifies serotonin-producing cells.

## Conclusion

Eight serotonergic neurons with *dsx* expression in abdominal ganglia are critical for copulation in *Drosophila*. We are currently using the *trans*-Tango method to identify neural circuit of copulation from brain to abdomen.

## Keywords

*Drosophila melanogaster*,neural circuit,copulation

# Abstract: The effect of cholinergic receptors agonist /antagonists together with anticancer drug 5-FU in hepatocellular carcinoma

**Authors:** Ayse Melek TANRIVERDI BADEMCI, Onat Yalgin Kucuk, Banu AYDIN, Hulya CABADAK

**Affiliations:** Marmara University, Istanbul, Turkey

## Background/aim

Hepatocellular carcinoma (HCC) is the most common primary malignancy of liver. No drug has been identified for the specific treatment of anti-metastasis in HCC. The drugs used in the treatment of primary HCC tumors are similar to the ones used against tumor metastasis. Cisplatin, doxorubicin and 5-FU are members of these cytotoxic drugs. The EGFR pathway plays an important role in promoting HCC metastasis, although the mechanism remains unclear. A cholinergic system with acetylcholinesterase and acetylcholinic receptors has been detected in HCC although the function and molecular mechanism in hepatic carcinogenesis are unknown. The aim of this study is to investigate the role of EGFR-AChR signaling pathway on cell proliferation in HCC. In addition, this study showed that the exposure of human HepG2 hepatoma cells to palmitate results in apoptosis.

## Materials and methods

The HCC cells are cultivated in culture medium containing 10% FBS. The effect of MLA, 4DAMP, HC3, Eserine, PD153035, ACh, 5-FU and various combinations of these drugs on cell proliferation is investigated; the cells cultured in serum-free culture medium are studied after 1 night treatment with the required drug. BrDU and Western Blot methods have been used for cell-counting and molecular signal detection, respectively. Immunoblotting is performed using the antibodies against AChR7, M3 and actin.

## Results

Using the hepatoma cells as a model system, we analyzed the combined effects of EGFR, cholinergic receptors and 5-FU on cell proliferation and the apoptotic pathway.

## Conclusion

We suggest that the activation of M3R and EGFR pathway might be a potential mechanism for ACh induced cell growth. Additionally, M3AChR antagonism of 4-DAMP combined with 5-FU might affect the cell viability and apoptosis in steatotic and non-steatotic HepG2 cells.

## Keywords

Hepatocellular carcinoma,5-FU,M3R,AChR7

## Acknowledgement

# Abstract: Determination of the effect of curcumin on superoxide dismutase and catalase activity in tomato

**Authors:** Seda YASAR, Derya HATIPOGLU, Burcu KARPUZ, Ozgur CAKIR

**Affiliations:** Istanbul University, Istanbul, Turkey

## Background/aim

Tomato (*Lycopersicon esculentum*) is a plant which has high economic value. Curcuma longa is a member of Zingiberaceae family which contains curcumin components. Curcumin has shown to exhibit antioxidant bioactivity. Recent researches also showed that curcumin has anticancerogenic, anti-inflammatory, antiviral and antifungal activities besides antioxidant activity. Curcumin is mostly used in human health; however there are few studies about plants. In this study, the effect of curcumin on the Catalase (CAT) and Superoxide Dismutase (SOD) enzymes of the antioxidant mechanism in tomato were investigated.

## Materials and methods

Tissue culture was established from tomato seeds and then curcumin was applied 25 μM, 50 μM, 100 μM to tomato. The effect of curcumin on tomato was determined by enzymatic analysis. SOD was measured by the photochemical method. CAT enzyme activity was determined by UV spectrophotometry.

## Results

CAT enzyme activity was increased in tomato plants treated with 25 μM and 50 μM curcumin and decreased with 100 μM curcumin. SOD enzyme activity was decreased depending on curcumin concentrations. The highest reduction was observed in tomato plants treated with 100 μM curcumin. However, the least change was observed at 25 μM curcumin treatment.

## Conclusion

CAT enzyme activity increased at 25 μM and 50 μM, it decreased at 100 μM compared to control groups. This suggests that high concentrations of curcumin may cause toxicity to CAT enzyme activity. Ethanol was used during the preparation of curcumin stock. However, CAT reacts effectively with ethanol. SOD enzyme activity was decreased in tomato plants. It is suggested that there is a difference in enzymatic activity between wild and cultivated types according to literature.

## Keywords

Curcumin,antioxidant enzymes activity,plant cell culture,tomato (*Lycopersicon esculentum*)

## Acknowledgement

This research was supported by Istanbul University Scientific Research Projects Unit (Project number: FLO-2018-32774)

# Abstract: Plasma 7-ketocholestherol, cholestan-3β,5α,6β-triol and malondialdehyde levels in allergic asthma patients

**Authors:** Behnoush Nasr ZANJANI, Afshin SAMADI, Asli GELINCIK, Suna BUYUKOZTURK, Sengul BEYAZ, Incilay LAY, Nazli ARDA

**Affiliations:** 1 Istanbul University, Istanbul, Turkey; 2 Hacettepe University, Ankara, Turkey

## Background/aim

Prevalence of allergic asthma gradually increases in the world, depending on air pollution, climatic changes and other environmental stimulants. In allergic conditions, oxidative stress occurs as a result of activation of the immune system. Lipid peroxidation is a form of oxidative stress that involves oxidation of fatty acids by enzymatic and/or non-enzymatic reactions. Oxidation of cholesterol leads to the formation of a large number of oxidation products known as oxysterols. Oxysterols are potentially non-invasive biomarkers of oxidative stress in vivo. Determination of oxysterol and a product of lipid peroxidation, malondialdehyde (MDA) levels in the blood of patients with allergic asthma can provide information about the role of oxidative stress in the pathogenesis of allergic asthma, and may exhibit new approaches to the diagnosis and treatment processes.

## Materials and methods

Plasma levels of two oxysterols, namely 7-ketocholesterol and cholestane-3β,5α,6β-triol in total 120 asthma patients (90 females + 30 males) and 120 healthy controls (matched by age and sex) were quantified by LC-MS/MS (liquid chromatography-tandem mass spectrometry). Plasma MDA levels were analyzed by spectrophotometric method depending on the measurement of thiobarbituric acid reactive substances.

## Results

Plasma 7-ketocholesterol (39.4520.37 ng/mL) and cholestan-3β,5α,6β-triol (25.6110.13 ng/mL) levels in patients were significantly higher than those in healthy subjects (17.844.26 ng/ mL (p < 0.001) and 10.003.90 ng/mL, respectively) (p < 0.001). The plasma MDA level was found to be significantly higher in the asthmatics (4.981.77 nmol/mL) as compared to healthy controls (1.140.31 nmol/mL) (p < 0.001). Significant positive correlations were observed between oxysterol levels and MDA as well as total IgE levels in patients.

## Conclusion

Since lipid peroxidation products seem to be closely related to the complication of allergic asthma, plasma MDA, 7-ketocholesterol and cholestan-3β,5α,6β-triol levels may represent helpful and valuable biomarkers in the clinical monitoring of asthma patients.

## Keywords

Asthma,7-ketocholesterol,cholestan-3β,5α,6β-triol,LC-MS/MS,malondialdehyde

# Abstract: Comparison of early and proliferative stages of injury induced brain regeneration

**Authors:** Gokhan CUCUN, Yeliz DEMIRCI, Suhaib MOHAMMED, Guillaume HEGER, Irene PAPATHEODOROU, Gunes OZHAN

**Affiliations:** 1 Izmir Biomedicine and Genome Center, Izmir, Turkey; 2 Dokuz Eylul University, Izmir, Turkey; 3 European Bioinformatics Institute (EMBL-EBI), Hinxton, Cambridge, United Kingdom; 4 cole Centrale de Nantes, Nantes, France

## Background/aim

Regeneration is a complex and dynamic phenomenon that restores the original architecture of a tissue and is tightly regulated by various signaling pathways and the surrounding microenvironment. In contrast to the limited capacity of the adult mammalian central nervous system (CNS) to regenerate, adult zebrafish are able to successfully regenerate the CNS including the brain. Taking advantage of this capacity, we aim to unravel and compare the gene expression profiles at very early and proliferative stages of brain regeneration in a zebrafish model of traumatic brain injury.

## Materials and methods

To induce regeneration in the adult zebrafish brain, we applied traumatic brain lesion assay in one hemisphere of the telencephalon. Next, we dissected the injured telencephalons, had their RNA sequenced with next generation sequencing and analyzed the RNA-seq results. Finally, we validated a number of differentially expressed genes quantitatively by quantitative real-time PCR (qPCR) and qualitatively by immunohistochemistry on brain cryosections.

## Results

Our findings on differential gene expression analysis reveal that apoptosis related pathways and inflammatory response-related genes were predominantly expressed at early stage of brain regeneration while reactive gliosis associated pathways were upregulated in the proliferative phase. We also confirmed the differential expression of certain genes including GFAP, PCNA, NeuroD and Caspase-3, which are known to be induced during regeneration. Intriguingly, particular signaling pathways were found to be differentially regulated in the same direction in both stages.

## Conclusion

We believe that identification of genes that are differentially expressed at early and proliferative stages of brain regeneration will provide an in-depth understanding of brain regeneration mechanisms and will pave the way for development of new therapeutic strategies to enhance regeneration in the mammalian brain.

## Keywords

Neurodegenerative diseases,Apoptosis,Zebrafish,RNA-Seq

## Acknowledgement

TUBITAK 215Z365, EMBO Installation Grant

# Abstract: Oxidative stress and antioxidant defense in allergic asthma

**Authors:** Behnaz KARADOGAN, Sengül BEYAZ, Asli GELINCIK, Suna BÜYÜKÖZTÜRK, Nazli ARDA

**Affiliations:** Istanbul University, Istanbul, Turkey

## Background/aim

Asthma is a chronic and complex inflammatory disease with environmental and genetic factors that contribute to its development. There are evidences that reactive oxygen species, especially free radicals may play important roles in the development and severity of this disease. In this study, we aimed to investigate the levels of some oxidative stress markers, and antioxidant capacity in plasma or serum samples of patients with allergic asthma.

## Materials and methods

We analysed malondialdehyde (MDA), protein carbonyls (PC), reduced glutathione (rGSH) levels, ferric reducing antioxidant capacity and catalase activity in blood (plasma or serum) samples of patients with allergic asthma using spectrophotometric methods. Patients and healthy individuals with no history of allergy were included in this study (for each group n=120). Study subjects were divided into 3 groups, according to their asthma control test, as totally controlled (TC), partially controlled (PC) and uncontrolled (UC). Differences in variables were analysed by using appropriate statistical tests, including one-way ANOVA and unpaired t-test.

## Results

Our results showed that significant increase in plasma MDA and PC levels in all groups of patients compared to healthy subjects (p < 0.001), especially in UC ones. Besides, significant decrease in plasma rGSH (p < 0.001) level, plasma FRAP (p < 0.007) and serum catalase activity (p < 0.01) was detected in all patients. However, there were no significant differences for these parameters between TC, PC and UC patients.

## Conclusion

This research confirmed the presence of severe oxidative stress in asthmatic patients. Further attempts are needed in order to improve antioxidant capacity and supress molecular oxidations, such as lipid peroxidation and protein carbonylation for restoring life quality of allergic asthma patients.

## Keywords

Oxidative stress,allergic asthma,antioxidant defence,MDA,protein carbonyls

## Acknowledgement

This study was funded by Scientific Research Projects Coordination Unit of Istanbul University, Project Number: 30409.

# Abstract: Intramuscular drug application in zebrafish

**Authors:** Zeynep ÇINAR, Pervin DINÇER

**Affiliations:** Hacettepe University, Ankara, Turkey

## Background/aim

*Zebrafish* (*Danio rerio*) is a freshwater fish that constitutes a convenient vertebrate model for the study of many disorders because it has a genetic structure to humans highly similar. It became a useful tool in drug treatments recently. Drug delivery in zebrafish adults can realize with small molecules by means of water-borne exposure, use oral gavage or injection techniques. In this study, we delivered a drug directly wild-type zebrafish adults muscles via intramuscular injection to standardize the intramuscular drug administration protocol to be applied to zebrafish adults. Besides, we created muscular dystrophy phenotype in the zebrafish model the LGMD2R (MIM 615325/Orpha code: 363543) phenotype caused by DES mutation. So, we also will use this method in *desma* mutant zebrafish adults. Aims of this study are both setting up convenient intramuscular drug application algorithm for zebrafish adults and investigating whether relieving the muscle dystrophy phenotype caused by the mutation or not.

## Materials and methods

Toxicity experiments in zebrafish adults and embryos accomplished according to OECD guidelines for determining suitable drug dosage. Intramuscular injection applied on locally region by microinjection needle. There were four study groups (control, needle-soaked control, solvent control, drug-treated). RNA isolation performed from injection region. Then, using qPCR we analyzed effects on genes thought to be related.

## Results

There were no statistically significant differences between groups in wild-type adult zebrafish for the genes studied.

## Conclusion

Intramuscular injection with microinjection needle method was found applicable. Also, this method did not result in stress on adult zebrafish. Experiments in wild-type and *desma* mutant adults/embryos are being maintained and we propose that outcomes will be significative.

## Keywords

Zebrafish,drug application,molecular biology

## Acknowledgement

This study is funding by Hacettepe University Scientific Research Projects Unit (project ID:17472). Ethical permission has been obtained.

# Abstract: Investigation of the relationship between aspartame and Swi6 in *Schizosaccharomyces pombe*

**Authors:** Beste BAYRAK, Merve YILMAZER, Bedia PALABIYIK

**Affiliations:** Istanbul University, Istanbul, Turkey

## Background/aim

Aspartame (APM) is an artificial sweetener used in many products since 1981. While some studies have found carcinogenic effects of APM, some others could not detect any negative effect. In this research, our aim is to shed light on molecular mechanism of APM effects in a simpler model organism *Schizosaccharomyces pombe*. In several cancer studies, it has been found that HP1 expression was downregulated. Thus for the evaluation, human HP1 (heterochromatin protein 1) family ortholog of Swi6 is selected. Swi6 is a telomere, centromere, and mating-type locus binding protein for the regulation of heterochromatin structure.

## Materials and methods

To verify if the effects of APM is related to Swi6, *S. pombe* parental and *swi6*Δ cultures were added 1 mg/mL APM every day for 7 days to mimic real life consumption habits, then analyzed with viability and quantitative real time polymerase chain reaction (qRT-PCR) methods.

## Results

We detected in qRT-PCR data that while on first day APM caused a decrease in the expression of *swi6* in parental, expression levels for energy metabolism (*hxk2, fbp1, tup11, cyr1, cox4*) and stress response genes (*atf1, sod1, ctt1*) between parental and *swi6*Δ on first and seventh day differed significantly, depending on the effect of APM.

## Conclusion

According to the first day results, it has been seen that *S. pombe* parental strain has adapted to APM effects by activating the stress response pathway and *swi6*Δ did not present a meaningful response. Thus, we proposed that there may be a relationship between APM and Swi6. Nevertheless on the seventh day, the cumulative effects of APM showed that cells were in a trend of fermentation, which is an indication for carcinogenic activity.

## Keywords

Aspartame,cancer,epigenetics,*Schizosaccharomyces pombe*,Swi6

## Acknowledgement

This study was funded by Scientific Research Project Coordination Unit of Istanbul University. Project number: FYL-2018-30854.

# Abstract: The effect of nitric oxide on the tissue transglutaminase interaction with ITGβ-1, SDC-4 and PDGFR-β

**Authors:** Inci KURT-CELEP, Martin GRIFFIN, Dilek TELCI

**Affiliations:** 1 Yeditepe University, Istanbul, Turkey; 2 Aston University, Birmingham, UK

## Background/aim

Nitric oxide (NO), an extracellular second messenger, is responsible from the nitrosylation of 15 cysteine residues on TG2, which results in irreversible inactivation of the cross- link activity of the enzyme. TG2, activated by calcium, is the major enzyme that cross-links proteins by forming ε(γ-glutamyl) lysine peptide bonds. TG2 loses its "cross-linking" enzyme activity upon binding to the extracellular fibronectin and becomes a novel surface adhesion molecule that acts as a co-receptor for integrin β-1 (ITGβ-1), syndecan-4 (SDC-4) and platelet-derived growth factor beta- receptor (PDGFR-β). The aim of this study was to investigate the effect of NO on the interaction of TG2 with cell surface ITGβ-1, SDC-4 and PDGFR-β in Swiss 3T3 cells.

## Materials and methods

Swiss 3T3 cells induced to overexpress TG2 (IND) were treated with 50- 300 M of NO donor S-Nitroso-N-acetyl penicillamine (SNAP) for 48 hours and interaction between TG2 and ITGβ-1, SDC-4 and PDGFR-β was demonstrated with co-IP experiments.

## Results

While there was a decrease in the complex formation of TG2 with ITGβ-1 and SDC-4, an increase in the interaction of TG2 with PDGFR-β was detected in response to SNAP treatment.

## Conclusion

This study suggests that nitrosylation of TG2 affects its interaction with the cell surface binding partners ITGβ-1, SDC-4, and PDGFR-β.

## Keywords

Nitric oxide,tissue transglutaminase-2,ITGβ-1,SDC-4 and PDGFR-β

# Abstract: Impairing GTPase cycle of RAS via therapeutics: Alternative to SHP2 inhibitors used in cancer treatment

**Authors:** Metehan ILTER, Ozge SENSOY

**Affiliations:** Istanbul Medipol University, Istanbul, Turkey

## Background/aim

Recent studies have shown that tyrosyl phosphorylation of RAS stalls GTPase cycle. In this study, we aim to investigate the impact of phosphorylation on structure/dynamics of RAS and utilize this knowledge to prevent transforming properties of RAS mutants by means of small molecules that can be used as alternatives to SHP2 phosphatase inhibitors.

## Materials and methods

We performed molecular dynamics simulations on (non)-phosphorylated RAS for 23-microseconds. Possible binding pockets and pharmacophore groups were determined using Schrödinger package. The candidate molecules were searched in ZINC database. Trajectories were analyzed in terms of local/ global properties of systems.

## Results

Our results showed that phosphorylation increases flexibility of Switch I residues which are involved in RAF and GAP (GTPase-activating-protein) binding. Consequently, this caused displacement of Switch I from the binding pocket as a result of electrostatic repulsion between phosphorylated-Y32 and GTP (guanine-triphosphate), thus preventing interaction of RAS with both RAF and GAP which is consistent with experimental data. Moreover, phosphorylation also caused widening of the binding pocket which might explain increased rate of nucleotide exchange observed for phosphorylated RAS. Similarly, the flexibility of Switch II also increased which prevented interaction between the protein and GAP/GEF (guanine-exchange-factor). Importantly, we achieved candidate molecules that could displace Y32 from the binding pocket in G12D mutant of RAS isoforms.

## Conclusion

To the best of our knowledge, this is the first study where impact of phosphorylation on structural/dynamic properties of RAS isoforms has been explored. In light of these findings, we propose that candidate molecules have potential for impairing interaction between RAF and G12D RAS mutants by displacing Y32 from the binding pocket, thus inhibiting RAF/MEK/ERK signaling pathway.

## Keywords

RAS,SHP2 phosphatase inhibitor,phosphorylation,molecular dynamics simulations

## Acknowledgement

This study was supported by TUBITAK (The Scientific and Technological Research Council of Turkey), grant no: 1919B011701434.

# Abstract: Molecular mechanisms of PI3K isoform dependence in carcinogenesis

**Authors:** Sena ATICI, Gözde GÜVEN, Onur ÇIZMECIOGLU

**Affiliations:** 1 Bilkent University, Ankara, Turkey; 2 Middle East Technical University, Ankara , Turkey

## Background/aim

PI3K pathway plays crucial roles in cell proliferation, survival, motility. PI3K- related mutations (PTEN-loss, PIK3CA) frequently seen in cancers. p110β becomes prominent p110- isoform in PTEN-expression loss in some cancers. Understanding molecular mechanisms behind p110-isoform prevalence, we induced step-wise changes in MEFs, PC3s in attempt to switch PI3K isoform dependence from one to other.

## Materials and methods

Untransformed MEFs, PC3s (metastatic PTEN-null human prostate cancer) were used. Myristoylated-PI3Ks were used for continued pathway activation. PTEN expression was knocked-down using shPTEN constructs. Various clinically relevant PI3K inhibitors were used to assess isoform prevalence. GSE21543 dataset (microarray data for Pik3cb-overactivation induced prostate cancer samples) was analyzed with GEO2R. Crystal violet stainings were used for proliferation measurements, target genes' mRNA levels were quantified with qPCR.

## Results

MEFs are primarily p110α-dependent for growth. We tried to switch p110α dependence into p110β via inhibitor usage, changing PTEN expression status. shRNA-mediated downregulation of PTEN in MEFs was not sufficient for isoform switch. We aimed to re-express PTEN-wt, PTEN- C124S (catalytically inactive) in PC3s. PTEN re-expressing PC3s displayed decreased sensitivity to p110β/δ isoform inhibitors, p110α prevalence did not change. Further changes are required for complete isoform switch. To understand these secondary steps for p110β dependence, we analyzed GSE21543 dataset and determined 3 metabolism-related genes upregulated in p110β dependent manner; CREB3l4, AK4, SQLE. Sqle and Ak4 expression levels responded to p110β inhibition in MEFs. Terbinafine (SQLE inhibitor) and simvastatin (cholesterol synthesis inhibitor) treatments were done in MEFs expressing activated p110α/β alleles. p110β dependent MEFs more sensitive to inhibition of cholesterol synthesis.

## Conclusion

PTEN expression is not sole determinant for p110β dependence in MEFs and cancer cells. According to results, isoform dependence cannot be flipped in single step, preferentially regulated metabolic genes may instruct further changes in the cell to define prominent PI3K isoform.

## Keywords

PIK3CB,PTEN,PI3K

# Abstract: Investigating impact of phosphorylation on dynamics of farnesyltransferase: Understanding dynamic properties of enzyme in hyperinsulinemia

**Authors:** Hanife PEKEL, Mustafa GUZEL, Ozge SENSOY

**Affiliations:** Istanbul Medipol University, Istanbul, Turkey

## Background/aim

Farnesyltransferase (Ftase) is a dimeric metalloprotein which is responsible for addition of farnesyl group to C-terminus of proteins having CaaX motif, thus mediating their membrane attachment and activation. Among these, the members of RAS superfamily are crucial since their aberrant activation causes cancer. It has been shown that insulin stimulates phosphorylation of certain alpha-subunit residues of Ftase (S60-S62) which leads to increased enzymatic activity of Ftase, amount of farnesylated p21Ras, DNA synthesis and cell migration. Despite being widely targeted for therapeutic purposes, the impact of phosphorylation on structure/dynamics of Ftase has not been studied but might be important for drug discovery studies that target Ftase.

## Materials and methods

We performed a total of 12-microseconds molecular dynamics simulations using crystal structures, which represent the apo, farnesyl-pyrophosphate (FPP)-bound and FPP- and KRAS4B peptide substrate Ftase, respectively, to investigate the impact of phosphorylation on different states of enzymatic activity. The trajectories were analyzed using local/global system properties.

## Results

Our results showed that phosphorylation decreases flexibility of both FPP- and FPP/ peptide-bound enzyme; however, this difference is not pronounced in apo FTase. More interestingly, phosphorylation causes some structural rearrangements in the ligand binding cavity. Specifically, zinc atom is coordinated closer in both phosphorylated FPP- and FPP/peptide-bound Ftase than non-phosphorylated form of the enzyme. Also, key residues, which make up the hydrophobic cleft and coordinate FPP, display differences in their dihedral angle preferences between non- and phosphorylated Ftase.

## Conclusion

Considering that hyperinsulinemia is associated with cancer and type-II-diabetes and impact of insulin-mediated phosphorylation on the properties of the enzyme, these findings have the potential to improve structure-based drug-discovery strategies used for development of therapeutics that target Ftase.

## Keywords

Farnesyltransferase,phosphorylation,hyperinsulinemia,molecular dynamics

## Acknowledgement

Simulations were performed at TUBITAK (The Scientific and Technological Research Council of Turkey) High Performance and Grid Computing Center.

# Abstract: Association of single nucleotide polymorphisms (SNP)s in PLA2G2A gene with prostate cancer

**Authors:** Meltem Selen ONAL, Kaan OZTURK, Asif YILDIRIM, Nihan CELEBI-OLCUM, Dilek TELCI

**Affiliations:** 1 Yeditepe University, Istanbul, Turkey; 2 Istanbul Medeniyet University, Istanbul, Turkey

## Background/aim

Phospholipase A2s (PLA2)s are lipolytic enzymes hydrolyzing membrane phospholipids to form fatty acids and lysophospholipids. One of the key products is Arachidonic Acid (AA) that can be metabolized into prostaglandins and leukotrienes. sPLA2s have been associated with various cancers including prostate cancer. High level of sPLA2-IIA is correlated with high prostate tumor grade, tumor proliferation in primary site tumors. The expression of sPLA2-IIA both in mRNA and protein level increases more rapidly in metastatic tumors when compared to benign tumors. Furthermore, clinical studies on different single nucleotide polymorphism (SNP) of sPLA2- IIA indicated an association between the elevated serum levels of sPLA2-IIA in the cancer patients of afore mentioned diseases. This study aims to investigate the association of 5'UTR variant rs11573156 and the missense variants rs374105365 and rs965800220 on prostate cancer development.

## Materials and methods

Possible effects of missense variants were investigated through molecular dynamics simulations. For this purpose, mutated proteins were modelled in Cyrus Bench and simulations were run in Amber12 software. Results have been investigated in terms of H-bonding and surface electrostatic potential change. Genetic profile from a total of 267 blood samples (82 control and 184 PCa) was analyzed by polymerase chain reaction-restriction fragment length polymorphism (PCR-RFLP) method and Sanger sequencing.

## Results

Two missense variants, rs374105365 and rs965800220, show no effect on catalytic dyad interaction in terms of H-bond profile, while changes in amino acid side groups caused shift on surface electrostatics. The heterozygote allele of rs11573156 shows a 2-fold decreased risk for PCA while no significant correlation was detected for other SNPs.

## Conclusion

Our results showed that although there is a strong association between heterozygous genotype of rs11573156 (p < 0.05) and prostate cancer, there is no association between other missense variants and prostate cancer.

## Keywords

Prostate cancer,phospholipase A2,single nucleotide polymorphism

# Abstract: Detecting CNVs in Hereditary Cancer: Is there an alternative for MLPA?

**Authors:** Nihat Bugra AGAOGLU, Gizem ALKURT, Izzet Mehmet AKÇAY, Elifnaz ÇELIK, Jale YILDIZ, Kübra ERMIS TEKKUS, Sezin CANBEK, Gizem DINLER DOGANAY, Levent DOGANAY

**Affiliations:** 1 Umraniye Education and Research Hospital, Istanbul, Turkey; 2 Istanbul Technical University, Istanbul, Turkey

## Background/aim

Cancer is a disease that related to both genetic and environmental factors. Various genetic disorders may cause susceptibility to cancer as copy number variations (CNV). Many publications reveal the relationship between CNVs and the development of breast and colon cancer. MLPA (Multiplex Ligation-dependent Probe Amplification) is widely used to detect CNVs in breast and colon cancer cases. Recently with developing of the new algorithms in NGS data analysis, deletion/duplication detection can be performed in NGS data. In Umraniye Training and Research Hospital GLAB, molecular genetic tests have been performed for 750 breast cancer and 250 colon cancer patients to detect hereditary cancer syndromes. In the NGS data of 390 breast cancer and 125 colon cancer patients. CNV analysis was performed with Sophia DDM bioinformatics software algorithm. The obtained data were compared with MLPA analysis performed in 258 of them.

## Materials and methods

Peripheral blood samples obtained from the patients were tested by MLPA and NGS methods in germline samples for deletions/duplications after wet laboratory procedures in our laboratory.

## Results

NGS and MLPA data were compared in 258 patients. In 247 of these patients, no deletion/ duplication was detected in CNV analysis of NGS data. This result was consistent with the results of MLPA analysis in the same patients. In 7 patients, the same germline pathogenic variation was detected in both NGS and MLPA data. MLPA and NGS test results were discordant in 4 patients.

## Conclusion

Our study suggests that the reliability of the algorithms currently used in the detection of CNV from NGS data is not yet sufficient for routine diagnosis and that the results need to be confirmed by MLPA. Further studies are necessary in the field of developing algorithms.

## Keywords

Cancer,Copy Number Variation,Multiplex Ligation-dependent Probe Amplification,Bioinformatics

# Abstract: Investigation of autophagy mechanism in serum-starved BT-474 breast cancer cells

**Authors:** Mehmet CILIZLAR, Miray TURK, Ozge TATLI, Gizem DINLER-DOGANAY

**Affiliations:** 1 Istanbul Technical University, Istanbul, Turkey; 2 Istanbul Medeniyet University, Istanbul, Turkey

## Background/aim

Autophagy is an intracellular self-degradation mechanism responsible for maintaining cellular homeostasis. Beclin 1 and Bcl-2 are the main players in the regulation of autophagy/apoptosis decision. Bcl-2 associated athanogene 1 (BAG-1), a well-known binding partner of Bcl-2, is a pro-survival molecule involved in chaperone-assisted proteasomal degradation to tune proteostasis. Hence, BAG-1 might have a role in the autophagy-apoptosis toggle switch, in concert with Beclin 1 and Bcl-2. Here, we studied the starvation-induced autophagic response in BT-474 human breast ductal carcinoma cells, to understand how BAG-1 acts in autophagy/apoptosis decision.

## Materials and methods

BT-474 cells were cultured under serum starvation (0.1% instead of 10%) for 3 to 24 hours to induce autophagy. Total protein was extracted from serum-starved cells. The levels of autophagy marker proteins including LC3, ATG5-12 complex, Beclin 1, p-Beclin 1 (T119), Bcl-2, p-Bcl-2(S70) were analyzed by western blotting to monitor autophagic flux within the given time period. Further, BAG-1 levels were also assessed under serum starvation-induced autophagy. Statistical analyses were performed by GraphPad Prism version 8.0.1.

## Results

Autophagy markers were significantly upregulated in 3 to 6 hours of starvation in BT-474 cells. Serum starvation of the cells resulted in increased levels of p-Beclin 1 (T119), and conversely, decreased levels of p-Bcl-2 (S70) within this time period. BAG-1 levels, especially BAG-1S isoform, were started to increase after 6 hours of treatment.

## Conclusion

We conclude that autophagic response is induced between 3 to 5 hours of serum starvation in BT-474 cells. Disruption of Bcl-2/Beclin 1 interaction likely occurs through phosphorylation of Beclin 1. The increase in BAG-1 levels at the end of autophagic flux suggested that BAG-1, in particular BAG-1S isoform, may have a role in tipping the balance toward proteasomal activation.

## Keywords

BT-474 cells,Autophagy,BAG-1,Beclin 1,Bcl-2

# Abstract: Investigation of PI3K Functional Compensation via Activated Tyrosine Kinases

**Authors:** Melike DEMIR, Onur CIZMECIOGLU

**Affiliations:** Bilkent University, Ankara, Turkey

## Background/aim

Tyrosine kinases (TK) are essential Protein kinases, have crucial functions for cellular signaling. PI3K (Phosphoinositide 3-Kinase) is the most deregulated pathway in human cancers, activated via oncogenic Ras/receptor tyrosine kinases (RTKs). High levels of tyrosine phosphorylation associated with proliferation, which is regulated by PI3K.We are investigating whether tyrosine kinase contributes to growth compensation in response to p110α/β inhibition todemonstrate potential modes of resistance to PI3K targetted therapies.

## Materials and methods

We have TK library consisting of 73 kinases, have C-terminal dimerization tags promote trans-phosphorylation for constitutive activation. Genetically engineered MEFs, where PI3K signaling can be abrogated upon Cre-expression, was used for screening the library. To mimic p110α/β catalytic inactivation in MEFs, pharmacological inhibition that we employed p110α/β inhibitors BYL719/KIN193, has been used in addition to genetic knock-outs.For conditional knock- out of p110α/β, Adenoviruses expressing Cre-recombinase recognize lox-P sites inserted within first exons of PIK3CA-PIK3CB were used. Cell viability was assessed using crystal violet staining. To check anchorage independent growth and transformation ability of TKs of interest, soft agar growth assays were performed. We executed these experimental procedures for MEFs, MCF10A,RPE1-hTERT.

## Results

Only single TK-pool, consisting of PTK2B, PTK6, ZAP70, NTRK3 was able to rescue growth in MEFs upon p110α/β dual-loss. In proliferation assays, activated ZAP70 expression compensated growth deficiency in PI3K inhibited MEFs and significantly elevated growth rates of MCF10A. ZAP70 synergized with HRAS-G12 for transformation of MEFs on soft agar. ZAP70 expressing RPE1-hTERT cells proliferate at faster rate and became more resistant to p110α inhibition in comparison to controls.

## Conclusion

Besides having critical functions in immune system, ZAP70 may be potent driver of proliferation and transformation in epithelial as well as mesenchymal cells upon PI3K abrogation. Therefore studies on ZAP70 mediated growth in solid tumors is warranted.

## Keywords

PI3K,Signaling,TKs

## Acknowledgement

TUBITAK(The Scientific and Technological Research Council of Turkey, 117C040)

# Abstract: A37 The effects of *Maackia amurensis* leukoaglutinin on the alternative splicing events of human anaplastic thyroid cancer cell 8505C

**Authors:** Suna BEKTAS, Engin KAPTAN

**Affiliations:** Istanbul University, Istanbul, Turkey

## Background/aim

Many variations in the transcriptome play a role in the biogenesis, progression and metastasis of cancer cells. The splicing process generally deteriorates in cancer cells and this phenomenon is resulted in both functional and non-functional end products. Protein isoforms arising from alternative splicing of many genes have been associated with various aspects of cancer formation and progression. Our previous study has shown that *Maackia amurensis* leukoagglutin-II (MAL) decreases the tumorigenic and malignant characteristics of 8505C human anaplastic thyroid cancer cells. In this study, it was aimed to evaluate how differential splicing events are affected by MAL treatment to 8505C cells by using RNA-Seq data, which allows the identification of alternative splicing isoforms.

## Materials and methods

Differential alternative splicing events were analysed using Illumina next generation sequencing technology by using cDNA libraries obtained from total RNA isolates of 8505C cells treated with 0.25 μM MAL-II for 24 hours. We also performed functional enrichment analysis of the differentially spliced mRNA and they were grouped based on protein class using PANTHER resource.

## Results

Our results showed that MAL treatment significantly affected alternative splicing events of 832 mRNA. The differentially spliced mRNAs grouped in protein class category were mostly involved in nucleic acid binding which included 14.1% of the mRNAs.

## Conclusion

Overall findings have suggested that MAL treatment affects alternative splicing events in human anaplastic thyroid cancer cells. Differentially spliced 117 genes have indicated that the MAL treatment affects transcriptional processes in the cells. Consequently, the alternative splicing events can be an important factor in the suppression of malignant and tumorigenic properties of 8505C cells when considering our previous findings.

## Keywords

Thyroid cancer,lectin,alternative splicing,RNA sequencing

## Acknowledgement

This study was supported by Scientific Research Projects Coordination Unit of Istanbul University with the project number 32144.

# Abstract: The relationship between serum ApoM and lipid levels in restenosis patients

**Authors:** Gülçin ÖZKARA, Zahra JAVADOVA, Ezgi Irmak ASLAN, Onur KILIÇARSLAN, Özgür Selim SER, Ahmet YILDIZ, Oguz ÖZTÜRK, Hülya YILMAZ-AYDOGAN

**Affiliations:** 1 Istanbul University, Istanbul, Turkey; 2 Istanbul University Cerrahpasa, Istanbul, Turkey

## Background/aim

Apolipoprotein M (ApoM) is a novel anti-atherosclerotic protein which is present predominantly (~5%) in High-Density Lipoprotein (HDL) and in trace amounts (< 2%) in Low-Density Lipoprotein (LDL). ApoM plays important role in HDL metabolism via reverse cholesterol transport and has protective effects against LDL oxidation and atherosclerosis. Clinical studies revealing the association between ApoM and diseases are very limited. Therefore, we aimed to investigate the relationship between serum ApoM and lipid levels in restenosis patients and healthy controls. This is the first study investigating the association between serum ApoM and lipid levels in restenosis patients.

## Materials and methods

Forty-two restenosis patients and 36 healthy controls were included in our study. Serum ApoM levels were measured by ELISA method.

## Results

In the patient group, serum ApoM (p=0.022) and HDL-cholesterol (p < 0.001) levels were lower compared to healthy controls, whereas serum Triglyceride (TG) level was found to be higher (p=0.002). In addition, higher TG (≥150 mg/dL) and LDL-cholesterol (≥130 mg/dL) levels were associated with higher ApoM levels in the restenosis patient group (p=0.038 and p=0.041, respectively). However, this relationship was not observed in control group (p>0.05).

## Conclusion

Serum apoM level exhibited a significant, positive correlation not only with the HDL- cholesterol level, but also with the TG and LDL-cholesterol levels in patients with restenosis. These results indicated that the serum ApoM levels may affect the serum TG and cholesterol levels so that contributes to the development of restenosis.

## Keywords

Apolipoprotein M,HDL,LDL,cholesterol,Triglyceride,restenosis

# Abstract: Histone deacetylase inhibitors alleviate the expression levels of NOD-like Receptors in Eosinophil-like EoL-1 cells

**Authors:** Ilgin AKKAYA, Irem OZEL, Onur ESKIOCAK, Ceren CIRACI

**Affiliations:** Istanbul Technical University, Istanbul, Turkey

## Background/aim

Histone Deacetylase inhibitors (HDACi) have been reported to have complex immunomodulatory properties, particularly by negatively regulating the Toll-like receptor (TLR) pathways in different immune cells through histone modification and regulation of the chromatin structure. Nonetheless, the effects of HDACi in the regulation of eosinophilic, allergic functions and cytosolic NOD-like receptors (NLRs) are unknown. Henceforth, we aimed to investigate the immunomodulatory roles of HDACi in eosinophil-like cells and whether they have specific roles in shaping the functionality of NLRs.

## Materials and methods

To investigate the effects of HDACi in regulating eosinophilic functions and NLRs, EoL-1 cells were stimulated with valproic acid (VPA), romidepsin and sodium butyrate. mRNA levels of TLRs, NLRs, eosinophil specific anti-inflammatory receptor Siglec-8 and allergy- related Fc Epsilon receptors were analyzed by RT-qPCR. Protein levels of NLRs and TLRs were determined by immunoblotting. Flow cytometry experiments were performed to analyze the surface expression of Siglec-8 and Fc epsilon receptors. To determine the effects of HDACi on IL-1B and IL-6, ELISA experiments were utilized.

## Results

HDACi treatment drastically downregulated the expression of NLR proteins at mRNA and protein levels. This pattern was not observed in TLR protein expression although TLR mediated IL-6 secretion declined after HDACi treatment. Interestingly, HDACi treatment did not alter the surface expression of Siglec-8 or the Fc Epsilon receptors.

## Conclusion

This study demonstrated the specific downregulation of NLRs in EoL-1 cells while TLR expression was not diminished. Furthermore, HDACi reduced the secretion of IL-6, a downstream product of the TLR pathways. Our data present possible targets in the regulation of NLR expression which can lead to the characterization of new molecular mechanisms of eosinophilic and allergic functions.

## Keywords

Eosinophils,HDACi,NOD-like receptors

## Acknowledgement

This study was funded by 115Z819 TÜBITAK project and 40713 ITÜ BAP project.

# Abstract: Investigation of PD-L1, miR-140 and miR-34 expression by colorectal cancer cells following doxorubicin treatment

**Authors:** Noel Mopouguini NABA, Gonul SEYHAN, Dogus ALTUNOZ, Mehmet CALCI, Batu ERMAN, Ayca SAYI-YAZGAN

**Affiliations:** 1 Istanbul Technical University, Istanbul , Turkey; 2 Sabanci University, Istanbul, Turkey

## Background/aim

Doxorubicin (DOX) is an anthracycline drug that is used for the treatment of various cancers but not for colorectal cancer (CRC) because of its resistance and severe side effects depending on the required high doses. The programmed death ligand-1 (PD-L1), a T lymphocyte inhibitory molecule, is highly expressed on tumor cells and plays a role in immune escape. This study aims to investigate the level of PD-L1, miR-140 and miR-34a expressions in DOX-treated CRC and breast cancer (BCR) cells.

## Materials and methods

RKO, Caco-2 and HCT-116 CRC and MDA-MB-231 BRC cells were treated with different concentrations of DOX (0.1, 0.5, 0.7, 1 and 1,5 μM) for 18 h. PD-L1 expression was determined by flow cytometer and qRT-PCR. The expression of miR-140 and miR-34a in HCT- 116 and MDA-MB-231 cells were assessed by qRT-PCR following DOX treatment.

## Results

DOX-dependent PD-L1 expression was elevated in RKO, Caco-2 and HCT-116 CRC cells with different concentrations of DOX while it is significantly decreased in MDA-MB-231 BRC cells. Moreover, PD-L1 expression was inversely correlated with miR-140, but not with miR-34a, in HCT- 116 cells. On the other hand, the expression level of both miR-34a and miR-140 was significantly induced in MDA-MB-231 cells.

## Conclusion

Our data suggest that PD-L1 expression is increased by DOX-treatment in CRC cells, including RKO, Caco-2 and HCT-116 while it is downregulated in MDA-MB-231 BRC cells. In addition, DOX treatment leads to downregulation of miR-140 expression in HCT-116 and in opposite upregulation in MDA-MB-231 cells. Overall, these findings suggest a role for PD-L1 in therapy to improve the efficiency and decrease DOX-related toxicity.

## Keywords

PD-L1,doxorubicin,miR-140,miR-34a

## Acknowledgement

This study was supported by Istanbul Technical University, Department of Scientific Research Projects (ITU-BAP) (Project # 39555).

# Abstract: End-binding 3 protein alterations in an in vitro spinal muscular atrophy model

**Authors:** Dila KOYUNOGLU, Gamze BORA, Çagdas Devrim SON, Hayat Erdem YURTER

**Affiliations:** 1 Hacettepe University, Ankara, Turkey; 2 Middle East Technical University, Ankara, Turkey

## Background/aim

Spinal muscular atrophy (SMA) is a rare neurodegenerative disease which is caused by mutations in *>Survival of motor neuron 1* (*SMN1* ) gene. Absence of SMN protein leads to cytoskeleton defects, especially in neurons, due to dysregulations in regulatory proteins. Our previous results showed impaired microtubule stability in SMN depleted cells and also alterations in some microtubule associated proteins, including microtubule-associated protein 1B (MAP1B). MAP1B affects the activity of microtubule plus-end protein, end-binding 3 (EB3), via regulating its microtubule binding. Therefore, in this study, we investigated the expression and localization of EB3 proteins in an in vitro SMA model.

## Materials and methods

SMN gene expression was knocked down by siRNA in murine motor neuron line cell line, NSC34. EB3 protein level and localization were analyzed by Western blot and immunofluorescence stainings, respectively. Quantitative microscopic analysis was performed to analyze the number and area of EB3 comets on dynamic microtubule network.

## Results

We detected a significant downregulation in EB3 protein level in SMN knock down cells. We also found that comet numbers were significantly increased at the beginning of neurites, however, the area of one comet on dynamic microtubules was reduced.

## Conclusion

Our results suggested a dysregulation in EB3 protein dynamics in the absence of SMN protein. Studies are ongoing to understand the possible link between EB3, MAP1B and their effects on microtubule stability in SMA.

## Keywords

Spinal muscular atrophy,microtubule,end-binding 3

## Acknowledgement

This study was supported by Hacettepe University Scientific Research Projects Coordination Unit (Project number: TYL-2018-17434).

# Abstract: Identification of resistance mechanisms against cytotoxic drugs using genome-wide CRISPR/ Cas9 Knock-out screening approach

**Authors:** Zeynep Hazal Yilmaz, Zeynep Tokcaer-Keskin, Nazli Kesikin Toklu, Emre Deniz

**Affiliations:** Acibadem Mehmet Ali Aydinlar University, Istanbul, Turkey

## Background/aim

Cancer is one of the highest rated disease on the values of mortality ratio. Among several types of treatment options, chemotherapy is still the most used. Cytotoxic drugs are widely used against cancer during chemotherapy. However, the drug efficacy might be interfered when cancer cells develop resistance to these drugs. This resistance may occur by several different reasons such as cancer heterogenity, molecular changes, drug efflux, etc.

## Materials and methods

In our experiments, we used doxorubicin as a model drug. Dox is a topoisomerase II poison that causes DNA double strand breaks. Thus, on fast dividing cells, such as cancer cells, dox has a cytotoxic effect because cells cannot keep up repairing these breaks. We planned to systematically knock out almost each protein coding gene in human MDA-MB-231 cancer cell line in order to screen the contribution of their individual absences in developing resistance to doxorubicin.

## Results

We isolated their genomic DNA, PCRed out the integratad viral genome sequences. Later we sequenced the PCR product on next generation sequencing platform and the results are in the progress of analysing the data.

## Conclusion

By this, we will identify the mutant genes that confer resistance to cancer cells. The products of the identified genes might have potential to be targets for new diagnostic tools and therapy approaches for cancer in clinics.

## Keywords

CRISPR/Cas9,gene editing,drug resistance,breast cancer,chemotherapy

## Acknowledgement

This project is supported by TUBITAK 1003, grant number: 216S404

# Abstract: The role of autophagy in midgut stem cells of *Bombyx mori*

**Authors:** Busra GUNAY, Ebru GONCU

**Affiliations:** Ege University, Izmir, Turkey

## Background/aim

Autophagy is thought to be a critical mechanism for providing self-renewal, proliferation and differentiation of stem cells. *Bombyx mori* midgut enters remodelling process during larval-pupal metamorphosis. Larval midgut stem cells undergo growth, proliferation, and differentiation, which leads to the formation of the pupal epithelium. In this study, the role of autophagy mechanism in stem cells forming pupal midgut was investigated.

## Materials and methods

In order to prevent autophagic activity, the larvae were administrated chloroquine by injection in two different doses, 1 mg and 3 mg, on days 7 and 8 of the fifth instar. Stem cells were isolated from midgut in every 24^th^ hrs. The expression levels of autophagy-related genes ATG8 and ATG12 in stem cells were determined by qRT-PCR. The amount of lysosomal enzyme acid phosphatase was measured spectrophotometrically. Proliferation and differentiation of stem cells were also investigated morphologically. The findings were evaluated comparatively with the control groups.

## Results

Metamorphosis did not occur in silkworms treated with chloroquine. In the control groups, ATG8 and ATG12 mRNA levels increased on days 8 and 9, and continued to decrease until 24th hour of pupa. On day 8, except for 3 mg of chloroquine treated group, significant increases in the expression of the genes studied in the other treatment groups were observed. Acid phosphatase enzyme increased in stem cells immediately after application of chloroquine. In the results of morphological analysis, chloroquine applications prevented differentiation of stem cells and healthy organization of pupal epithelium.

## Conclusion

As an autophagy inhibitor, chloroquine regulates the breakdown of autophagic protein in the lysosomal system. In this study, after chloroquine application, increasing expression of autophagy-related genes, increasing acid phosphatase enzyme amount and abnormal morphological findings, indicated the role of autophagy in the proliferation and differentiation of midgut stem cells.

## Keywords

*Bombyx mori*,midgut,stem cell,autophagy

# Abstract: CRISPR/Cas9 technology: Functional characterization of single amino acid variants

**Authors:** Betül PIR, Sebiha CEVIK, Oktay I. KAPLAN

**Affiliations:** Abdullah Gul University, Kayseri, Turkey

## Background/aim

Determining the biological impact of the ever-increasing number of human genetic variants is presently challenging. The functional inference computational tools are apparently not adequate enough for predicting functional consequences of missense variants, requiring experimental evaluation of these variants. CRISPR/Cas9 technology is a cost-effective and a rapid method to introduce a single nucleotide change to any part of genome. By employing CRISPR/Cas9 technology, we investigated the biological functions of two variants (P726A and G704S) in short-rib thoracic dysplasia associated IFT140 (Intraflagellar Transport 140) gene in *C. elegans*.

## Materials and methods

ConVarT (www.convart.org), a new tool developed by our research group, was used to find two conserved variants between human and *C. elegans* with high pathogenic score. Homozygous mutants carrying a single amino acid change (IFT140^P726A^ or IFT140^P726A^) in *C. elegans* were generated using the CRISPR/Cas9 system. Dye filling assay and microscopic analysis were used to assess functional effects of these variants on the structure of cilia.

## Results

Although both variants are predicted to be pathogenic by functional prediction programs, SIFT and PolyPhen-2, we have found that IFT140^P726A^, but not IFT140^G704S^ mutant phenocopies IFT140 null mutant for the ciliary accumulation of IFT proteins and short cilia. The IFT140^P726A^ phenotype was fully rescued by introducing wild type IFT140.

## Conclusion

Pathogenicity prediction tools provide important insight into the functional implications of possibly disease-causing variants but experimental validations are needed to comprehend true nature of a variant.

## Keywords

CRISPR,*C. elegans*,variants,rare diseases

## Acknowledgement

We thank Pei Zhao and Yahong Kang for their technical assistance. This work is supported by the Scientific and Technological Research Council of Turkey (TÜBITAK), Grant No: SBAG- 118S552 and Grant No: KBAG- 118Z471.

# Abstract: Understanding the basis of Nek1-linked ALS pathogenesis and development of a potential therapeutic strategy

**Authors:** Bahriye ERKAYA, Harun ÖZTÜRK, Umut SAHIN

**Affiliations:** Bogazici University, Istanbul, Turkey

## Background/aim

Amyotrophic lateral sclerosis(ALS) is a neurodegenerative disease(ND) with dismal prognosis and driven by mutations in multiple genes. Recently, a new ALS-related gene(NIMA- related kinase1, NEK1) was identified but the cellular mechanism of disease pathogenesis is unknown. We hypothesize that NEK1 mutants aggregate, creating cytotoxicity in similar manner as other NDs. In cells, aggregation-prone proteins can be eliminated by the ubiquitin proteasome system. Recent studies also implicate SUMO (Small Ubiquitin-like Modifier) peptides in protein degradation. In particular, SUMOylation can facilitate ubiquitination and proteasome recruitment. PML(Promyelocytic leukemia) nuclear bodies(NB) are subnuclear protein-based structures that facilitate SUMOylation and degradation of various proteins. This project aims to elucidate how NEK1 mutations result in ALS pathogenesis and find possible therapeutic intervention for NEK1- related ALS.

## Materials and methods

We used HEK293 cells transiently expressing a GST-tagged wild type(wtNEK1) or ALS-linked truncated mutant form(tNEK1) of NEK1. Subcellular localization and aggregation characteristics were determined by immunofluorescence with GST antibody. Aggregation propensity and protein solubility were further analysed by biochemical extraction methods. Post- translational modifications of wtNEK1 and tNEK1 were studied by immunoprecipitation followed by immunoblotting.

## Results

We show that the ALS-linked tNEK1 experiences massive loss of solubility and forms intranuclear aggregates with cytotoxicity. In nucleus, unaggregated tNEK1 is recruited in PML NBs and becomes hyper-SUMOylated. Interferon alpha(IFN), which induces PML expression, PML NB biogenesis and tNEK1 recruitment in PML NBs, also induces PML NB-dependent hyper- SUMOylation, ubiquitination and degradation of toxic tNEK1.

## Conclusion

We hope to have a better insight into a highly complex disease as ALS. To identify the cellular basis of NEK1-linked ALS, we showed that tNEK1 forms toxic cellular aggregates, which can be reversed by IFN treatment that selectively targets tNEK1 for destruction via PML NB-dependent SUMOylation*.

## Keywords

ALS,SUMOylation,targeted therapy

## Acknowledgement

Türk Patent Basvuru No.2019/03865 European Molecular Biology Organization (EMBO).

# Abstract: Transient transfection and expression of ATM

**Authors:** Serap PEKTAS

**Affiliations:** Recep Tayyip Erdogan University, Rize, Turkey

## Background/aim

Ataxia telangiectasia-mutated (ATM) is a Ser/Thr protein kinase that is involved in sensing DNA double strand breaks and activation of the repair mechanism. ATM signals to proteins involved in DNA repair, cell cycle arrest, and apaoptosis by phosphorylating downstream targets such as p53, Chk1/Chk2, H2AX, BRCA1, etc. ATM consists of 3056 residue, due to its size difficult to express with a high protein expression yield. This study aims to determine and compare the ideal transfection method and cell line for transient transfection of ATM.

## Materials and methods

All chemicals were purchased from commercial vendors and used without further modification, except the pcDNA3.1(+)Flag-His-ATM wt, it was a gift from Michael Kastan (Addgene plasmid #31985). The plasmid DNA produced in Stbl2 cell strains and isolated using Thermo Scientific transfection grade DNA isolation kit. Two different transient transfection methods used, calcium phosphate coprecipitation technique and Polyethylenimine (PEI) transfection technique and transfections performed with one adherent cell line and one suspension-adapted cell line, Flp-In 293, and Expi293F, respectively. The efficiency of the transfection was tested using ATM kinase- specific ELISA kit.

## Results

PEI transfection method showed 2.5 times higher protein expression yield when compared to the calcium phosphate coprecipitation method on Flp-In 293 cell line. Besides, PEI method also displayed 8 times higher protein expression on Expi293F cells than Flp-In 293 cells.

## Conclusion

The obtained results indicated that ATM kinase plasmid used in this study expressed better in Expi293F cells, and PEI transfection method was more efficient for transient transfection of both cell lines.

## Keywords

ATM,Transient Transfection,DNA Double Strand Break (DSB)

## Acknowledgement

This work supported by The Scientific and Technological Research Council of Turkey (TUBITAK, Grant Number: 116Z360)

# Abstract: Characterization of CRISPR/Cas9 edited *KCNQ1* gene variants in human induced pluripotent stem cell-derived cardiomyocytes

**Authors:** Tugçe TORUN, Sevilay SAHOGLU GÖKTAS, Yusuf Enes KAZCI, Esra ÇAGAVI

**Affiliations:** Istanbul Medipol University, Istanbul, Turkey

## Background/aim

Human induced pluripotent stem cells derived cardiomyocytes (iPSCs-CMs) can be used to model cardiac arrhythmias such as Long QT syndrome type 1 (LQTS-1). Previously, we have targeted heterozygous c.965 C/T mutation in *KCNQ1* gene in iPSCs obtained from LQTS-1 patients by using CRISPR/Cas9 system. Random deletions were detected after editing in 3 iPSC clones carrying different *KCNQ1* variants of which were repaired by NHEJ. We aimed to determine molecular and electrophysiological characteristics of uniquely edited iPSCs-CM cultures.

## Materials and methods

CRISPR/Cas9 edited mixed sequences of *KCNQ1* gene were separated by TA subcloning. We determined *KCNQ1* variants by Sanger sequencing and characterized gene expression by qRT-PCR, Western Blot and immunocytochemistry. Effects of *KCNQ1* variants on electrophysiological behaviour of iPSCs-CMs were explored via Ca+2 imaging and field potential recordings.

## Results

Gene expression analysis showed that pluripotency of iPSCs were not affected by CRISPR/ Cas9 editing. Sanger sequencing revealed mutations that arose from CRISPR/Cas9 editing resulted in frame shifts in the *KCNQ1* gene sequence while producing an early stop codon in one variant. Expression of cardiac specific genes in genome edited clones did not show statistically significant differences. Importantly, Ca+2 imaging of iPSCs-CMs demonstrated prolonged QT intervals and early after depolarisation (EAD) defects with truncated *KCNQ1* expressing cultures compared to healthy cultures, reminiscent of clinical phenotype. Furthermore, administration of isoproterenol to the iPSCs-CMs increased beating rate.

## Conclusion

Reminiscent of clinical phenotype, LQTS-patient derived and CRISPR/Cas9 edited iPSCs-CMs demonstrated prolonged QT intervals and EAD defects with mutant *KCNQ1* expressing cultures. These electrophysiological findings demonstrated genotype-phenotype correlation in LQTS iPSC-CMs confirmed by CRISPR/Cas9 editing and developed a new model to study disease mechanism.

## Keywords

Long QT Syndrome,iPSCs-Derived Cardiomyocytes,CRISPR/Cas9 system

## Acknowledgement

TÜBITAK under 1003 projects no: 213S192 and Istanbul Medipol University BAP Committee by project no: 2018/17.

# Abstract: Interactions between mineralocorticoid and estrogen receptor signaling in T47-D breast cancer cell line

**Authors:** Damla GÜNES, Bircan ÇOBAN, Seniye TARGEN, Olivier STAUB, Özlen KONU

**Affiliations:** 1 Bilkent University, Ankara, Turkey; 2 University of Lausanne Lausanne, Switzerland

## Background/aim

Mineralocorticoid receptor (MR), a steroid hormone receptor, and its ligand Aldosterone (ALDO) have been gaining attention in cancer research, recently. Another steroid receptor, Estrogen Receptor (ER), has been shown to interfere with MR signalling when activated by Estrogen (E2). Herein, we aimed to investigate the role of MR-ALDO signalling and reveal its possible crosstalk with ER-E2 signalling in T47D cell line.

## Materials and methods

Breast cancer cell lines of different subtypes were screened for MR by RT-qPCR and Western Blotting. MCF7, T47D, MDA-MB-231 cells with different MR and ER status were chosen for ALDO and/or E2 administration, and known members of MR signalling were quantified at mRNA and/or protein levels. Combinatorial effect of these hormones on cell viability and proliferation was tested by MTT assay together with expression in T47-D cells. Association between MR expression and ER status on breast cancer patient survival were investigated by Kaplan-Meier Plots.

## Results

Different MR expression levels were detected in a panel of eight breast cancer cell lines. Known components of MR signalling such as SGK1, NEDD4-2 and subunits of ENaC were found to respond differentially to ALDO or E2 exposure in different cell lines. Additionally, ALDO and E2 exhibited opposing effects, i.e., anti-proliferative and proliferative, respectively, in T47D cells (ER+MR+) which was further studied for crosstalk. Furthermore, high MR expression was shown to be a significant cue for relapse-free survival for ER+ breast cancer patients.

## Conclusion

Possible crosstalk between MR and ER signalling pathways needs to be tested further, functionally and mechanistically, in T47D cells. A better understanding of this crosstalk can help uncover some untouched aspects in breast cancer signalling.

## Keywords

Breast cancer,Mineralocorticoid Receptor,Aldosterone,Estrogen

## Acknowledgement

This study was funded by The Scientific and Technological Research Council of Turkey (114S226) and COST ADMIRE.

# Abstract: In vitro investigation of the interactions between Raf kinases and pro-survival BAG-1 in breast cancer

**Authors:** Miray TURK, Ozge TATLI, Ecenur CEBI, Zeynep SAHIN, Gizem DINLER-DOGANAY

**Affiliations:** 1 Istanbul Technical University, Istanbul, Turkey; 2 Istanbul Medeniyet University, Istanbul, Turkey

## Background/aim

BAG-1 is a pro-survival molecule, which protects the cells from apoptotic stimuli and is frequently upregulated in human malignancies. C-Raf and B-Raf serine/threonine kinases, known interaction partners of BAG-1, are involved in MAPK pathway that governs proliferation and cell survival. The interaction of BAG-1 with C-Raf and B-Raf sets a critical role in the prolonged survival of cancer cells. However, the exact role of BAG-1 in phosphorylation-dependent Raf activation cycle remains unknown. Herein, we aim to probe phosphorylation-dependency of the interactions between Raf kinases and BAG-1 in breast carcinoma.

## Materials and methods

His_6_-tagged BAG-1S, BAG-1L, C-Raf and B-Raf genes are cloned into mammalian expression vector. To mimic the phosphorylated and active state of Raf kinases, S446D and S338D substitutions were introduced into B-Raf and C-Raf, respectively. MCF-7 cells were transfected with His6-tagged constructs and MTT assay was performed to screen the effect of proteins on viability. Cell lysates were used in His-pull down assays. Non-mutated Raf kinases were subjected to phosphatase treatment to fully inactivate kinases.

## Results

To investigate phosphorylation-dependency of BAG-1/Raf kinase interactions, an aspartate substitution mimicking phosphorylated serine was introduced at residues S-338 and S-446 for C-Raf and B-Raf, respectively. The interactions of active/inactive Raf kinases with small/large isoforms of BAG-1 were investigated through His-pull down assays. His-pull down experiments showed that while only BAG-1L interacts with fully inactivated C-Raf and B-Raf, both BAG-1L and BAG-1S isoforms interact with phosphomimeticly active forms of kinases.

## Conclusion

The isoform-specific interaction of BAG-1 with activated and inactivated forms of Raf kinases will contribute to our understanding the role of BAG-1 in MAPK pathway for the selection of druggable sites for inhibition of interactions between MAPkinases and BAG-1 to block the prolonged survival of cancer cells.

## Keywords

BAG-1,C-Raf,B-Raf

## Acknowledgement

This work is supported by TUBITAK (Project No:117Z848).

# Abstract: NLRP7-BMP4 axis is involved in human early embryogenesis

**Authors:** Aybüke GARIPCAN, Burcu ÖZÇIMEN, Ilke SUDER, Volkan İLKER, Tamer ÖNDER, Nesrin ÖZÖREN

**Affiliations:** 1 Bogaziçi University, Istanbul, Turkey; 2 Koç University, Istanbul, Turkey

## Background/aim

NOD-like receptor family pyrin containing protein 7 (NLRP7) is a member of cytosolic pattern recognition receptors, participating in inflammasome complex formation. Mutations in the primate-specific maternal-effect gene have been rather associated with biparental complete hydatidiform mole (HM), gestational trophoblastic disease exhibiting trophoblast hyperproliferation with no embryo formation although how NLRP7 deficiency contributes to HM pathogenesis remained obscured.

## Materials and methods

So as to investigate the role of NLRP7 in HM, fibroblasts from a patient carrying compound heterozygous NLRP7 mutations have been reprogrammed to induced pluripotent stem cells (iPSCs). Using a cocktail containing BMP4 and inhibitors of activin/Nodal and FGF signaling (BAP cocktail), trophoblasts were differentiated from iPSCs. The loss of pluripotency and gain of trophoblast markers were verified by qPCR, western blotting, immunofluorescence and RNA sequencing. Trophoblast differentiation was conducted using no BMP4 (AP cocktail) and confirmations were performed as mentioned above. Activation of BMP4 pathway was examined by western blotting and ELISA.

## Results

Trophoblast differentiation from HM iPSCs were accelerated compared to WT cells upon exposure of BMP4 and inhibitors of activin/Nodal and FGF signaling. HM iPSCs could differentiate into trophoblasts even with its endogenous BMP4 (exposure to AP cocktail). BMP4 pathway in HM iPSCs is activated earlier upon AP cocktail.

## Conclusion

NLRP7 deficiency predisposes iPSCs to trophoblast lineage commitment via BMP4 pathway, suggesting a critical role for NLRP7-BMP4 in human early embryogenesis.

## Keywords

Hydatidiform mole,NLRP7,early embryogenesis,BMP4

## Acknowledgement

TUBITAK 112S115, TUBITAK 217Z131

# Abstract: In vivo assessment of pluripotent stem cell derived cardiomyocytes in rhythm disorders

**Authors:** Gizem ÖRS, M. Ugurcan TURHAN, G. Ilayda SÖZTEKIN GÖRGÜLÜ, Esra ÇAGAVI

**Affiliations:** Medipol University, Istanbul, Turkey

## Background/aim

Long QT syndrome (LQTS) is the most common hereditary form of irregular heart rhythm disorder, also known as arrhythmias. LQTS Type-1 is characterized by a prolongation of the QT interval due to a mutation in K+ channel-coding *KCNQ1* gene. In the context of this study, therapeutic potential of cell transplantation in arrhythmia was tested in LQTS Type-1 transgenic mouse model for the first time in the literature.

## Materials and methods

Wild-type embryonic stem cell derived cardiomyocytes were injected into myocardium of transgenic mouse hearts. To understand the electrophysiological effects of the cell transplantation, electrocardiographic (ECG) recordings of operated mice (n=10) taken before and 7 days after surgery were compared to the ECG measurements of SHAM controls (n=10). Immunohistochemical analysis (IHC) was performed 7 and 14 days after the operation to analyze integration of transplanted cells in the heart.

## Results

Our findings revealed that 50% of operated transgenic mice have shown shortened QT intervals, suggesting an ameliorated disease phenotype. From all operated mice, 20% have shown an increase and 30% showed no significant difference before and after treatment. IHC analysis showed transplanted wild-type cardiomyocytes expressing cTnT protein were integrated to the myocardium.

## Conclusion

Through this study, cell therapy approach were tested for the treatment of LQTS Type 1 for the first time in the literature and therapeutical effects of cell transplantation are confirmed. Together with further preclinical and translational studies on large animal models, cell transplantation have the potential to develop as a novel therapeutic approach for arrhythmia treatment.

## Keywords

Arrhythmia,cell transplantation,electrocardiography (ECG),embryonic stem cell derived cardiomyocytes,Long QT syndrome (LQTS)

## Acknowledgement

This study was supported by TÜBITAK under 1003 Scientific and Technological Research Projects Funding Program by project no: 213S194.

# Abstract: The clinical significance of the increased expression levels of *BID* gene in Larynx Cancer

**Authors:** Semra DEMOKAN, Sena SEN, Onder ERYILMAZ, Can DORUK, Gizem ESENTURK, Murat ULUSAN

**Affiliations:** Istanbul University, Istanbul, Turkey

## Background/aim

Apoptosis is a programmed cell death, a physiological event that regulates the homeostasis of the organism by the destruction of unnecessary/damaged cells without an inflammatory response. The *BID* (*BH3 Interacting Domain Death Agonist*) protein, encoded by the *BID* gene, is a member of the BCL2 family that regulates apoptosis by activating the mitochondrial death pathway via Caspase 8, allowing cytochrome C to be released. In the literature, increased expression levels of *BID* gene have been observed in glioma, lymphoma, colon, prostate and cervical cancers; while in Hepatitis B Virus (HBV) infected hepatocellular carcinoma, its decreased expression levels have been reported. In our study (I.U.BAP-ONAP-42152), we aimed to investigate the differentially expressed levels of *BID* gene in Turkish larynx cancer patients and to reveal the clinical significance.

## Materials and methods

The expression status of *BID* was analyzed in tumor and matched-normal tissue samples of 50 larynx cancer patients using LightCycler 480 by the quantitative real-time polymerase chain reaction method. The results were compared with clinicopathological data.

## Results

*BID* and the reference gene expression status were analyzed by calculating the threshold cycle numbers (Ct) as fold changes using the 2^-ΔΔCt^ method. After evaluation of the expression levels, we selected the ratio of >=2 as the threshold for the differentially expressed *BID*. The increased expression levels of *BID* were observed in 46% (23/50) of tumor samples compared with matched normal tissue, whereas the expression levels were decreased in 28% (14/50) of patients. The statistical significanceswere observed between the increased expression levels of *BID*with recurrence (p=0.024) and anatomical region (p=0.018).

## Conclusion

The increase in the expression level of the *BID* gene is thought to be associated with the development and recurrence of larynx cancer.

## Keywords

Larynx cancer,expression,*BID* gene

# Abstract: Contact with senescent mesenchymal stem cells (MSCs) modulate secretome composition of myeloma cells

**Authors:** Serife AYAZ-GUNER, Mustafa Burak ACAR, Eda MERT GÖKDUMAN, Nicola ALESSIO, Umberto Galderisi, Servet OZCAN

**Affiliations:** 1 Abdullah Gül University, Kayseri, Turkey; 2 Erciyes University, Kayseri, Turkey; 3 University of Campania "Luigi Vanvitelli", Naples, Italy

## Background/aim

Aging is one the largest risk factor for the onset of cancer which could be to certain extent a consequence of the increased senescent cells during aging. Senescent mesenchymal stem cells (MSCs) secrete signals known as "senescence-associated secretory phenotype" which could modify their microenvironment and contribute to cellular proliferative arrest. We previously showed that once the senescent MSCs contacted with myeloma cells, myeloma cells can manipulate senescent MSCs secretome and impair their anti-proliferative activity to survive and grow. Cancer cells might secrete molecules that manipulate the secretome content of MSCs. In order to elucidate this interaction, we aimed to investigate the secretome of myeloma cells.

## Materials and methods

ARH-77 myeloma cells primed with MSCs by direct contact and indirect contact by using thincert. Following the priming step, MSC cells removed and the secretome of ARH77 collected by StrataClean resin. In gel digested proteins were analyzed by using LC MS/MS.

## Results

The composition of ARH77 cell's secretome was profoundly modified by the direct interaction with senescent MSCs. Globally, direct priming induced the production of 129 proteins and repressed the secretion of 67 others. However indirect priming had very little effect on cancer cell secretome.

## Conclusion

This result suggest that cancer cells respond to direct contact with senescent mesenchymal stem cells more efficiently. Further investigation on pathways and signalling molecules needs to be done.

## Keywords

Molecular biology,proteomics,cancer cells

## Acknowledgement

This research partially supported by the Scientific and Technological Research Council of Turkey (TÜBITAK project no. 114S873) and the Erciyes University Research Project Fund (project no. BAPFYL-2015-5616).

# Abstract: A candidate gene obtained from methylation/expression arrays showed methylation- independent expression loss in oral carcinoma

**Authors:** Semra DEMOKAN, Onder ERYILMAZ, Sevde COMERT, Sena SEN, Murat ULUSAN

**Affiliations:** Istanbul University, Istanbul, Turkey

## Background/aim

In our study (supported by TUBITAK-SBAG-114S497), we aimed to investigate the potential epigenetic biomarker candidate genesobserved methylation-dependent expression loss via methylation and expression array methods in oral squamous cell carcinoma (OSCC) patients.

## Materials and methods

After synthesis of cDNA/BC-DNA from RNA/DNA samples isolated from 9 OSCC patients' tumor and matched-normal tissues, the methylation profile was analyzed by Infinium-II using "Illumina Human Methylation 450 chips". The expression profile was analyzed by the Illumina Genome Studio using IlluminaiScan. The expression and methylation status of the candidate genes, were validated in tissues (tumor and matched-normal) of 20 OSCC patients using LightCycler480 by QRT-PCR/QMSP methods, respectively.

## Results

After bioinformatic analysis, we identified 409 candidate genes using specific probes detecting methylated the CpG regions in the tumor groups compared to matched-normal groups, while the decreased expression levels in tumors were observed in 35 genes. From those data set, we were searching epigenetic candidate biomarker genes observedmethylation-dependent expression loss. A candidate gene from this gene panel showed significant hypermethylation and decreased expression levels, and it was choosing to perform further validation in a larger patient cohort. The decreased expression levels of this candidate gene (unpublished data) that play an important role in the cell adhesion processes, was observed in 50% (10/20) of tumor samples compared with matched normal tissue, whereas the expression levels were increased in 15% (3/20) of the patients. Methylation was not observed in all tumor groups.

## Conclusion

We concluded that hypermethylation may not the suppression mechanism for this gene's decreased expression. The methylation results of the array cohort and the validation cohort was different since the patients were not the same in both groups. The decreased expression levels of this candidate gene may be associated with the carcinogenesis mechanism of oral cavity.

## Keywords

Oral Squamous Cell Carcinoma,Methylation,Expression,Biomarker

# Abstract: Identifying miRNA-associated candidate molecular pathways and key regulators in schizophrenia pathogenesis by using bioinformatic analyses

**Authors:** Dilek PIRIM

**Affiliations:** Bursa Uludag University, Bursa, Turkey

## Background/aim

Accumulating evidence indicates the value of utilizing miRNAs as a biomarker for Schizophrenia (SCZ), however their potential functions in the SCZ pathogenesis are largely unknown. The aim of this study to identify putative functions of miRNAs in molecular networks and assess their interactions with key regulators contributing to the SCZ-associated pathways by using bioinformatic tools.

## Materials and methods

Microarray data set (GSE54578) was downloaded from Gene Expression Omnibus. Differentially expressed miRNAs (DEMs) were analyzed in GEO2R using Limma R and the cut-off criteria for DEMs were set as an adjusted-p < 0.05 and a fold change>1.5 or < -1.5. DIANA miRPath and DAVID databases were used for functional enrichments of the DEMs and their targets, respectively. Target genes of the miRNAs were analyzed by using Ingenuity Pathway Analysis and constructed protein-protein interaction was imported to Cytoscape for visualization and hub gene identification. Transcription factor (TF)-miRNA interactions were also assessed by Transmir and expressions of the possible hub genes were evaluated in SCZ patients and healthy individuals through schizophrenia database (SZDB).

## Results

A total of 18 DEMs were found in SCZ patients as compared to controls. Top Kyoto Encyclopedia of Genes Genomes (KEGG) category was related immune system (chemokine signaling pathway). Functional enrichment of significant DEMs revelaed several significant pathways highlighting their roles in glycan metabolism, substance addiction and cancer. Seven hub genes were identified by Cytohubba and evaluating them in SZDB revealed *AKT1* and *PI3K* were dysregulated in SCZ. *TP53*, one of the hub gene, was the top transcription factor that was predicted to be regulated by 10 miRNAs in TF-miRNA interaction analyses.

## Conclusion

Our results highlight candidate molecular pathways and key regulators that may involve in the etiology and pathogenesis of SCZ of which can be utilized to further elucidate the SCZ susceptibility and management.

## Keywords

Schizophrenia,miRNA,bioinformatic

# Abstract: A novel in vitro screening method unravels targets for NK cell-based cancer immunotherapy

**Authors:** Didem OZKAZANC UNSAL, Ece Canan SAYITOGLU, Benjamin John JOSEY, Michelle HARTMAN, Elif CELIK, Michael CHROBOK, Anna-Maria GEORGOUDAKI, Harry Thomas TEMPLE, Batu ERMAN, Evren ALICI, Adil Doganay DURU, Tolga SUTLU

**Affiliations:** 1 Sabanci University, Istanbul, Turkey; 2 Nova Southeastern University, Davie, United States; 3 Karolinska Institutet, Stockholm, Sweden

## Background/aim

Natural killer (NK) cells of the innate immune system are recognized for their ability to distinguish and potently kill tumor cells. NK cell-mediated lysis is maintained by a balance between several activating and inhibitory receptors that either trigger or stop NK cell effector functions upon engaging their ligands. In this study, we aim to dissect this complex balance into single receptors to develop an in vitro cell-based screening tool against tumor cells that can rapidly identify receptor specific anti-tumor responses.

## Materials and methods

Genes coding for 20 different NK cell surface receptors were cloned into lentiviral vectors for genetic modification of NK-92 cell line. Genetically modified (GM) NK-92 cells were enriched using flow cytometry-based cell sorting and overexpression of each receptor was confirmed by flow cytometry. We analyzed degranulation capacities of GM NK-92 cells against various well-characterized human cancer cell lines including carcinoma, sarcoma, melanoma and leukemia.

## Results

Lentiviral genetic modifications did not hamper cytotoxic capacity of GM NK-92 cells; rather induced elevated tumor cell line killing by receptors such as DNAM-1 and NKG2D compared to controls. We further evaluated the functional response of these receptors in triggering degranulation against primary human sarcoma explants. We observed that DNAM-1 overexpressing GM NK-92 cells elicited increased anti-tumor responses. We confirmed that this response was indeed DNAM-1- dependent by interfering with CD155/DNAM-1 interaction using blocking antibodies targeting either the receptor or its ligand.

## Conclusion

Our results showed the feasibility of an in vitro genetic screening approach to identify response-triggering receptors in genetically modified NK-92 cells. This tool has the potential to rapidly identify patient-specific targets for adoptive immunotherapy of cancer.

## Keywords

Immunology,Natural killer cells,Cancer immunotherapy

## Acknowledgement

This research is supported by Scientific and Technological Research Council of Turkey (TUBITAK) grant 114Z861 and BIDEB 2214-A Scholarship.

# Abstract: Development of next generation vaccine technology based on carrier/adjuvant properties of ASC specks

**Authors:** Aylin ALKAN, Mesut BERBER, Davod KHALAFKHANY, Nesrin ÖZÖREN

**Affiliations:** Bogaziçi University, Istanbul, Turkey

## Background/aim

Pathogen-associated molecular pattern (PAMPs) and danger-associated molecular patterns (DAMPs) are sensed by the nucleotide binding oligomerization domain like receptor (NLR) family of proteins in cytosol for the regulation of innate immune response. Certain NLRs, such as NLRP3, induce formation of inflammasome complexes. Activation of inflammatory Caspases usually occurs via interaction with apoptosis-associated speck-like protein (ASC). The adaptor protein ASC contains a C-terminal caspase recruitment domain (CARD) and an N-terminal pyrin (PYD) domain. Upon stimulation, ASC combine into large filamentous structures which eventually came together forming a globular structure also known as the ASC-speck. These specks might remain inside the cells or can be released into extracellular matrix and amplify the inflammatory response. Our group previously showed that ASC specks can artificially be loaded with antigens and these, antigen loaded particles can remain stable at 37C at least for a month. Also, in In vivo model after intra-peritoneal injection ASC specks tend to accumulate in the spleen with relatively long-lasting stability.

## Materials and methods

We purified the ASC specks particles carrying antigenic epitopes and evaluated their properties as novel adjuvant/carrier to achieve immunity in C57/BL6 mice as model for tumor eradication or immunity against viral infections.

## Results

After stimulation of macrophages driven from THP-1 cells we showed an increase in secretion of certain inflammatory cytokines including IL-1 beta and TNF alpha. Furthermore, C57BL/6 mice vaccinated with tH5-ASC specks showed higher IgG level in comparison to those receiving to those vaccinated by alum adjuvanted or non-adjuvant antigen. Lastly, we showed that animals immunized via I.P injection of tOVA-ASC specks are capable of eradication or growth suppression of EG7-OVA thymoma tumors expressing chicken ovalbumin

## Conclusion

Antigen loaded ASC specks showed a superior immune induction capability in comparison to antigen plus Alum or antigen alone.

## Keywords

Adjuvant,Vaccine,Immune therapy,ASC-speck

# Abstract: Progenitor cells differ in the ratio of cellular structures in young and old brains as revealed in zebrafish.

**Authors:** Melis TEMEL, Begün ERBABA, Ozge KOÇKARA, Figen KAYMAZ, Michelle ADAMS, Ayça ARSLAN ERGÜL

**Affiliations:** 1 Hacettepe University, Ankara, Turkey; 2 Bilkent University, Ankara, Turkey

## Background/aim

Brain aging is inevitable for almost all organisms, however, the mechanisms are yet to be studied. Zebrafish is an excellent model to investigate aging. In this study, we examined the cellular compartments of brain progenitor cells and differentiated in young and old zebrafish.

## Materials and methods

We isolated progenitor cells from young (10 months old) and old (20 months old) zebrafish brains and maintained in cell culture. Then we examined the cells in transmisson electron microscope. We counted the cellular compartments and properties manually and normalized to number of cells counted.

## Results

We found that young (Y) and old (O) cells have similar numbers of lisosomes (3.4 (Y) vs 3 (O) per cells counted) and mitochondria (13.5 (Y) vs 13.8 (O) per cells counted). In young and old specimens, there were similar ratios of macrophages (8% (Y) vs 12% (O)), apoptotic cells (2% (Y) vs 2% (O)), and enlarged nuclei (46% (Y) vs 41% (O)). However, in terms of necrotic cells, heterochromatin formation, and number of vacuoles, young and old samples differed. There were 26% and 16% necrotic cells, 31% and 18% heterochromatin foci, and 21% and 31% vacuoles, respectively in young and old samples

## Conclusion

In this preliminary work, we wanted to identify the structural differences in brain progenitor cells. We found that the young cells were enriched in necrotic cells and heterochromatic foci, whereas the old cells were enriched in vacuoles. We will repeat the experiments to validate our results.

## Keywords

brain progenitor cells,zebrafish,cell structure

## Acknowledgement

This work has been funded by TUBITAK grant 114S548.

# Abstract: Role of tissue transglutaminase transamidation and GTP binding activity on renal cell carcinoma drug response

**Authors:** Burge ULUKAN, Ayca Ece NEZIR, Ajna BIHORAC, Halime ILHAN, Robert KIRALY, Laszlo FESUS, Dilek TELCI

**Affiliations:** 1 Yeditepe University, Istanbul, Turkey; 2 University of Debrecen, Debrecen, Hungary

## Background/aim

Renal cell carcinoma (RCC), the most common form of kidney cancer, has been stated as the 6th severe cause of cancer-related death. The kinase inhibitors Sorafenib and Everolimus are the first line treatments for RCC. However, the effectiveness of anticancer agents in clinical practice needs to be improved to achieve a complete response. Tissue transglutaminase (TG2) is a multifunctional protein that is strongly associated with cancer progression and metastasis. This impact of TG2 was partly attributed to its involvement in drug resistance and cell survival pathways, which may hinder the effects of chemotherapy. The objective of this study was to understand whether the transamidation or GTP-binding/GTPase activity of TG2 is responsible for the drug response observed in RCC.

## Materials and methods

RenCa mouse RCC cells were transduced with lentiviral particles encoding wild-type TG2, transaminase-deficient C277S and W241Aand the GTP-binding deficient R580ATG2 mutants. To evaluate the drug response phenotypes, cell proliferation assay and Annexin V staining were performed following drug treatments. The interaction between TG2 and PDGFR proteins was detected by co-immunoprecipitation.

## Results

Our results showed that non-transduced, wild-type TG2, TG2-C277S and TG2-R580A mutant RenCa cells demonstrated a similar cell viability pattern against Everolimus and Sorafenib. On the other hand, 2 to 3-fold increase in the cell survival was detected for the TG2-W241A cells after drug treatments. In consistency, Co-IP results demonstrated that the physical association of TG2 and PDGFR was significantly increased in these cells.

## Conclusion

Our data support the notion that GTP-binding activity of TG2 is closely related with the development of drug resistance in RenCa cells, and the increase in survival capacity may be due to the activation of PDGFR-dependent survival signaling. Thus, inhibitors targeting the GTP-binding activity of TG2 may increase the efficacy of anticancer treatments in RCC.

## Keywords

Renal cell carcinoma,tissue transglutaminase,drug resistance

# Abstract: The role of ERBIN in breast cancer and drug resistance

**Authors:** Gizem SUNAR, Özge SAATÇI, Isik YULUG

**Affiliations:** Bilkent University, Ankara, Turkey

## Background/aim

Erbin is an Erbb2 interacting protein with roles in many signaling pathways. Dysregulation of Erbin can lead to tumorigenesis. Breast cancer is one of the types of cancer that is affected by Erbin regulation. However, it is unclear how Erbin regulates the biological behavior and drug resistance of breast cancer cells. The main aim of our study is to explore the role of the Erbin gene in drug resistance in breast cancer.

## Materials and methods

The gene expression level of Erbin was first analyzed by using seven different NCBI GEO Datasets. The Erbin protein level in native breast cancer cells and their drug resistant lines was then demonstrated with Immunoblotting.

## Results

Bioinformatic analysis of GEO datasets has shown that ERBIN mRNA expression is lower in basal tumors than non-basal tumors and in high-grade tumors than low grade tumors in five different datasets. High level of ERBIN expression predicts better survival in breast cancer patients treated with chemotherapy (especially in TNBC patients) or targeted therapies while the Erbin level does not change the survival rates of untreated breast cancer patients. We hypothesize that the Erbin expression level can alter the effect of the drug treatment. Moreover, immunoblot results demonstrate that the drug resistant cell lines display lower levels of Erbin protein compared to non-resistant cell line counterparts.

## Conclusion

The ERBIN gene may play an important role in drug resistance of breast cancer cells.

## Keywords

ERBIN,breast cancer,drug resistance

## Acknowledgement

We would like to thank Özgür Sahin's Lab for giving us drug resistant cell lines and Özge Saatçfrom the same lab for her NCBI bioinformatics analysis of GSE datasets.

# Abstract: Real-time and label-free evaluation of cell - surface interactions

**Authors:** Abdulhalim KILIÇ, Fatma Nese KÖK

**Affiliations:** Istanbul Technical University, Istanbul, Turkey

## Background/aim

Understanding of interfacial phenomena between cell and biomaterial helps to control the integration of biomaterials in the body. In this study, the adhesion process of human fetal osteoblastic bone cells (hfOB) on different surfaces was investigated taking the advantage of quartz crystal microbalance with dissipation (QCM-D), which can monitor the interfacial phenomena between biomaterials and cells in real time, label-free and quantitative manner.

## Materials and methods

A defined number of cells were passed within the sensor chamber. After the surface was saturated, the pump was stopped (1 hour), and adhesion and later spreading was monitored for 17 h on hydroxyapatite, silk fibroin, gelatin coated sensor surfaces and gold surfaces as the reference substrate.

## Results

Passive flattening of the cells was tracked from the initial increase in mass (~frequency shift; Δf) and viscoelastic properties (~dissipation shift; ΔD). Δf and ΔD reached a constant value at the end of 1 h flow showing that the cell sedimentation was completed. The initial cell attachment was similar and sustained mostly by nonspecific interactions. Then, increase in signals continued as a result of cell spreading for several hours even the cell seeding was stopped. At the end of 18 h, Δf changes and the acoustic ratio (|ΔD/Δf|), which is an indication of protein-mediated specific interactions and focal adhesion maturation, were observed and compared.

## Conclusion

Interaction of mammalian cells with different surfaces was quantitatively determined with QCM-D. This method offers label-free detection of interfacial phenomena on cell - biomaterial surface so important in interpreting biomaterial behaviour in contact with tissue.

## Keywords

Quartz crystal microbalance,Human fetal osteoblast cells,bio-nano interfaces

# Abstract: The mutation spectrum of our patients with nephrological disorders

**Authors:** Hamza POLAT, Ceren ALAVANDA, Özlem YILDIRIM, Ebru ARSLAN, Ipek YILMAZ, Nurdan Yildiz, Pinar ATA, Harika ALPAY

**Affiliations:** Marmara University, School of Medicine, Istanbul, Turkey

## Background/aim

Nephrological disorders have genetic heterogeneity and although the symptoms are usually proteinuria and hematuria, the genetic etiology might be arising from different mutations. Herein we aimed to discuss our patients' findings and genetic results for this heterogeneous group.

## Materials and methods

Patients admitted to our outpatient clinic from June 2018 to September 2019 with a symptom scale of hematuria, proteinuria, abdominal pain, renal cysts, nephrolithiasis. There were 15 females and 10 males aged between 6 months to 35 years of age. The urinary system ultrasonography, urine analysis and biochemical examinations were evaluated for all of them. For genetic analysis after peripheral blood DNA isolation and adapter application, we had amplified 33 genes associated with nephrological disorders. After sequencing at Illumina Miseq^R^ platform the vcf files were analysed at Sophia DDM v.4 software. The mutations were analysed with uptodate databases for prediction of pathogenicity and classified according to ACMG guidelines. Segregation analysis and genetic counselling were performed with Sanger Sequencing for all families.

## Results

We have detected 25 mutations associated with disease phenotype. There were three patients with pathological mutation at more than one genes and one patient had two homozygous mutations at the same gene that one of them is pathological while the other was defined as variant of unknown significance (VUS). We had 3 Bartter Syndrome, 5 Polycystic Kidney Disease, 5 renal tubular acidosis, 5 Alport Syndrome, 1 Hyperoxaluria, 1 Recurrent Nephrolithiasis, 1 Hypophosphatemia, 1 Renal Agenesis, 2 Nephrotic Syndrome and 1 Wilm's Tumour patients whose diagnosis were confirmed genetically.

## Conclusion

Kidney disorders have diverse symptoms and genetically heterogenous. For the clinical diagnosis at this century NGS and Sanger Sequencing confirmation analysis are important tools. Yet there is need of functional studies to be done.

## Keywords

Nephrological disorder,Pathogenic Mutation,Next generation sequencing

# Abstract: The effect of signal peptides in recombinant phytase production

**Authors:** Kaya ISLEYEN, Filiz ERÇELIK, Emrah NIKEREL, Bahar SOGUTMAZ ÖZDEMIR

**Affiliations:** Yeditepe University, Istanbul, Turkey

## Background/aim

Enzymes are important macromolecules relevant for industrial production. High level expression of these enzymes is key for large-scale production. Recombinant DNA technology is used to produce tailor-made enzymes with increased expression level and yield. Signal peptides (SP) are short sequences that guide the produced enzyme to its final destination. Finding the suitable SP for the enzyme of interest directly affects the amount of secretion. However, an optimal SP for a protein can be predicted only upon an intensive screening. Phytase is a significant commercial enzyme that hydrolyze phytic acid to inorganic phosphate. *Bacillus subtilis* is able to express proteins extracellularly, therefore is a good candidate for SP optimization studies. The aim of the study is to produce extracellular phytase from *B. subtilis* RIK 1285 with signal peptide modifications.

## Materials and methods

For cloning of the phytase gene, *B. subtilis* 168 was used as source and *B. subtilis* RIK1285, a protease deficient strain, was the expression host. A plasmid library was prepared with 173 signal peptides belonging to the *B. subtilis* Sec pathway and transformants were screened for their phytase activity. The selected recombinant plasmids were sequenced to determine the inserted SP.

## Results

A vector expressing phytase was successfully constructed and cloned into the host organism, *B. subtilis* RIK1285. In total, twenty-nine *B. subtilis* transformants were obtained with varying phytase activity and sequencing of the selected recombinant plasmids revealed that the colonies contained the signal peptides, lipB or dltD.

## Conclusion

This study underlines the importance of the modifications on a molecular level for increasing the efficiency of recombinant enzyme production.

## Keywords

Recombinant enzyme,phytase,signal peptides,*Bacillus subtilis*

# Abstract: Motif discovery for engineering transcription factor binding sites in *Aspergillus niger*

**Authors:** Filiz ERÇELIK, Emrah NIKEREL

**Affiliations:** Yeditepe University, Istanbul, Turkey

## Background/aim

Enzymes have great importance in industrial biotechnology context owing to their wide application in different industries such as food, feed, detergent etc. High level production of enzymes depends on numerous parameters such as selecting a combination of suitable host, environmental conditions, and expression system (in case of heterologous production). Promoter regions located upstream of gene of interest contain several key regulatory components, such as transcription factor binding sites (TFBS), where Transcription Factors (TFs) bind to. TFs also contain various domains enabling binding of RNA polymerase and other regulatory components (e.g. enhancers and silencers), thus the system (tightly) regulates the transcription of the gene of interest. As a whole, engineering of TFBS and other components attracts increasing attention especially within synthetic biology context, as their optimization significantly improves the enzyme production. In this study, TFBS in promoters located upstream of differentially expressed genes (DEGs) in Aspergillus niger were scanned and several TFBS motifs were discovered to be further used for promoter tuning.

## Materials and methods

Various *A. niger* transcriptomics data were analyzed to find out DEGs include transporters, proteases, and other cellular proteins. 1000 bp upstream sequences of these genes were chosen as possible promoter regions and analyzed using MEME Suite (http://meme-suite. org/tools/meme) tool for discovery of TFBS motifs. Each dataset and the corresponding genes were analyzed separately and the most common TFBS motif was found. Finally, they were aligned to check for similarities in their sequences.

## Results

A collection TFBS motifs was obtained including common sequences such as CT-rich regions, key sequences for mRNA stability.

## Conclusion

This approach constitutes the basis of engineering of regulatory elements in promoter regions. Output of this research provides key information for further promoter tuning studies.

## Keywords

*Aspergillus niger*,enzyme production,industrial biotechnology,transcription factors

## Acknowledgement

Financial support for Filiz Erçelik via TÜBITAK 2211-C Program is gratefully acknowledged.

# Abstract: Characterization of C-terminal lysine heterogeneity in biosimilar antibodies

**Authors:** Melike BERKSÖZ, Pegah ZAHEDIMARAM, Lolai IKROMZODA, Basak ÖZATA, Huriye ERDOGAN, Yilmaz ÇAPAN, Batu ERMAN, Tolga SUTLU

**Affiliations:** 1 Sabanci University, Istanbul, Turkey; 2 ILKO ARGEM Biotechnology Center, Teknopark, Istanbul, Turkey; 3 Department of Chemistry, Gebze Technical University, Istanbul, Turkey

## Background/aim

Structural heterogenities observed in therapeutic monoclonal antibodies (Mab) may impact the stability, efficacy and safety of the drug product. C-terminal lysine processing is a common post-translational modification in recombinant production of antibodies. Carboxypeptidases present in the bioreactor mixture were found to directly affect the C-terminal lysine variants. This study aims to identify C-terminal modifications in a biosimilar Mab by using Cation Exchange (CEX) Chromatography and Liquid Chromatography-Mass Spectrometry (LC-MS).

## Materials and methods

Presence of positively charged C-terminal lysine residues increases the pI of the molecule and allows for a charge-based separation of two, one or no lysine bearing variants. Intact mass profiles were determined with UPLC- Xevo G2 XS- QTOF system using a C_4_ reverse-phase column. Unprocessed C-terminal lysines cause a 128 Da upwards shift in the mass spectrum which allows for a straightforward mass-based separation. Presence of C-terminal lysines was confirmed by treating the samples with Recombinant Carboxypeptidase B which catalyzes the hydrolyis of basic amino acids at the carboxy terminus. Charge variants and mass profiles before and after the enzymatic reaction were compared.

## Results

A significant level of basic variants observed in CEX chromatogram were removed after the carboxypeptidase treatment. Intact mass profiles displayed three distinct species, each with a mass shift of nearly 128 Da.

## Conclusion

CEX chromatography and LC-MS experiments indicated that the charge heterogeneity in this biosimilar candidate is largely caused by incomplete removal of C-terminal lysines in the bioreactor.

## Keywords

C-terminal lysines modifications,Biosimilar Mabs,Cation Exchange Chromatography,Mass Spectrometry

## Acknowledgement

This project is supported by Tubitak Kamu Arastirmalari Destek Grubu (KAMAG)

# Abstract: Effects of *Helichrysum arenarium* (Immortelle) on ECV 304 and Ishikawa cell lines

**Authors:** Rumeysa CEBECIOGLU, Dilan AKAGUNDUZ, Ilknur KORKMAZ, Asena ARICIOGLU, Aykut KUL, Tunc CATAL

**Affiliations:** 1 Uskudar University, Istanbul, Turkey; 2 Istanbul University, Istanbul, Turkey

## Background/aim

*Helichrysum arenarium* (Immortelle) plant contains pharmacologically important substances such as flavonoids, catechins and proanthocyanidine. The plant has antioxidant features acting as immune system stimulant, anticancer, anticoagulant, antiallergic and antimicrobial agent. In this study, the effects of *Helichrysum arenarium* plant on ECV 304 (human endothelial cell line) and Ishikawa cells (human endometrial adenocarcinoma cells) were investigated.

## Materials and methods

The effects of dimethylsulfoxide (DMSO) and methanol extracts obtained from *Helichrysum arenarium* plant on ECV 304 and Ishikawa was investigated using MTT Assay. Malondialdehyde (MDA) levels were measured, biochemically. Fluorescence microscopy was ap- plied for the examination of cells using ethidium bromide/acridine orange staining technique. Es- sential oils of *Helichrysum arenarium* extracts were analysed using gas chromatography mass spec- trometry.

## Results

DMSO extract of *Helichrysum arenarium* decreased cell viability down to 40% in Ishikawa cells while cell viability of ECV304 remained around 60% at the concentration level of 500 g/mL. MDA levels were increased in Ishikawa cells in the same concentration. 2-Palmitoylglycerol and palmitic acids were found as the major essential oils in DMSO extracts of *Helichrysum arenarium*.

## Conclusion

In conclusion, *Helichrysum arenarium* may show therapeutic effect against Ishikawa cells.

## Keywords

Cytotoxicity,*Helichrysum arenarium*,lipid peroxidation,oxidative stress

# Abstract: Runt-related transcription factor 2 (Runx2) affects the expression of some extracellular matrix proteins

**Authors:** Sevgi BINAY, Engin KAPTAN

**Affiliations:** Istanbul University, Istanbul, Turkey

## Background/aim

RUNX2 is one of the runt related transcription factor that are important regulatory molecules of osteoblast/ chondrocyte differentiation. It has recently been discovered that RUNX2 is overexpressed in human cancers and is one of the major precursors of bone metastasis. The cancer cell metastasis requires modifications of extracellular matrix (ECM) and reduction in ECM-cell inter- action. This process is performed by various enzymes and proteins secreted by cancer cells. The aim of this study was to investigate the effect of RUNX2 transcription factor on expression of proteins such as OPN, SPARC, LOX and HPA1 which have important roles in ECM modification and ECM- cell interaction in human breast cancer cell lines.

## Materials and methods

For this purpose, RUNX2 *knockdown* model was created by using RUNX2 siRNA in human breast cancer oestrogen receptor negative MDA-MB-231 and positive MCF-7 cell lines. The changes in the mRNA and protein expression levels of ECM proteins in this model were shown by the PCR and Western blotting methods respectively.

## Results

The data obtained from this study showed that there was a decrease in both mRNA and pro- tein levels of HPA1, SPARC and LOX in MCF-7 cells, but there was no change in OPN mRNA and protein levels. However, the protein and mRNA levels of HPA and LOX were significantly reduced in MDA-MB-231 cells and SPARC expression was significantly increased.

## Conclusion

Consequently, the regulation of matricellular proteins, which are HPA1, LOX and SPARC, expressions through RUNX2 appears to be one of the regulatory mechanisms of the malig- nant potential of human breast cancer cells. Therefore, RUNX2 can be considered as a target mol- ecule in the treatment process of breast cancer.

## Keywords

Breast cancer,HPA1,LOX,OPN,RUNX2,SPARC

## Acknowledgement

This study was supported by Scientific Research Projects Coordination Unit of Istanbul University with the project number 25103.

# Abstract: Evaluation of *ALX* homeobox gene variants using in silico tools

**Authors:** Melih ÖZTEPE, Meliha KOÇ, Elif Beyza KARTALOGLU, Elif UZ YILDIRIM

**Affiliations:** Bursa Uludag University, Bursa, Turkey

## Background/aim

Aristaless-like homeobox (*ALX*) genes belong to homeobox genes and there are 3 paralogs of this protein family in humans. Mutations in these genes usually result in frontonasal defects in humans. We aimed to evaluate the functional effects of SNVs using different in silico tools and to summarize the allele frequencies.

## Materials and methods

Only missense, nonsense, and frameshift SNVs were filtered using Variation Viewer website. The molecular consequence of each variant was evaluated using three different in silico tools them (SIFT, PolyPhen2 and Mutation Taster) and combined score was determined. The allele frequencies of the variants were obtained using gnomAD. RegulomeDB web tool was used to interpret the regulatory important points. Posttranslational modification status of the proteins was evaluated using Phosphosite Plus web tool.

## Results

Total number of variants (combined score of pathogenic variants) for the *ALX*1, *ALX*3, and *ALX*4 genes were 265 (28%), 278 (33%), and 381(38%), respectively. The highest number of pathogenic variants were located in exon 2 of the *ALX*3 gene and in exon 4 of the *ALX*1 and *ALX*4 genes. The data obtained from the gnomAD indicated that 20% variants in the *ALX*1, and 42% variants in the *ALX*4 have allele frequency of >0.01. Interestingly, none of the variants on *ALX*3 gene have allele frequency of >0.01. According to the RegulomeDB analysis scores, only one *ALX*4 exonic variant had a score less than 2, which means only this exonic variant has an effect on the regulation of the *ALX*4 gene. From different types of posttranslational modifications, only phosphorylation of *ALX* proteins is affected in all 3 paralogs.

## Conclusion

The variant analysis of the 3 paralog genes gives an idea about how the number and functional consequences of these variants and their impact on phenotype could be variable.

## Keywords

*ALX* genes,SNV,in silico analysis

# Abstract: Investigation of the Role of M3 muscarinic receptor in Chronic Myeloid Leukemic Cells

**Authors:** Zehra KANLI, Banu AYDIN, Hülya CABADAK

**Affiliations:** Marmara University, Istanbul, Turkey

## Background/aim

K562 cells are used to as model systems for normal myeloid, development of myeloid leukemia, control of differentiation studies. K562 cell line represent early differentiation stage in granulocyte lineage. Non-neuronal acetylcholine has a important role in migration, proliferation and differentiation of different types of cell. It was recently suggested that the non-neuronal cholinergic system has also a important role in different types of cancer cells. Muscarinic receptors are part of G protein coupled receptors . K562 cells express M_2_, M_3_ and M_4_ muscarinic receptors. Especially, muscarinic type 3 receptor (M_3_R) plays an important role in different cancer cells. Bax and Bcl-2 proteins are related to apoptosis. Aim of this study firstly was to examine effects of pilocarpine, TNFalpha and other drugs on apoptosis. Secondly, we aimed to demonstrate the mitogenic effects of FBS, TNF alpha and EGF in K562 cells proliferation. Effects of Pilocarpin on The AChE activity has been detected.

## Materials and methods

Cell viability were evaluated by the 0.4% trypan blue exclusion test by using a hemocytometer. Proliferation assays were performed by measuring BrdU incorporation during DNA synthesis in proliferating cells. Effects of TNF alpha, EGF, M_3_ agonist and antagonist on bcl and bax expression were detected by western blotting method. AChE level was detected by the QuantiChrom Acetylcholinesterase Assay Kit (DACE-100).

## Results

This study proposes that pilocarpine mediated M3R activation can promote apoptosis and inhibit cell proliferation.

## Conclusion

We suggest that 1m Pilocarpine and Egf inhibitor may have a significant effect on apoptosis in K562 cells.

## Keywords

Cholinergic receptors,pilocarpine,K562 cells,apoptosis

## Acknowledgement

This research was supported by Marmara University Research Fund (the project numbers: SAG-C-YLP-080415-0101)

# Abstract: Information theory to explain allometric relation among genes' and proteins' lengths

**Authors:** Yekbun ADIGUZEL

**Affiliations:** Altinbas University, Istanbul, Turkey

## Background/aim

The aim of this study is by using the information theory, trying to explain the allometric relation which can be defined as a scaling relation with power, and can be observed between the lengths of proteins and the DNA that encode them, in terms of their number of residues.

## Materials and methods

Our ongoing studies on the explanation of information amounts between the proteins and encoding DNA with a modified calculation method are present. Accordingly, the information amount discrepancies that is resulting from the calculation with information theory was tried to be eliminated by modification of the calculation, with the assumption that there is no loss but instead transformation of information, during information transfer from DNA to the protein. Following this, relation between the lengths of these macromolecules that is observed through the present approach was compared with the bioinformatics analysis data that can reveal allometric relation.

## Results

A model function, slope of the trendline function of which contains a constant sum (or multiplier) difference but is significantly close to that of maximum length findings of the bioinformatics analysis of experimental data was observed and termed as the best model.

## Conclusion

Constant sum (or multiplier) difference between the functions of the best model and the trendline of the bioinformatics analysis' findings implies that our model requires improvement, when the assumption of the current study is deemed as correct. Our next issue is how to interpret the modification in the theory within the context of allometric relation.

## Keywords

Information communication theory,gene length,protein length,allometric scaling

# Abstract: Quercetin-induced heat shock proteins inhibition and cell death in human breast cancer cells

**Authors:** Ezgi KIYGA, Aslihan SENGELEN, Zelal ADIGÜZEL, Evren ÖNAY UÇAR

**Affiliations:** 1 Istanbul University, Istanbul, Turkey; 2 TUBITAK Marmara Research Center, Kocaeli, Turkey

## Background/aim

Breast cancer is the most commonly diagnosed cancer types in women. High levels of heat shock proteins (Hsp, especially Hsp27, Hsp70 and Hsp90) are associated with poor prognosis. Due to the inadequacy of current treatments and side effects, quercetin (Qu), which is an Hsp inhibitor as a natural compound, can be a good alternative. However, studies on the effect of Qu on Hsps in breast cancer are insufficient. Herein, we aimed to examine the Qu-induced Hsp inhibition and apoptosis in human breast cancer cells MCF-7 (positive for estrogen receptor, ER+) and MDA- MB-231 (ER-).

## Materials and methods

The effect of quercetin on cell viability was determined by MTT assay. DNA damage was detected by Comet assay. Caspase activity was determined by colorimetric activity assay. Western blot analysis was performed to examine the effects of Qu on Hsp27, Hsp70, Hsp90, caspase-3 and PARP-1.

## Results

We found that Qu blocks cell proliferation in a dose- and time-dependent manner. Qu treat- ments (10, 25 and 100 μM) resulted in down-regulation of all Hsps tested and apoptosis induction, but did not cause DNA damage. Apoptosis in MCF-7 and MDA-MB-231 cells was demonstrated by increased caspase activity and by cleavage of caspase-3 and PARP-1.

## Conclusion

The results suggest that Qu may be an effective agent for the treatment of breast cancer due to leading a significant reduction in Hsp levels and induction in apoptosis. Furthermore, MCF-7 cells were found to be more sensitive than MDA-MB-231 cells against Qu treatments, thus Qu can function as an anti-breast cancer agent with higher potential in ER+ breast cancer than compared to ER- breast cancer cells.

## Keywords

Quercetin,heat shock protein,apoptosis,breast cancer

## Acknowledgement

The authors declare no conflict of interest. This work was supported by TÜBITAK (Project Number: 1919B011502439), Turkey.

# Abstract: Site directed mutagenesis of NAD+-dependent formate dehydrogenase isolated from cotton (*Gossypium hirsutum*)

**Authors:** Reyhan AKKUZU, Emel ORDU

**Affiliations:** Yildiz Technical University, Istanbul, Turkey

## Background/aim

The NAD+-dependent formate dehydrogenases (FDH, EC 1.2.1.2) are important enzymes especially in pharmaceutical industry for the regeneration of NAD(P)H which is an expensive coenzyme used for chiral molecules synthesis. However, FDH enzymes show a rapid inactivation under biotransformation conditions because of the lack of thermostability. Thermal stability is a limiting factor for the development of industrial biotransformation processes. While there is a wealth of data for the effects on plant growth under biotic and abiotic stress conditions, our understanding about stability of plant-derived recombinant FDHs is limited and the biotechnological potential of plant-sourced FDHs need to be evaluated. Therefore, we used site directed mutagenesis method to improve the stability of NAD+-dependent FDH isolated from *Gossypium hirsutum*(GhFDH).

## Materials and methods

In this study M126, M214, M258, M294, M299 and M321 residues were selected to substitute Leu residue by using homology modelling. Primer pairs that carry the desired modification were designed and site directed mutagenesis was performed by using single step PCR. The mutagenesis mixture was transformed into DH5α-T1 E. coli cells. Mutant GhFDHs was sequenced in the region of the mutation to check for correct base chancing.

## Results

Mutant GhFDHs have been overexpressed in *Escherichia coli* and purified. SDS-PAGE showed the proteins to be > 95% pure after purification. Protein concentration was determined using Bradford Protein Assay (Bio-Rad). 1.4 mg/ml protein was obtained from purification.

## Conclusion

Characterization studies of mutants in terms of kinetic and thermal stability properties are still in progress.

## Keywords

NAD^+^-dependent formate dehydrogenase,*Gossypium hirsutum*,site directed mutagenesis,thermal stability

# Abstract: Analyses of p53 interactions with human aprataxin, SACML1 and METAP1 by Y2H system.

**Authors:** Kübra DURMUS, Muhammed DÜNDAR, Ahmet KOÇ

**Affiliations:** Inonu University School of Medicine, Malatya, Turkey

## Background/aim

The p53 transcription factor regulates multiple biological functions, including growth arrest, DNA repair, and apoptosis. p53 activity is regulated by many factors including postt- ranslational modifications. We previously studied to find antioxidant genes that regulate human p53 activity in the heterelogous yeast system. 109 deletion mutants lacking all the antioxidant genes were screened and three of them (Δ*hnt3*, Δ*sac1* and Δ*map1*) were found to be showing lower p53 activity as compared to the wild-type cells. These genes have homologs in humans and they may be important in p53 regulation. Here, we aimed to investigate the relationships between the p53 and human HNT3, METAP1 and SAC1 by yeast two-hybrid analyses.

## Materials and methods

We utilized the yeast two-hybrid technique to dissect the protein-protein interactions between the p53 and the other proteins. We used ProQuest^TM^ Two-Hybrid System (Thermo Fischer) for effcient cloning and expression of GAL-fusion proteins.

## Results

Transformants that contain p53-SAC1, p53-METAP1 and p53-HNT3 gave high β-galactosidase activity along with the p53-LacZ control assay. Statistical analyses revealed insig- nificant differences between the control and test samples.

## Conclusion

Presence of p53 plasmid resulted in positive activity in all the transformants including the controls. We believe that the transcriptional transactivity of p53 interfered with the assay. Thus, we need to re-evaluate the protein interactions by Co-IP analyses in mammalian cells.

## Keywords

p53,protein interaction,yeast two hybrid

## Acknowledgement

This work was supported by TÜBITAK 117Z990 project to AK.

# Abstract: A long noncoding RNA repressed by DNA damage mediates cell survival and proliferation

**Authors:** Gulin BARAN, Irfan Baki KILIC, Nazli KESKIN-TOKLU, Zeynep TOKCAER-KESKIN, Emre DENIZ

**Affiliations:** Acibadem Mehmet Ali Aydinlar University, Istanbul, Turkey

## Background/aim

The Human Genome Project followed by developments in next generation sequencing technologies led to better annotation of the human genome. According to these, more than 70% of the human genome is transcribed, however, only less than 2% of these are translated into final protein products. Long noncoding RNAs (lncRNAs) are transcripts that are longer than 200 nucleotides and that lack an open reading frame. Researchers are brightening the literature about lncRNAs in the various cellular processes and diseases; however, the list is far from being completed.

## Materials and methods

Differentiation Antagonizing Non-Protein Coding RNA (DANCR) is a lncRNA and was found to promote cell growth and tumorigenicity in various cancer types. We identified that DANCR is controlled during DNA damage response. In RNA-seq experiments, we found that upon doxorubicin treatment, DANCR expression is repressed in human colorectal cancer (HCT116) and mouse embryo fibroblast (MEF) cells, suggesting an evolutionary conserved role for this lncRNA.

## Results

Using RT-qPCR, we confirmed that the decrease in DANCR expression is time dependent but it is restored in prolonged treatments. In order to uncover the roles of DANCR in cancer cell growth and proliferation, we deleted DANCR gene in HCT116 cells using CRISPR/Cas9 gene editing technology and confirmed the gene deletion by genotyping PCR and Sanger sequencing. The mutant cells proliferate slower compared to paternal cells; migrate slower; and have greater IC50 value against doxorubicin.

## Conclusion

The identification of molecular mechanisms of DANCR in these cellular processes will shed light onto the DNA damage response mechanisms in colorectal cancer.

## Acknowledgement

This project is supported by grants from TUBITAK (Grant number: 216S404) and Acibadem Mehmet Ali Aydinlar University Scientific Research Project Council (Grant number: 2017/01/10).

# Abstract: Secretion systems of *Xenorhabdus* bacteria associated with entomopathogenic nematodes: A relative analyses

**Authors:** Safak KALINDAMAR, Salih KUMRU, Jochen BLOM, Selcuk HAZIR

**Affiliations:** 1 Ordu University, Ordu, Turke; 2 Recep Tayyip Erdogan University, Rize , Turkey; 3 Justus-Liebig-University Giessen, Giessen , Germany; 4 Aydin Adnan Menderes University, Aydin, Turkey

## Background/aim

Bacteria in *Xenorhabdus* genus are symbiotically associated with obligate insect pathogenic *Steinernema* nematodes. *Xenorhabdus* bacteria secrete toxins, exoenzymes, and proteins for immune evasion and subsequent killing of insects. Some of these proteins are secreted via particu- lar protein secretion systems in entomopathogenic Gram-negative bacteria. However, secretion sys- tems in the entomopathogenic *Xenorhabdus* spp. have not been identified completely. To understand the distribution of secretion systems among the *Xenorhabdus* genomes, we conducted a comprehen- sive comparative analysis of secretion systems (Type I-VI).

## Materials and methods

MacSyFinder (TXSScan), SecReT4, and SecReT6 databases were used to identify secretion systems from the 51 of available complete and draft *Xenorhabdus* genomes in NCBI (National Center for Biotechnology Information). For phylogenetic analysis, average nucleo- tide identity (ANI) values were calculated by EDGAR.

## Results

Our results showed that all analyzed *Xenorhabdus* genomes had Type I (T1SS) and Type V (T5SS) secretion systems and Type IV pili (T4P). None of the *Xenorhabdus* genomes possessed Type II (T2SS) and Type III (T3SS) secretion systems. The complete operon of Type VI secretion system (T6SS) was present in all strains except in the X. thuongxuanensis 30TX1. There was no variation in secretion systems among *Xenorhabdus* genomes.

## Conclusion

This is the first comprehensive analysis on the secretion systems of the *Xenorhabdus* genomes. We expected to improve our understanding of distribution of secretion systems by com- parative analysis of the *Xenorhabdus* genomes.

## Keywords

Comparative genomics,bacterial secretion systems,entomopathogen,*Xenorhabdus*

# Abstract: Functional analysis of a novel hexosaminidase dmHexDC in *Drosophila* eye development

**Authors:** Anastasia FOKINA, Ferdi Ridvan KIRAL, Emir ERKOL, Gökhan ÖZTURAN, Arzu ÇELIK FUSS

**Affiliations:** Bogaziçi University, Istanbul, Turkey

## Background/aim

Glycosylation is an important modification of proteins and lipids. It affects their folding, targeting and the interaction between them. Glycosyltransferases add glycans to proteins and lipids and elongate the chains of glycans while glycosidases are responsible for hydrolysis of glyco- sides and play role in glycan maturation and degradation of the glycated molecules. Hexosaminidases are responsible for cleavage of terminal N-acetyl-β-D-hexosamines. Here we describe the beta-hex- osaminidase CG7985 (dmHexDC), which is expressed in R7 photoreceptors and the morphogenetic furrow during retina development in *Drosophila*.

## Materials and methods

molecular cloning, RNAi, immunohistochemistry, LipiTOX staining, en- zymatic assay, CRISPR/Cas9.

## Results

The downregulation of dmHexDC affects the localization of Wg, Notch, Delta, and Dpp signaling in the developing fly retina. Loss of dmHexDC leads to the neuronal loss through apoptosis which is accompanied by non-neuronal tissue overgrowth. Similar to the homologous human cytoplasmic hexosaminidase HEXD, dmHexDC catalyzes the hydrolysis of the N-acetylglucosamine and N-acetylgalactosamine residues with a bias towards N-acetylgalactosamine. Unlike HEXD, dmHEXDC possesses a transmembrane domain, which suggests its localization to the secretory pathway. HexDC^null^ mutants show enlargement of lysosomes and lipid accumulation. However, our preliminary co-localization studies and dmHexDC optimum activity at pH6.0 suggests localization of dmHexDC to Golgi.

## Conclusion

The loss of dmHexDC in Drosophila causes phenotypes resembling lysosomal storage disorder symptoms in humans, but the localization of dmHexDC together with the process that is crucial for neuronal development and that depends on this enzyme remain unclear. To reveal the role and the localization of dmHexDC, we are performing a search for potential interaction partners of dmHexDC via proximity-dependent labelling technique.

## Keywords

*Drosophila*,hexosaminidase,retina development,R7 photoreceptors,neuronal loss

## Acknowledgement

The work is supported by Bogaziçi University Research Fund projects 14B01M1, 15B01P7 and TÜBITAK (The Scientific and Technological Research Council of Turkey) fellowship 1059B161700281.

# Abstract: Investigation of the anti-alzheimer effect of novel coumarin derivative

**Authors:** Sule IRMAK, Asli SEMIZ, Elif KALE, Fatma Sezer SENOL-DENIZ, Ozden OZGUN ACAR, Gabriela TATARINGA, Ana-Maria ZBANCIOC, Krystyna SKALICKA-WOZNIAK, Ilkay ERDOGAN ORHAN, Alaattin SEN

**Affiliations:** 1 Pamukkale University, Denizli, Turkey; 2 Gazi University, Gazi University, Ankara, Turkey; 3 University of Medicine and Pharmacy, Iasi, Romania; 4 Medical University of Lublin,Lublin, Poland; 5 Abdullah Gül University, Kayseri, Turkey

## Background/aim

Alzheimer disease (AD) is a complex disorder characterised by loss of synapses, causing memory loss and neurodegeneration. At present, there is no definite treatment that is known to prevent the progression of this disease. Coumarin has been proven to be a biological and therapeutic agent in various diseases. It was aimed to investigate the anti-Alzheimer effects of new coumarin derivatives on human neuroblastoma cell line (SH-SY5Y).

## Materials and methods

Coumarin derivatives were synthesised and provided by Dr. G. Tataringa. 4-Propyl-8-hydroxycoumarin (PHC) was selected based on its AChE inhibition activity, among others. The SH-SY5Y cells were cultured in DMEM:F12 medium and the cell viability was assessed using crystal violet staining. EC_04_ and EC_08_ doses were determined for further study. Determined doses of PHC was applied to the cells. Total RNA was isolated, and qRT-PCR analysis was carried out for each gene. The effect of PHC on the expression level of APP, PSEN1, PSEN2, APOE, CLU, CR1, PICALM, BIN1, ABCA7, MS4A1, CD33, CD2AP, SORL1, MMP9 was analysed.

## Results

We have found that the expression of PSEN1, PSEN2, APOE, CLU, PICALM, ABCA7 genes were significantly inhibited while the level of CR1 and MS4A1 genes were significantly induced upon PHC application in SH-SY5Y cells

## Conclusion

Based on the down-regulation of the genes related to Alzheimer disease, it is suggested that PHC poses therapeutic potential for Alzheimer disease.

## Keywords

Anti-Alzheimer,Coumarin,4-propyl-8-hydroxycoumarin,Acetylcholinesterase

## Acknowledgement

This work is supported by Pamukkale University Scientific Research Project Funds with the project no: 2019FEBE021.

# Abstract: miR-185-5p response to usnic acid induces apoptosis in breast cancer cell

**Authors:** Elif DEGERLI, Demet CANSARAN-DUMAN

**Affiliations:** Ankara University, Ankara, Turkey

## Background/aim

Breast cancer is the most common cancer types in woman. Recent research has focused on determining the efficiency of alternative molecules in breast cancer treatment. The aim of this study was to determine the effect of usnic acid response-miR-185-5p relationship with apoptosis pathway.

## Materials and methods

Apoptosis was assessed by using flow cytometry and cell cycle assays. The apoptosis-related genes expression analysis was performed by qRT-PCR and the direct target of miR- 185-5p in BT-474 cells was identified by western blot and luciferase reporter assay.

## Results

The cell apoptosis rate was significantly increased following the ectopic expression of miR- 185-5p in BT-474 cells. Furthermore, the results of cell cycle assay performed by flow cytometry revealed that the transfection with miR-185-5p induced G1/S phase arrest. Our data showed that miR-185-5p can cause significant changes in apoptosis-related genes expression levels, suggesting that cell proliferation was suppressed by miR-185-5p via inducing apoptosis in breast cancer cells. According to western blot results, miR-185-5p lead to decrease BCL2 protein level in BT-474 cells and direct target of miR-185-5p was identified as BCL by luciferase reporter assay.

## Conclusion

This study revealed that miR-185-5p may be an effective agent in the treatment of breast cancer.

## Keywords

miR-185-5p,usnic acid,breast cancer,apoptosis,BCL2

## Acknowledgement

We thank Ankara University, Management of Scientific Research Projects (Proj- ect no. 15B0415001), for financial support.

# Abstract: Effect of β-Hydroxybutyrate in aging process *Schizosaccharomyces pombe*.

**Authors:** Hande ULUDAG, Çagatay TARHAN, Özgür ÇAKIR, Melis ATAÖZÜ

**Affiliations:** Istanbul University, Istanbul, Turkey

## Background/aim

Ketone bodies are the group of water-soluble molecules that are produced by the liver especially from fatty acids. The effect of ketone bodies on the longevity or aging process has been studied for years in a wide range of organisms. Although some positive effect of different kind of ketone bodies has been shown in different studies, the underlying molecular mechanisms remain debated and need to be clarified. Here, we aimed to study the effect of a ketone body, β-Hydroxybutyrate, on the longevity of *Schizosaccharomyces pombe* and tried to shed more light on the mechanisms that contributed the increased longevity.

## Materials and methods

*S.pombe* cells were treated with different concentrations of β-Hydroxybutyrate and the results were compared with non-treated *S.pombe* cells. To investigate the β-Hydroxybutyrate treatment on certain cellular paramaters, carbohydrate consumption, total cellular oxidation, lipid peroxidation, stress response to heat and H_2_O_2_ have been measured. Besides, the effect of β-Hydroxybutyrate on 23 different *S.pombe* mutants that are defective in glucose uptake process was investigated.

## Results

β-Hydroxybutyrate extended the lifespan of *S.pombe* cells 20%. β-Hydroxybutyrate treated cells used less carbohydrate and had significantly less lipid peroxidation. On the other hand β-Hydroxybutyrate extended the lifespan of 6 mutants related with glucose uptake mechanism.

## Conclusion

Ketogenic diet has a considerable effect on the longevity of *Schizosaccharomyces pombe* cells. The results support that this effect may be caused by a decrease in glucose uptake and beneficial effect on longevity may especially be related with intracellular oxidative condition.

## Keywords

Aging,β-Hydroxybutyrate,ketone metabolism,*Schizosaccharomyces pombe*.

## Acknowledgement

This research is supported by Istanbul University Scientific Research Projects Unit (BAP) project number 33015.

# Abstract: Association between Parkinson disease and vitamin D receptor (VDR) Taq-I polymorphism in the Turkish population

**Authors:** Kadir Sinan ARSLAN, Halil Ibrahim KOC, Ahmet Yahya HOSCAN, Ahmet ARMAN

**Affiliations:** Marmara University, Istanbul, Turkey

## Background/aim

Parkinson Disease (PD) is the second most common neurodegenerative disease. The symptoms of PD are tremor, akinesia, bradykinasia, hypomimia, decreased eye blinking, and hypophonia. The pathogenesis of Parkinson Disease (PD) remains poorly understood. The purpose of this study was to determine the relationship between *VDR* Taq-I polymorphism and PD in the Turkish population.

## Materials and methods

This study contains 152 PD patients and 184 healthy controls. Genomic DNA's were isolated from the blood samples of each subject by salting out method. *VDR* gene Taq-I polymorphism region was amplified by polymerase chain reaction (PCR). The PCR products were digested by Taq-I enzyme and genotype of patients and healthy control was determined by size of the digested PCR products.

## Results

No Significant association for genotype distribution of *VDR* Taq-I polymorphism was found between PD patients and healthy control in Turkish population. Also, there is no relationship for allele frequency of *VDR* Taq-I between PD patients and healthy controls too.

## Conclusion

There is no significant association with *VDR* Taq-L polymorphism (p>0,05) and Parkinson's Disease in Turkish population.

## Keywords

*VDR*,Taq-I ,Polymorphism,PD

## Acknowledgement

This project was supported by GEN ERA Diagnostic.

# Abstract: Assessment of cytotoxic and apoptotic activities of the essential oil from *Salvia syriaca*

**Authors:** Kübra KOCABIYIK, Dogukan MUTLU, Sevki ARSLAN, Gürkan SEMIZ

**Affiliations:** Pamukkale University, Denizli, TURKEY

## Background/aim

*Salvia* species have received a great deal of attention due to their economic importance and their wide diverse biological activities including anti-tumor, anti-carcinogenic, antioxidant activities. In this regards, the aim of this study is to determine cytotoxic and apoptotic effects of essential oils obtained from aerial parts of *Salvia syriaca* L. and their chemical content by GC-MS.

## Materials and methods

*Salvia syriaca* were collected from Burdur-Turkey. Essential oil was obtained by water distillation using a Clevenger apparatus. Different concentration of essential oils was applied to human colon cancer cell line (Caco-2) for 24 hours. At the end of 24 hours, the survival rate of cell was measured crystal violet test at 630 nm by using ELISA reader. The Annexin V-FITC Apoptosis Assay Kit was used to evaluate cell apoptosis.

## Results

The GC-MS analysis showed that germacrene D (21.77%), trans-β-ocimene (14.66%), β-pinene (9.07%), α-cadinol (8.19%) and α-pinene (6.50%), were major components found in essential oil. Moreover, that oil showed cytotoxic effect on Caco-2 cells in a dose-dependent manner. The EC50 value of the essential oil was found to be 63.5 μg/ml. Image based cytometry analysis revealed that the percentage of apoptosis was increased after exposure of Caco-2 cells to *S. syriaca* essential oils. After oil treatment, annexin positive cells increased around 6-fold with respect to control cells.

## Conclusion

All those data suggest the hypothesis that essential oil of S. syrica is relatively cytotoxic and apoptotic to Caco-2 cells and has promising phytochemicals that may be used in cancer treatment. Activity guided fractionation experiment should be done for testing that hypothesis.

## Keywords

*Salvia syriaca*,cytotoxic and apoptotic activity,GC-MS,essential oil,Caco-2

## Acknowledgement

This study was supported by Scientific Research Coordination Unit of Pamukkale University under the project number 2018KRM011 and 2019KRM004.

# Abstract: Terpinolene induces apoptosis in human breast cancer cell lines

**Authors:** Cenk KIG, Hizlan HINCAL AGUS, Varol GÜLER, Alper ÇALISKAN, Elif MERTOGLU, Evren ÖNAY UÇAR

**Affiliations:** 1 Istanbul Yeni Yuzyil University, Istanbul, Turkey; 2 Istanbul University, Istanbul, Turkey

## Background/aim

Findings suggest that terpinolene can suppress proliferation and induce cell death in cancer cells. However, pathways and mechanisms involved in terpinolene-induced cell-death are not clear. In this study we aimed to investigate the changes in cell viability and the expression of some apoptotic genes in response to terpinolene treatment in ER^+^ or ER^-^ cells.

## Materials and methods

Firstly, using MTT (3-(4,5-dimethylthiazole-2,5-diphenyltetrazolium) assay we compared the effect of terpinolene on viability of ER^+^ (MCF7), ER- (MDA-MB-231) breast cancer cells. HEK- 293 was used as a non-cancerous control cell. Terpinolene-induced changes in the expression levels of apoptotic proteins (Bax and cleaved PARP) were determined by Western blot assay. Our findings on apoptosis were further tested by Annexin V/PI staining.

## Results

Cisplatin was used as a positive control agent for evaluating anti-cancer effects of terpinolene. ER^TR^+ breast cancer cell line MCF-7 (IC_50_: 43.77μM, 24 hours) was more sensistive to cisplatin than ER^TR^- MDA-MB-231 cell line (IC_50_: 78.42 μM, 24 hours). Non-cancerous cell line HEK-293 was also found to be sensitive to cisplatin (IC_50_: 44.74 μM). Similarly, MCF-7 cells were the most sensitive to terpinolene treatment (IC_50_: 528.67 μM, 24 hours) while MDA-MB-231 cell line displayed a moderate resistance to terpinolene treatment (IC_50_: 636.54 μM, 24 hours). HEK-293 cells were found to be the most resistant (IC_50_: 709.92 μM, 24 hours). Data obtained from both Western blot and RT-qPCR suggested that terpinolene induced the expression of genes involved in apoptotic pathways.

## Conclusion

Terpinolene induces cell death probably through induction of apoptosis. When compared to the ER^-^ MDA-MB-231 cell, ER^+^ cell MCF7 was more sensitive to terpinolene treatment. Unlike cisplatin, noncancerous HEK-293 cells showed strong resistance against terpinolene.

## Keywords

Terpinolene,Breast Cancer,Apoptosis,Cisplatin

## Acknowledgement

This work was supported by TÜBITAK, Turkey (Project Number: 118S206).

# Abstract: Angiogenesis-related proteins sFlt-1 and PlGF in preeclampsia

**Authors:** Vildan YAMAN, Ali BENIAN, Nazli ARDA

**Affiliations:** 1 Istanbul University, Istanbul, Turkey; 2 Istanbul University-Cerrahpasa, Istanbul, Turkey

## Background/aim

Preeclampsia is a pregnancy specific syndrome. Studies have suggested that a high ratio of soluble fms-like tyrosine kinase-1 (sFlt-1, an anti-angiogenic factor) to placental growth factor (PlGF, an angiogenic factor) is related to pathophysiology, and can be used in the diagnosis of preeclampsia. Aim of this study is to check the plasma concentrations of these factors, and their ratio in preeclamptic (PE) and healthy (control) pregnant subjects.

## Materials and methods

Plasma samples were collected from patients with preeclampsia and healthy pregnant between the years 2016-2018. The concentration of sFlt-1 and PlGF were measured by ELISA.

## Results

Mean sFlt-1 levels were 13.682.015 ng/mL (n=19) and 9.6440.7177 ng/ mL (n=18) in control and PE groups, respectively. Mean PlGF level was 0.490.0788 ng/mL in control group while 0.35890.02905 ng/mL in PE group. Thus, sFlt-1/PlGF ratio was calculated as 29.311.06 and 27.751.665 in control and PE groups, respectively. Although PlGF level was lower in PE group in comparison to healthy group as expected, significantly high level of sFlt-1 was detected in healthy pregnant surprisingly. There was no statistically significant difference in the sFlt-1/PlGF ratio be- tween two groups.

## Conclusion

Many studies have concluded that sFlt-1 and sFlt-1/PlGF ratio are higher in preeclamp- sia patients than healthy pregnant subjects. On the contrary, we found that sFlt-1 was higher in healthy pregnant subjects, and there is no significant difference in sFlt-1/PlGF ratio. PlGF was lower in preeclampsia patients, in consistent with the results of other studies. Further studies are needed to clarify whether the plasma levels of sFlt-1, PlGF and their ratio are useful markers in the diagnosis of preeclampsia.

## Keywords

Preeclampsia,soluble fms-like tyrosine kinase-1,placental growth factor

## Acknowledgement

This study was funded by Scientific Research Projects Co- ordination Unit of Istanbul University, Project Number: 22922.

# Abstract: Evaluating the role of microenvironment in efficacy of Etanercept

**Authors:** Mona SALIMI, Elnaz SHIRMOHAMMADI, Farnaz BARNEH

**Affiliations:** 1 Pasteur Institute of Iran, Tehran, Iran; 2 Tehran University of Medical Sciences, Tehran, Iran; 3 Shahid Beheshti University of Medical Sciences, Tehran, Iran

## Background/aim

Interaction between microenvironment and breast cancer cells is not routinely considered at the early stages of drug development leading to failure of many drugs at later clinical stages. Here, we used systems biology approach to predict the efficacy of Etanercept, which was further confirmed by the molecular biology experiments.

## Materials and methods

We extracted secretome data of MDA-MB-231 cells from public repositories and performed gene enrichment analysis using the library of LINCS L1000 ligand perturbations. We also used Boolean network to model changes in the dynamics of intracellular signaling pathways in the presence and absence of macrophages. MTT assay, apoptosis and cell cycle progression evaluations, Matrigel method along with Real-time PCR were used for the molecular biology assessment. Moreover, we investigated the effect of breast cancer and macrophage co-culturing on the efficacy of Etanercept.

## Results

The secretome profile was found to be similar to the expression of genes following treatment of breast cancer cells with TNF-α. Inhibition of TNF-α using Etanercept decreased survival of MDA- MB-231 cells and induced apoptosis as well as cell cycle arrest in vitro through inhibition of NFKB. We observed that the inhibitory effect of Etanercept on cell viability, cell cycle progression, invasion and induction of apoptosis decreased when cancer cells were co-cultured with macrophages. The results showed that macrophage cytokines and chemokines activated NFKB leading to the decreased efficacy of Etanercept suggesting that inhibition of NFKB may be an alternative approach to inhibit cancer cell growth in presence of macrophage crosstalk.

## Conclusion

Our study provides an approach to predict the effect of drugs in the presence of stromal cells to guide experimental designs in drug development.

## Keywords

Breast cancer,Co-culturing,Etanercept

# Abstract: A genome-wide screening of fluconazole resistant mutants in *Schizosaccharomyces pombe*

**Authors:** Burcu KARTAL, Merve YILMAZER, Bedia PALABIYIK

**Affiliations:** Istanbul University, Istanbul, Turkey

## Background/aim

Invasive fungal infections are hidden killers that stem from several fungal spe- cies such as *Candida*. Fluconazole is a well-known triazole drug with antifungal activity and widely used against *Candida* infections. It is important to identify genes affecting sensitivity and resistance to antifungal drugs. In this study, we screened the haploid mutant collection of *Schizosaccharomyces pombe* with fluconazole to determine potential target genes affecting the drug activity.

## Materials and methods

*S. pombe* deletion mutant library (haploid 3,400 strains) were supplied by BiONEER. The parental strain, ED666 (h^+^ ade6-M210 ura4-D18 leu1-32) have been used for deter- mination of effective fluconazole concentration by counting the colony forming units and vitality of cells. The screening of resistant strains was carried out in three steps by measuring optical density of control and experimental groups at 600 nm. Database searches were performed using the Schizosac- charomyces pombe genome database (PomBase) and the *Candida* genome database (CGD).

## Results

Using genome-wide screening we determined that 130 deletion strains in the library are fluconazole resistant. We found that 76 of 130 mutants have *Candida albicans* orthologous and clas- sified into various biological processes based on the functions of the proteins that the genes encode. Protein modification by small protein conjugation or removal, transmembrane transport and signal- ling are remarkable processes among them.

## Conclusion

Similarity of features with pathogenic fungi make *S. pombe* excellent model organism to study the activity of antifungal drugs. We performed a genome-wide screen for fluconazole that providing the opportunity for resistance development. According to our results, genes that are deleted in the resistant mutants may be considered as new targets for studying the resistance mechanism of fluconazole.

## Keywords

*Schizosaccharomyces pombe*,fluconazole,deletion library,*Candida albicans*

## Acknowledgement

This work was supported by Scientific Research Project Coordination Unit of Istanbul University. Project number: 51649.

# Abstract: PICUS: Pointed interpretation of clinical variant significance

**Authors:** Baris SALMAN, Sibel Ugur ISERI

**Affiliations:** 1 Gen-Era Diagnostik, Istanbul, Turkey; 2 Istanbul University, Istanbul, Turkey

## Background/aim

Analysis of genetic variations have have identified molecular basis for over 5000 diseases through the past decades. Growing knowledge on inherited diseases and gradual decrease in the cost of next generation sequencing (NGS) applications have made NGS based genetic tests feasible for genetic diagnosis. However, the emergence of NGS in the era of clinical genetics comes with various challenges. One of those challenges is assessing the significance of genetic variations in the human genome. American College of Medical Genetics and Genomics (ACMG) and the Associa- tion for Molecular Pathology (AMP) guide has been widely used as standard for sequence variant interpretation based on 28 criteria. However, there is significant variance when it comes to implemen- tations of those criteria among different institutes and genome analysts. We have therefore set out to automatize ACMG/AMP criteria via developing a novel bioinformatics tool, namely 'picus'.

## Materials and methods

Picus is developed using Python with the Pandas module. After process- ing raw fastq data into varant call format (vcf), vcf is further annotated using Ensembl Variant Effect Predictor (VEP) tool. Parameters such as disease and allele frequency, domain structure of proteins, in silico prediction metrics and documented variant data retrieved from ClinVar have been used for automatized processing of ACMG/AMP criteria prediction.

## Results

Picus v0.0.4 can automate interpretation of 13 ACMG/AMP criteria in order to classify variants based on significance. Manual interpretation is required to incorporate individual of family specific metrics such as segregation data and *de novo* status of variants.

## Conclusion

There is high demand for customized bioinformatics tools that could be used in the medical genetics area due to growing number of NGS based clinical genetic tests. Picus aims to help genome analysts in this concept via increased automation in ACMG/AMP criteria.

## Keywords

Next generation sequencing,medical genetics,bioinformatics,automation of ACMG/ AMP criteria

# Abstract: Analysis of m6A RNA modification patterns in apoptosis-induced Hela cells

**Authors:** Ayse Bengisu GELMEZ, Azime AKÇAÖZ, Ipek ERDOGAN, Bünyamin AKGÜL

**Affiliations:** Izmir Institute of Technology, Izmir, Turkey

## Background/aim

Apoptosis is one of the essential mechanisms for cellular homeostasis. Several post-transcriptional and transcriptional processes are shown to have regulatory function in apoptosis. However, the contribution of RNA methylation to this fundamental process still remains unclear. Since N6-methyladenosine (m6A) RNA modifications are known to be one of the most abundant RNA modifications thus far, in this study, we aimed to investigate m6A methylation patterns in apop- totic HeLa cells.

## Materials and methods

HeLa cells were treated with cisplatin (CP), a chemotherapeutic drug and TNF-alpha recombinant protein to activate intrinsic and extrinsic apoptotic pathways, respectively. After determination of apoptosis rate by flow cytometry, samples were subjected to m6A-iCLIP- seq analysis to determine the extension of m6A RNA modifications compared to the control group (DMSO 0.1%).

## Results

Single-base resolution m6A-iCLIP analysis revealed the differential m6A RNA modifica- tions in various RNA types such as mRNAs, lncRNAs, and miRNAs related with apoptosis. In total, 58-apoptosis-related genes contains enriched m6A modifications upon cisplatin treatment while this number was 92 in TNF-alpha treated cells. Interestingly, the methylation enrichment observed on 5'UTRs in cisplatin treated cells, while TNF-alpha treatment primarily led to methylation on 3'UTRs.

## Conclusion

To our knowledge, this is the first report of m6A modification analysis of RNAs under different apoptotic conditions that should shed light, upon additional functional experiments, onto the potential functional effects of such modifications under apoptotic conditions.

## Keywords

Epitranscriptomics,N6-methyladenosine,RNA modifications,apoptosis

## Acknowledgement

This study was funded by TUBITAK (The Scientific and Technological Research Council of Turkey) (Project no: 217Z234)

# Abstract: The effects of temozolomide in exosomal stress proteins in glioma cells

**Authors:** Cansu KILCI, Aslihan SENGELEN, Murat PEKMEZ

**Affiliations:** Istanbul University, Istanbul, Turkey

## Background/aim

Glioblastoma (GBM) is highly aggressive primary brain malignancy with high mortality rates. Heat shock proteins (Hsp) are overexpressed in GBM, they are effective in the tumor cell proliferation and progression. Exosomal Hsp70 is the key role in tumor growth, cell migration and metastasis particularly. Temozolomide (TMZ) is a chemotherapy agent for GBM treatment. The effect of TMZ on exosomal Hsp70, incompletely understood yet. The aim of this study was to investigate the effect of TMZ on exosomal and cellular Hsp70 expression profile and tumor cell migration.

## Materials and methods

The effect of TMZ on cell proliferation was determined by MTT assay. Exosome isolation was confirmed by Western blotting and Scanning Electron Microscopy. The effect of TMZ on Hsp70 was evaluated by Western Blotting. Cells migration rate was assessed by in vitro scratch assay. The statistical analysis was performed with one-way ANOVA followed by the Tukey post-hoc-test. The significance limit was accepted P < 0.05.

## Results

TMZ treatments (25 and 200 μM) resulted increment in intracellular Hsp70, resulting reduction in exosomes, and Hsp70 levels decreased both in the cell and in the exosome at 750 μM TMZ. After 48 h treatment with TMZ, cell migration rate decreased in dose-dependent manner.

## Conclusion

Exosomal Hsp70 levels reduced by TMZ whereas cellular Hsp70 expression increa- sed in a dose-dependent manner. This is closely related to the cell's mechanism of resistance to the drug. Also, we observed that cell migration ability slows as the concentration of TMZ increases. The reduction of migration rate confirms that TMZ is an important anticancer drugs.

## Keywords

Exosome,temozolomide (TMZ),glioblastoma (GBM),heat shock protein (Hsp)

## Acknowledgement

This work was supported by the Istanbul University Research Foundation, Turkey (Project Number:29087).

# Abstract: Determination of therapeutic potential of fisetin for acute lymphoblastic leukaemia cells

**Authors:** Mustafa OZTURK, Sevim Beyza GURLER, Yagmur KIRAZ, Yusuf BARAN

**Affiliations:** Izmir Institute of Technology, Izmir, Turkey

## Background/aim

Acute Lymphoblastic Leukaemia (ALL) is a malignant hematologic cancer char- acterized with increased number of genetically and/or epigenetically inhibited lymphoblast cells in bone marrow. Reciprocal translocation of *BCR gene* on chromosome 22 and *ABL genes* on chromo- some 9 leads Philadelphia (Ph) chromosome which has aberrant tyrosine kinase activity occurred in ALL (Ph+ ALL). Tyrosine kinase inhibitors are frequently used in addition to chemotherapy. Due to complete cure could not be succeeded and current strategies only help to prolong lifetime of Ph+ ALL patients, new strategies are needed for therapy. Fisetin (7, 3', 4 fit-flavon-3-ol) is a flavonoid and naturally found in many vegetables and fruits. Studies demonstrate fisetin have anti-oxidant and anti- cancer effect on several cancer types including breast, lung, prostate cancers and leukemia. In this study, therapeutic potential of fisetin on Ph+ ALL cells will be determined for the first time.

## Materials and methods

Cytotoxic effect of Fisetin on Ph+ ALL cells (SD-1 cell line) and normal epithelial cells (Beas-2B) was determined dose and time dependently by using MTT assay, Trypan- Blue cell viability assay and DAPI/PI staining. Our ongoing studies will determine the cytostatic and apoptotic effects of fisetin on SD-1 cells by Cell Cycle Analysis and AnnexinV-Propidium Iodide Double Staining through using flow cytometry.

## Results

According to our MTT Assay results, IC50 value of fisetin for SD-1 cells was determined as 30 μM while fisetin has not cytotoxic effect on Beas 2B cell line in both 24 and 48 hours. Moreover, Trypan-Blue and DAPI Staining confirmed the MTT results.

## Conclusion

As results, Fisetin has cytotoxic and anti-proliferative effect on SD-1 cells while fisetin has not cytotoxic effect on Beas-2B cells. Our ongoing studies will determine the dose and time de- pendent cytostatic and apoptotic effects of fisetin on Ph+ ALL cells.

## Keywords

Fisetin,Acute Lymphoblastic Leukaemia,apoptosis

# Abstract: Fanconi anemia due to homozygous deletion in *FANCA* diagnosed by evaluating low coverage regions

**Authors:** Özlem AKGÜN DOGAN, Yasemin KENDIR DEMIRKOL, Merve SAY, Tugba KIZILBOGA AKGÜN, Levent DOGANAY

**Affiliations:** 1 Umraniye Education and Research Hospital, Istanbul, Turkey; 2 Gen-Era Diagnostics, Istanbul, Turkey

## Background/aim

Fanconi anemia is characterized by bone marrow failure, increased malignancy risk and physical abnormalities which present in 75% of affected individuals. Progressive bone mar- row failure typically presents in the first decade resulting in acute myeloid leukemia in the 13% of the patients by age 50 years. Hematopoietic stem cell transplantation is the only curative therapy. Identification of the pathogenic variants in FA associated genes allows the confirmation of the disease especially in patients without any physical anomaly. A 15-year-old patient with no previous health problems was evaluated because of pancytopenia. Physical examination was normal except for microcephaly, deeply located eyes and slightly proximal placement of thumbs. Pancytopenia persisted at the end of three months of follow-up. Fanconi aplastic anemia was in the differential diagnosis list so he was referred to our department for a multigene panel.

## Materials and methods

Clinical exome sequencing was planned. Sequencing was carried out using Illumina V2 chemicals on the Illumina NextSeq 500 platform. Bioinformatics analyzes and variant calling were performed by using Sophia-DDM-V3 bioinformatics analysis program.

## Results

No pathogenic variants associated with the phenotype were found. However, when low cov- erage areas were examined, it was found that reads of the *FANCA* gene exon 15 to 21 were extremely low. This raised suspicion of a homozygous deletion in this region. In deletion/duplication analysis of the *FANCA* by MLPA, homozygous deletion containing exon15-21 was detected.

## Conclusion

By reporting this case, we would like to note that the detailed evaluation of low-cover- age regions may be a clue to identify possible CNVs even in CES analyzes where CNV analysis can not be performed.

## Keywords

Fanconi,*FANCA*,Copy Number Variation,Low coverage

# Abstract: CRISPR/Cas9 based identification of epigenetic factors to overcome therapy resistance in triple negative breast cancer

**Authors:** Ozlem YEDIER-BAYRAM, Alisan KAYABOLEN, Ali Cenk AKSU, Tunc MOROVA, Firat UYULUR, Nathan LACK, Tamer ONDER, Tugba BAGCI-ONDER

**Affiliations:** Koç University, School of Medicine, Istanbul, Turkey

## Background/aim

Triple negative breast cancer (TNBC) is a subtype of breast cancer that does not express receptors for estrogen, progesterone and Her2. It has poor prognosis and lacks targeted thera- pies. Surgery followed by chemotherapy is the primary established systemic treatment for early stage and advanced TNBC. However, developing resistance to chemotherapy is a major obstacle in clinics that usually results in relapse and metastasis. Epigenetic modifications contribute to the development and maintenance of chemotherapy resistant phenotype in different cancers. Therefore, a systematic interrogation of the roles of epigenetic modifiers in chemotherapy resistance in TNBC is crucial to design more effective therapies.

## Materials and methods

We designed an Epigenetic Knock-out Library (EPIKOL), which utilizes the CRISPR-Cas9 technology to study the functions of a wide variety of epigenetic modifiers in chemotherapy-sensitive and -resistant TNBC cell lines. EPIKOL is composed of 8000 gRNA (10 gRNA per gene) targeting the 5' exons of genes encoding chromatin- and DNA-modifying proteins. We utilized MDA-MB-231, SUM159 and SUM149 cell lines to generate chemotherapy-resistant TNBC models. To this end, we exposed these cells to escalating doses of doxorubicin and taxol, the most widely used clinical chemotherapeutics, and selected resistant subpopulations.

## Results

Paired transcriptome analysis of chemoresistant cells revealed differences in epigenetic landscape that result from transition from a chemosensitive to chemoresistant state. Through EPIKOL screens on chemosensitive TNBC cells, we have identified novel epigenetic modifiers that are crucial for cancer cell fitness.

## Conclusion

Further screens with our EPIKOL gRNA library on chemoresistant cells will allow us to differentiate novel epigenetic regulators of chemotherapy resistance from cancer fitness genes in TNBC. These findings can lead to combination therapies targeting resistance-causing epigenetic factors.

## Keywords

Epigenetics,CRISPR/Cas9,breast cancer,drug resistance

## Acknowledgement

This work was supported by The Scientific and Technological Research Council of Turkey (TUBITAK) 1003- 216S461 Grant.

# Abstract: Identification of SERPINE1 as a regulator of glioblastoma cell dispersal with a novel profiling approach

**Authors:** Fidan SEKER, Ahmet CINGOZ, Ilknur SUR-ERDEM, Nazli ERGUDER, Alp ERKENT, Firat UYULUR, Myvizhi Esai SELVAN, Zeynep Hülya GÜMÜŞ, Mehmet GONEN, Halil BAYRAKTAR, Hiroaki WAKIMOTO, Tugba BAGCI-ONDER

**Affiliations:** 1 Koç University School of Medicine, Istanbul, Turkey; 2 Icahn School of Medicine at Mount Sinai, New York, USA; 3 Istanbul Technical University, Istanbul, Turkey; 4 Harvard Medical School, Boston, MA, USA

## Background/aim

High mortality rates of glioblastoma (GBM) patients are partly attributed to the invasive behavior of tumor cells that exhibit extensive infiltration into adjacent brain tissue, leading to rapid, inevitable, and therapy-resistant recurrence. Understanding the mechanisms of GBM cell invasiveness is the priority for the development of successful therapies. This study aims to provide a motility signature of GBM cells as well as to identify possible target genes to limit the dispersal of the cells.

## Materials and methods

We used an in vitro spheroid dispersal model and analyzed transcriptome of motile (dispersive) and non-motile (core) GBM cells. Genetic or pharmacological inhibition of SERPINE1 was used in in vitro assays of spheroid dispersal, cell adhesion and cell movement tracking. TCGA glioma datasets and a patient-derived orthotopic GBM model were used to assess the significance of SERPINE1 in GBM growth and invasion.

## Results

We observed upregulation of gene sets linked to cell cycle and epithelial-mesenchymal transition (EMT) and identified *SERPINE1* as a remarkably induced gene in dispersive cells. Genetic or pharmacological inhibition of SERPINE1 led to reduction of dispersal of GBM cells. We demonstrated that SERPINE1 regulates cell-substrate adhesion and directional movement of GBM cells. *SERPINE1* expression was associated with poor prognosis and mesenchymal GBM in patients. SERPINE1 knock-down in primary GBM cells suppressed tumor growth and invasiveness in the brain.

## Conclusion

To our knowledge, our study provides the first functional demonstration of a direct role of SERPINE1 in GBM cell motility as well as a pro-tumorigenic role in in vivo GBM models. Therefore, our work identifies SERPINE1 as a potential target for future anti-invasive GBM therapies.

## Keywords

GBM,RNA-sequencing,SERPINE1,dispersal,adhesion

## Acknowledgement

The Scientific and Technological Research Council of Turkey (TUBITAK) (315S161, TBO), Koç University (SF.00024, TBO). Koç University Research Center for Transla- tional Medicine (KUTTAM). LUNGevity foundation (ZHG, MS).

# Abstract: Silencing of HSF1 increases the apoptosis induction in human glioblastoma cells upon rosmarinic acid treatment

**Authors:** Aslihan SENGELEN, Evren ÖNAY UÇAR

**Affiliations:** Istanbul University, Istanbul, Turkey

## Background/aim

Glioblastomas (GBM) are the most common and malignant primary brain tumors. High levels of heat shock proteins (Hsp) and their transcription factor heat shock factor 1 (HSF1) in gliomas are closely associated with tumor cell proliferation. Rosmarinic acid (RA), a natural com- pound, exhibits antitumor effects against GBM, but there are no reports regarding its effect on HSF1 expression in gliomas. The aim of the present study was to investigate the effect of RA on HSF1 expression and apoptosis in non-transfected and transfected GBM cells.

## Materials and methods

MTT assay was used to measure the cytotoxic effects of RA and siRNAs. Transfection with specific siRNAs was performed for the HSF1 gene silencing. RA (80 and 215 μM) was administered alone or in combination with siRNAs (25 and 50 nM) to U87MG human glioblas- toma cells. The effects of the treatments on the levels of HSF1, several Hsps and apoptosis-related proteins were evaluated by Western blot analysis. Differences among groups were tested by oneway ANOVA followed by Tukey post-hoc-test.

## Results

We found that RA blocks cell proliferation in a dose- and time-dependent manner. HSF1 siRNA administration alone caused a 70% reduction in HSF1 levels, while combined treatment with RA resulted in almost depletion of the protein. All treatments caused the downregulation of the tested Hsp expression levels and triggered apoptosis. Moreover, our results showed that HSF1 inhibition makes U87MG cells extremely vulnerable to apoptosis induction upon RA treatment.

## Conclusion

Our results suggest that RA administration in combination with HSF1 silencing has the potential to be used as a candidate for GBM treatment.

## Keywords

Rosmarinic acid (RA),heat shock factor 1 (HSF1),heat shock protein (Hsp),apoptosis,glioblastoma (GBM)

## Acknowledgement

The authors declare no conflict of interest. This work was supported by the Istanbul University Research Foundation, Turkey (Project Number: FDK-2019-32757).

# Abstract: Amyloid-beta induced zebrafish model of Alzheimer's disease may offer a new option for drug development

**Authors:** Yusuf Kaan POYRAZ, Gökhan CUCUN, Günes ÖZHAN

**Affiliations:** 1 Dokuz Eylul University, Izmir, Turkey; 2 Izmir International Biomedicine and Genome Center, Izmir, Turkey

## Background/aim

Alzheimer's disease is the most widespread neurodegenerative disorder that causes loss of synaptic connections, neuronal death, and memory deficits. In this study, we aim to generate an amyloid β42(Aβ42)-dependent neurotoxic model in the adult zebrafish brain and validate the AD-like pathology of this model using markers of apoptosis, immune response and amyloidosis. This model will later be used to test the efficiency of a specific peptide as a candidate drug for treat- ment of AD using solid lipid nanoparticles as the peptide delivery system.

## Materials and methods

Aβ42 peptide was injected into the adult zebrafish brain using cerebroventricular microinjection (CVMI) technique. To determine the localization of Aβ42 toxicity in the brain, we performed immunohistochemistry by using an antibody against Aβ42 and acquired images with a confocal microscope. We also examined expression of AD-related genes using quantitative real-time PCR (qPCR).

## Results

We have developed a zebrafish model of AD using CVMI and measured expression levels of AD-related genes including *Bace, Psen, App, Pcna* and *Gfap* in the telencephalon. Our results also confirm the aggregation of Aβ42 in the brain and mark an increase in the number of proliferating cells.

## Conclusion

We believe that zebrafish AD model will emerge as a useful platform to both understand the molecular mechanism of the disease and test the attenuating effects of drug candidates that can constitute a promising therapeutic approach rather than the existing symptomatic treatment of AD.

## Keywords

Alzheimer's disease,zebrafish,amyloid-β42,neurodegenerative diseases

## Acknowledgement

EMBO Installation Grant, TUBITAK 215Z365

# Abstract: Possible relationship between mevalonate kinase and cell migration from perspective of mevalonate kinase deficiency

**Authors:** Tayfun Hilmi AKBABA, Z.Yeliz AKKAYA-ULUM, Selcan DEMIR, Seza ÖZEN, Banu PEYNIRCIOGLU

**Affiliations:** Hacettepe University, Ankara, Turkey

## Background/aim

Mevalonate Kinase Deficiency (HIDS/MKD, MIM#260920) is an ultra rare, autoinflammatory syndrome caused by mutations in MVK gene. Recurrent attacks are seen in patients with abdominal pain, lymphadenopathy and increased immunoglobulin D (IgD). Treatment approach is anti-IL-1 and anti-TNF administration. In this study, we aimed to analyze cell migration which is an important process in inflammation by using primary cells of MKD patients.

## Materials and methods

Peripheral blood mononuclear cells were isolated from 2 MKD, 11 FMF patients (as disease control) and 7 healthy controls. LPS was used to induce inflammation. After activation of inflammation, transwell experiments were performed with isolated cells. Migrating alive cells were stained with calcein-AM and analyzed by ImageJ programme.

## Results

MKD patients' cells did not migrate under normal or stimulated conditions. We observed increased cell migration in FMF patients compared to controls. The possible effect of anti-IL-1 treatment, the drug used for MKD patients, on cell migration was eliminated due to the observation of increased cell migration in minor FMF patients having the same treatment.

## Conclusion

Loss of function in mutant mevolanate kinase enzyme leads to a reduction in the amount of geranylgeranyl pyrophosphate required for protein prenylation. The regulation of many biological processes by protein prenylation and the prenylation of actin-associated proteins (Rho, Rac1, Rab, etc.), which play an important role in cell migration, reinforces the hypothesis that mevalonate kinase may be effective in this process. This study is the first study to demonstrate the possible relationship between mevalonate kinase and cell migration from the MKD perspective.

## Keywords

Mevalonate kinase deficiency,cell migration,prenylation

## Acknowledgement

This project was supported by Hacettepe University Scientific Research Coordination Unit (Project Number: TYL-2018-17354).

# Abstract: Comparison of different chimeric antigen receptor designs for retargeting NK-92 cells against tumor antigens

**Authors:** Elif ÇELIK, Didem ÖZKAZANÇ ÜNSAL, Cevriye PAMUKCU, Tolga SÜTLÜ

**Affiliations:** Sabanci University, Istanbul, Turkey

## Background/aim

Chimeric Antigen Receptor (CAR) mediated targeting of tumor antigens is a promising approach for adoptive immunotherapy. Retargeting Natural Killer (NK) cells by CAR ex- pression is clinically tested and carries the potential to translate into clinical application. This study aims to develop novel and practical CAR vector design and use NK-92 cells for evaluation of dif- ferent designs in order to optimize a CAR vector that could be efficiently used to retarget NK cells against tumour antigens.

## Materials and methods

CAR transgenes comprising identical antigen binding domains that target CD19, combined with different intracellular signalling domains (CD3ζ, CD28 and CD137) are trans- ferred to NK-92 cells via lentiviral vectors. Functionality of CAR-NK-92 cells are evaluated against the CD19- and CD19+ cell lines by analysis of degranulation and cytokine secretion.

## Results

Vector design comprising CD3ζ only increases nonspecific activity in terms of both de- granulation and TNFα secretion. While the CD28ζ-CD3ζ vector works efficiently in NK-92 cells, CD137ζ- CD3ζ vector does not seem to trigger high levels of antigen specific activity.

## Conclusion

CD3ζ only vector seems to increase unspecific activity due to its effect on expression of CD3ζ-coupled NK cell receptors. Our results provide valuable data for optimal CAR vector design in NK cells.

## Keywords

natural killer cells,immunotherapy,chimeric antigen receptor

## Acknowledgement

Thanks to Dr. Winfried S.Wels for CAR backbone plasmids.

# Abstract: Investigation of the relationship between aspartame and Swi6 in *Schizosaccharomyces pombe*

**Authors:** Beste BAYRAK, Merve YILMAZER, Bedia PALABIYIK

**Affiliations:** Istanbul University, Istanbul, Turkey

## Background/aim

Aspartame (APM) is an artificial sweetener used in many products since 1981. While some studies have found carcinogenic effects of APM, some others could not detect any negative effect. In this research, our aim is to shed light on molecular mechanism of APM effects in a simpler model organism *Schizosaccharomyces pombe*. In several cancer studies, it has been found that HP1 expression was downregulated. Thus for the evaluation, human HP1 (heterochromatin protein 1) family ortholog of Swi6 is selected. Swi6 is a telomere, centromere, and mating-type locus binding protein for the regulation of heterochromatin structure.

## Materials and methods

To verify if the effects of APM is related to Swi6, *S. pombe* parental and swi6Δ cultures were added 1 mg/mL APM every day for 7 days to mimic real life consumption habits, then analyzed with viability and quantitative real time polymerase chain reaction (qRT-PCR) methods.

## Results

We detected in qRT-PCR data that while on first day APM caused a decrease in the expression of swi6 in parental, expression levels for energy metabolism (*hxk2, fbp1, tup11, cyr1, cox4*) and stress response genes (*atf1, sod1, ctt1*) between parental and *swi6Δ* on first and seventh day differed significantly, depending on the effect of APM.

## Conclusion

According to the first day results, it has been seen that *S. pombe* parental strain has adapted to APM effects by activating the stress response pathway and *swi6Δ* did not present a meaningful response. Thus, we proposed that there may be a relationship between APM and Swi6. Nevertheless on the seventh day, the cumulative effects of APM showed that cells were in a trend of fermentation, which is an indication for carcinogenic activity.

## Keywords

cancer,Aspartame epigenetics,*Schizosaccharomyces pombe*,Swi6

## Acknowledgement

This study was funded by Scientific Research Project Coordination Unit of Istanbul University. Project number: FYL-2018-30854.

# Abstract: Association of single nucleotide polymorphisms (SNP)s in PLA2G2A gene with prostate cancer

**Authors:** Meltem Selen ONAL, Kaan OZTURK, Asif YILDIRIM, Nihan CELEBI-OLCUM, Dilek TELCI

**Affiliations:** 1 Yeditepe University, Istanbul, Turkey; 2 Istanbul Medeniyet University, Istanbul, Turkey

## Background/aim

Phospholipase A2s (PLA2)s are lipolytic enzymes hydrolyzing membrane phospholipids to form fatty acids and lysophospholipids. One of the key products is Arachidonic Acid (AA) that can be metabolized into prostaglandins and leukotrienes. sPLA2s have been associated with various cancers including prostate cancer. High level of sPLA2-IIA is correlated with high prostate tumor grade, tumor proliferation in primary site tumors. The expression of sPLA2-IIA both in mRNA and protein level increases more rapidly in metastatic tumors when compared to benign tumors. Furthermore, clinical studies on different single nucleotide polymorphism (SNP) of sPLA2- IIA indicated an association between the elevated serum levels of sPLA2-IIA in the cancer patients of afore mentioned diseases. This study aims to investigate the association of 5'UTR variant rs11573156 and the missense variants rs374105365 and rs965800220 on prostate cancer development.

## Materials and methods

Possible effects of missense variants were investigated through molecular dynamics simulations. For this purpose, mutated proteins were modelled in Cyrus Bench and simulations were run in Amber12 software. Results have been investigated in terms of H-bonding and surface electrostatic potential change. Genetic profile from a total of 267 blood samples (82 control and 184 PCa) was analyzed by polymerase chain reaction-restriction fragment length polymorphism (PCR-RFLP) method and Sanger sequencing.

## Results

Two missense variants, rs374105365 and rs965800220, show no effect on catalytic dyad interaction in terms of H-bond profile, while changes in amino acid side groups caused shift on surface electrostatics. The heterozygote allele of rs11573156 show a 2-fold decreased risk for PCA while no significant correlation was detected for other SNPs.

## Conclusion

Our results showed that although there is a strong association between heterozygous genotype of rs11573156 (p < 0.05) and prostate cancer, there is no association between other missense variants and prostate cancer.

## Keywords

Prostate cancer,phospholipase A2,single nucleotide polymorphism

# Abstract: Nutrient restriction induced cell death in Caco-2 colon cancer cells

**Authors:** Aliye Ezgi GÜLEÇ, Hepsen Hazal HÜSNÜGIL, Sreeparna BANERJEE

**Affiliations:** 1 Baskent University, Ankara, Turkey; 2 Middle East Technical University, Ankara, Turkey

## Background/aim

Colorectal cancer (CRC) is the third most common cancer worldwide. Despite therapeutic advances, low drug sensitivity is still an obstacle to successful chemotherapy. Recent studies have revealed the importance of nutrient restriction on the rewiring of metabolic networks of cancer cells. Induction of autophagy is one of the responses of cells to nutrient restriction. Given their fundamental role in development and disease, we have hypothesized that miRNAs may regulate genes that are involved in this rewiring. The aim of this study is to identify miRNAs that are involved in nutrient restriction mediated rewiring of metabolism in cancer cells.

## Materials and methods

We have subjected Caco-2 CRC cells to nutrient restriction by culturing these cells for 48h in a pre-optimized mix of low serum [1% fetal bovine serum (FBS) as opposed to 20% FBS], low glucose (0.1g/L as opposed to 1g/L) and low L-glutamine (0.2 mM as opposed to 2mM). Later, protein samples were collected from control and nutrient restricted cells. Then, au- tophagy and apoptosis induction was assessed with western blotting for the autophagy and apoptosis markers.

## Results

Under nutrient restriction, Caco-2 cells were observed to undergo autophagy, as indicated by enhanced ratio of LC3II/I, beclin-1 levels and p62 levels, and apoptosis, as indicated by decreased procaspase-3 levels.

## Conclusion

These results showed that starved Caco-2 cells underwent autophagy. This confirms the well-known paradigm that cells under the stress of starvation frequently undergo autophagy, a form of "self-eating" that allows the cell to eliminate organelles and proteins as well as other cargos in order to cope with the stress.

## Keywords

Colorectal cancer,nutrient starvation,autophagy

## Acknowledgement

TÜBITAK 118Z116

# Abstract: Comparison of autophagy in *Bombyx mori* midgut tissue under starvation condition and metamorphosis

**Authors:** Tugçe ERGIN, Ebru GONCU

**Affiliations:** Ege University, Izmir, Turkey

## Background/aim

Autophagy is a well-known physiological process which is mainly activated under starvation condition and act as a type of cell death under certain conditions. The aim of this study was to investigate the differences between autophagy processes during starvation conditions and metamorphic remodeling period of midgut of *Bombyx mori*.

## Materials and methods

*Bombyx mori* larvae were reared until 5^th^ larval stage. Half of the larvae were starved from the beginning of the 5^th^ larval stage. The other group was reared under normal feeding conditions. Midguts from both groups were dissected every 24 hours. Expressions of autophagy-related genes -ATG8 and ATG 12- were detected by qRT-PCR. Lysosomal hydrolytic enzyme acid phosphatase activity was measured spectrophotometrically as an autophagy marker. Midgut morphologies were evaluated with qRT-PCR and enzyme activity results.

## Results

The starved larvae survived until day 7 of 5^th^ larval stage. In the control group ATG8 expression gradually increased from day 0 to day 7 of 5^th^ instar but its expression was considerably low in starved insects especially after day 2 of 5^th^ instar. Similar to ATG8, ATG12 mRNA levels were found higher than starved insects. Acid phosphatase activities were not significantly different in both groups. Midgut morphologies were consistent with other results.

## Conclusion

Larval midgut is the main site for digestion. According to our results, autophagy was not stimulated in the midgut of starved larvae, conversely it was inhibited compared with the control group. These results suggested that autophagy is not involved in cell's survival during starvation. In addition a previous study showed that digestive enzyme synthesis activity of midgut cells precede under starvation condition. Therefore it is thought that cells meet the energy needs from the fat body in order to survive and maintain their functions.

## Keywords

Autophagy,*Bombyx mori*,midgut,starvation

# Abstract: Verification of novel targets of the Wnt/β-catenin pathway targets

**Authors:** Ayse Nur KAYABASI, Necla Birgül IYISON

**Affiliations:** Bogaziçi University, Istanbul, Turkey

## Background/aim

Wnt/β-catenin signaling pathway is an evolutionary conserved pathway in ver- tebrates which controls embryonic development and adult homeostasis. Having an important role in tissue homeostasis, aberrant activation of Wnt/β-catenin signaling is often linked to a wide va- riety of cancers. Mutations in target molecules of this pathway are identified to have tumorigenic effect. Previous studies confirmed BRI3 and MGAT1genes to be novel transcriptional targets of the Wnt/β-catenin pathway. Huh7 cells (hepatocellular cancer cell line) stably expressing either BRI3 or MGAT1 have greater proliferative and invasive capabilities compared to wildtype Huh7 cells, and when subcutaneously injected into NUDE/SCID mice resulted in larger tumor formation. In this study, we investigated BRI3 and MGAT1 as novel targets of the Wnt/β-catenin pathway and their involvement in hepatocarcinogenesis in vitro. We examined the RNA-Seq results of tumors derived from either BRI3 or MGAT1 overexpressing Huh7 cells compared to GFP overexpressing Huh7 cells and observed differential expressions of several genes. By selecting three genes for both BRI3 and MGAT1 based on their fold change and relevance to cancer, we proposed possible mechanisms of both BRI3 and MGAT1 contributions to tumorigenesis separately.

## Materials and methods

qRT-PCR and Western Blot analyses have been used to determine differential expressions of selected genes in either BRI3 or MGAT1 overexpressing Huh7 cells compared to control. Bioinformatic analysis using PRISM online tool has been carried out.

## Results

We verified differential expressions of selected genes for both BRI3 and MGAT1 separately and proposed a possible mechanism using both our data and previous studies.

## Conclusion

BRI3 and MGAT1 contribute to tumorigenesis by regulating the expression of some genes in Huh7 cells.

## Keywords

HCC,Wnt pathway,BRI3,MGAT1

## Acknowledgement

This project was supported by the funds of BAP project number 13123.

# Abstract: Interaction between heat-shock protein 90 (HSP90) and transglutaminase (TGase) in model plant *Brachypodium distachyon* under drought stress

**Authors:** Aylin KORKMAZ, Dilek TELCI, Bahar SOGUTMAZ OZDEMIR

**Affiliations:** Yeditepe University, Istanbul, Turkey

## Background/aim

Drought stress is one of the biggest and severe abiotic stress that cause reduction in crop productivity and yield. Plants continuously encounter stress stimuli due to their sessile structure and have to give quick responses to these stimuli. These stress response pathways depend on the action of heat-shock proteins (HSPs) by means of their intracellular chaperone role in protein- protein interactions by facilitating protein folding. HSPs are divided into 5 groups as HSP60, HSP70, HSP90, HSP100 and small heat shock proteins (sHSPs) and 1 to 6 % of the total protein in a cell contains HSP90. Free and bound formed polyamines (PAs) are naturally found molecules that can directly or indirectly affect HSP90 expression levels. Another protein that can interact with PAs is transglutaminase (TGase). The main roles of TGase are defined by catalyzation of protein cross- linking and post-translational protein modification including free PA incorporation. The objective of this study was to understand the HSP90 and TGase interaction under drought stress in a model plant. For this purpose, *Brachypodium distachyon* (Bd21 line) was used since it can reflect the physiological and molecular mechanisms in other monocot plants, especially economically important cereals.

## Materials and methods

*Brachypodium distachyon* plants were exposed to drought stress by water withholding for 12 days at their vegetative stage under greenhouse conditions. Proteins extracted from collected leaves were analyzed using western blot for HSP90 and TGase expression.

## Results

Western blots showed that HSP90 protein expression increased approximately 7-fold whereas TGase protein level decreased almost 0.23-fold under drought stress.

## Conclusion

The protein expression levels, together with known mechanisms and PA interactions under drought stress, have unraveled the possibility of an indirect interaction between HSP90 and TGase in *Brachypodium distachyon*. This study highlights the importance of molecular mechanisms behind drought stress.

## Keywords

Heat-shock Protein 90,Transglutaminase,*Brachypodium distachyon*,drought stress

# Abstract: Structure-activity relationship of 3-aryloxypyridyl-4*H*-1,2,4-triazoles as novel flexible benzodiazepine analogues

**Authors:** Latifeh NAVIDPOUR, Shayan SHEIKHI, Soraya SHAHHOSSEINI, Mona KHORAMJOUY

**Affiliations:** 1 Tehran University of Medical Sciences, Tehran , Iran; 2 Shahid Beheshti University of Medical Sciences, Tehran, Iran

## Background/aim

Benzodiazepines (BZDs) are among one of the most important drugs affecting central nervous system, clinically used as anxiolytic, sedative/hypnotic, muscle relaxant, and anticonvulsant agents. BZDs operate through binding to a specific domain of GABA_A_ receptor, a chloride ion channel, and modulate the action of GABA (g-amino butyric acid). In previous works, diverse non-rigid structures, preserving an aromatic or heteroaromatic A-ring (participating in p-p stacking interactions with aromatic amino acids of the receptor), a coplanar proton-accepting group at position 2 at a suitable distance from ring A, (interacts with histidine residue of the receptor) and an out-of-plane phenyl ring, were reported to have considerable anticonvulsant activity. In most benzodiazepines, the presence of electron withdrawing substituents at ortho position of out-of-plane phenyl ring improves the activity, while in meta and para position eliminate the activity. Docking studies have shown the possible different mode of interaction for the non-rigid analogs with the binding site. Therefore, it is interesting to investigate if meta or paras-substituted analogs preserve affinity on benzodiazepine receptors.

## Materials and methods

The new non-rigid 3-aryloxypyridyl-4*H*-1,2,4-triazoles were synthesized and their binding affinity to GABA_A_/BZD Receptor complex was evaluated by their ability to displace [^3^H]-flumazenil (Ro15-1788) from its specific binding in rat cortical membrane tissue.

## Results

The concentration of the tested compounds (non-radioactive ligands) that inhibits the binding of [^3^H]-flumazenil by 50% is considered as IC50 values. Interestingly, the meta and para of non-rigid analogs, in contrary to classic benzodiazepines were shown significant affinity to BZD receptors.

## Conclusion

Recent introduced flexible BZDs show very high affinity to GABA_A_/BZD Receptor complex, but possible different mode of interaction requires structure-activity relationship studies.

## Keywords

1,2,4-triazole,flexible benzodiazepines,GABA_A_/BZD receptor binding assay

# Abstract: Screening of trisomy 21 in pregnant Turkish women via non-invasive prenatal test (NIPT)

**Authors:** Gizem ALKURT, Sezin CANBEK, Murat MUHCU, Ebru A. DAVUTOGLU, Levent DOGANAY

**Affiliations:** 1 Istanbul Technical University, Istanbul, Turkey; 2 Health Science University, Istanbul, Turkey

## Background/aim

Non-invasive prenatal test (NIPT) is a screening method for the detection of fetal aneuploidies via obtaining cell free fetal DNA in the prepherial blood of pregnant woman. Trisomies of chromosome 13 (Patau Syndrome), 18 (Edward Syndrome) and 21 (Down Syndrome) is determi- ned by NIPT. Here results for trisomy 21 are presented.

## Materials and methods

This test was applied to 440 women with pregnancy who were referred to Perinatology Clinic due to having a risk factor for any of the trisomies in between April 2018 and August 2019. Patient selection criteria were, maternal age above 40 ages, having high double marker test results (1:100-1:1000) or having abnormal finding in sonographic examination. The whole blood was taken after 12 weeks pregnant. cfDNAs were isolated under pressure via QIAVac and library was prepared with 12 cfDNA samples per run on MiSeq. The analysis was done on Clarigo Reporter, fetal fraction value and trisomy status were determined.

## Results

Trisomy 21 was detected in 4 (0.9%) of 440 patients. Of them, in three patients double mar- ker test results were high risk and cardiac hyperechogenic focus was observed in the fourth patient. All positive results were confirmed by amniocentesis. Moreover, 78 of 440 samples were repeated due to low fetal fraction, 64 of them had a negative result but in 14 patients (3%) NIPT failed to give a conclusive result.

## Conclusion

NIPT is a highly efficient screening test, with very low false negative result. Having high risk ratios in double marker test often yields to invasive procedures like amniocentesis but NIPT efficiently lowers the need for invasive diagnostic approaches.

## Keywords

NIPT,trisomy 21,NGS

# Abstract: Apoptosis and autophagy signaling repress the transcription of GPD1 and CYC1 gene

**Authors:** Eren TANIK, Ceyda ÇOLAKOGLU, Osman Alp ÖZTOP, Sezai TÜRKEL

**Affiliations:** Bursa Uludag University, Bursa, Turkey

## Background/aim

Autophagy and apoptosis are two basic cellular processes that are activated under distinct cellular growth conditions. NAD-dependent glycerol-3-phosphate dehydrogenase, which is encoded by the GPD1 gene, is essential for the regulation of redox balance. Cytochrome c, isoform 1, encoded by the CYC1 gene, locates within intermembrane space functions as the electron carrier for mitochondrial functions. Cyc1 also has a function in the initial stage of apoptosis. The aims of this study are to investigate how apoptosis and autophagy signaling affects the transcription of GPD1 and CYC1 genes. We have also tested the involvement of protein kinase Hog1/MAPK11 in regulation of these genes during apoptosis and autophagy signaling.

## Materials and methods

To quantitate the expression levels of GPD1 and CYC1 genes, we have used the LacZ gene fusions of GPD1 and CYC1 genes that contain the entire promoter regions of these genes. To activate apoptosis, yeast cells grown in 60 mM acetic acid for 5 h. Autophagy signaling is activated by blocking the TOR pathway with 8 mM caffeine.

## Results

Activation of apoptosis resulted in 4 to 8-fold repression of GPD1 and CYC1 genes tran- scription, both under fermentative and respirative growth conditions. On the other hand, activation of autophagy differentially affects the expression of GPD1 and CYC1 genes. While GPD1 transcription is activated by 25% upon autophagy signal triggered by caffeine incubation, transcription CYC1 is repressed by 2-fold.

## Conclusion

The activation of apoptosis rapidly represses the transcription of the GPD1 and CYC1 gene. It seems that apoptosis and autophagy signaling might affect redox balance within the cells through the regulation of GPD1 transcription.

## Keywords

Apoptosis,autophagy,redox balance,cytochrome C1,yeast

# Abstract: A preliminary study for soil probiotic: Evaluation of siderophorogenic bacteria

**Authors:** Kübra ERKAN TÜRKMEN, Hikmet KATIRCIOGLU

**Affiliations:** 1 Hacettepe University, Ankara, Turkey; 2 Karamanoglu Mehmetbey University, Karaman, Turkey; 3 Gazi University, Ankara, Turkey

## Background/aim

Probiotics are defined as health benefit microorganisms for the host.Probiotic ac- tivity is related to the limitation of pathogens and balances microbial flora in human.Besides, probiot- ics promote many mechanisms of the plant which are effect plant growth and production of sidero- phore. All organisms have many specific mechanisms to transport the iron from their environment into cells. One of the processes for iron transport is siderophore. It is defined as low molecular weight and specific affinity to chelate iron. Polysaccharides are defined as natural compounds that have anti-inflammatory activity. Many bacteria are able to produce different variety of polysaccharides. Exopolysaccharide (EPS) is one of the kinds of polysaccharide produced by bacteria that secreted into the environment. This study was aimed to determine the quality and quantity of Siderophore and EPS production from bacteria.

## Materials and methods

All bacteria used in this experiment isolated from soil. Soil samples were taken pesticide-free agricultural land. Each soil sample was diluted with sterile saline solution. For each serial dilution of soil samples,50 μl volume sample was taken from dilution tube and inoculated on Chrome Azurol S (CAS) agar. Also, EPS production capacity of isolates was determined by phenol sulphuric acid method. Identification of siderophore producing isolateswere used 16srRNA analysis.

## Results

In 22 isolates,3 isolates were determined the highest siderophore producing capacity. One of these isolates had high siderophore and eps producing activity. As Regards to16srRNA analysis results, it was determined as *Bacillus subtilis*.

## Conclusion

Last years, soil probiotics had been remarkable area for plant protection. There have searched new methods for biocontrol strategies against plant-pathogens. Siderophoregenic probiotic bacteria would be considered as novel aspects.

## Keywords

soil probiotic,siderophore,EPS

## Acknowledgement

The authors special thank to Scientific Research Projects Unit of GAZI UNI- VERSITY (Project No:04/2017-03) for supporting this study.

# Abstract: Transcriptome profiling of LAP1B-deficient myoconverted fibroblasts reveals pathways underlying their differentiation defect.

**Authors:** Gülsüm KAYMAN KÜREKÇI, Aybar C. ACAR, Pervin R. DINÇER

**Affiliations:** 1 Hacettepe University, Ankara, Turkey; 2 Middle East Technical University, Ankara, Turkey

## Background/aim

Lamina-associated polypeptide 1 (LAP1) is a ubiquitously expressed integral in- ner nuclear membrane protein. In human, a frameshift mutation leading to the specific loss of LAP1B isoform causes muscular dystrophy. Our aim is to elucidate the molecular pathways implicated in the muscle phenotype caused by LAP1B deficiency.

## Materials and methods

We transdifferentiated LAP1B-mutant fibroblasts isolated from patient into myoblasts by using doxycycline-inducible MyoD gene transfer. Fusion and differentiation potentials were monitored for a period of 8 days after switching to differentiation medium. RNA sequencing was performed with total RNA isolated from control and mutant cells at four differentiation time- points. Enrichr tool and STRING database were used for gene enrichment analyses.

## Results

Myogenic differentiation and fusion indexes were dramatically lower in mutant cells com- pared to control cells. Transcriptome analyses revealed a prominent number of downregulated genes in mutant cells compared to control at each differentiation day. Comparison of transcriptome through- out differentiation showed that a subset of genes upregulated in control cells are downregulated or unchanged in mutant cells. Differentially expressed genes identified in regression analysis for time- course expression were mainly associated with extracellular matrix organization, cell cycle and skel- etal muscle contraction. While differentially expressed genes unique to control cells were correlated with myogenic differentiation, genes unique to mutant cells were enriched for translation and protein targeting to ER.

## Conclusion

Results suggest that loss of LAP1B is associated with a loss of the activation of a subset of genes involved in myogenic differentiation. Transcriptome data elucidated pathways that fail to be reprogrammed during myogenic differentiation in mutant cells and indicate a defect in cell cycle exit.

## Keywords

myogenic differentiation,LAP1,transcriptome

## Acknowledgement

This study was funded by the Scientific and Technological Research Council of Turkey, Project number 116S307 to P. Dinçer.

# Abstract: Functional characterization of Joubert syndrome associated CEP41

**Authors:** Mustafa S. PIR, Sebiha CEVIK, Oktay I. KAPLAN

**Affiliations:** Abdullah Gul University, Kayseri, Turkey

## Background/aim

Ciliopathies are typically a group of genetic disorders arising from mutations in genes encoding proteins that particularly localize to cilia and/or the basal body of cilia or the proximal region of cilia (transition zone). Joubert Syndrome (JS) is a ciliopathy disease typically characterized by hypoplasia of the cerebellar vermis, ataxia and various cognitive impairments. Mutations in 35 distinct genes, including *CEP41*, have been linked to Joubert Syndrome. *CEP41* encodes a microtubule-associated component of the ciliary axoneme. However, the mechanisms by which *CEP41* controls cilia biogenesis remains poorly defined. We have therefore investigated the function of *CEP41* in *Caenorhabditis elegans*, where cilia are located at the end of long dendrites of sensory neurons.

## Materials and methods

We first generated a null mutant in C.elegans using the CRISPR / Cas9 technique. We also produced a transgenic strain expressing GFP tagged *CEP41* construct. We then used standard genetic crossing techniques coupled with the fluorescence microscopy to assess the cilia structure of *CEP41* null mutant.

## Results

Consistent with its mammalian localization, we found that GFP tagged *CEP41* localizes to cilia in *C. elegans*. Furthermore, we noticed that *CEP41* is restricted to the middle segment of the cilia, excluded from the distal regions of the cilia.

## Conclusion

After confirming the cilia localization of *CEP41* in *C. elegans*, we are currently assess- ing the cilia structure of *CEP41* mutant and we will present recent findings in the meeting.

## Keywords

Joubert Syndrome,ciliopathy,*CEP41*,CRISPR/Cas9,*C. elegans*

## Acknowledgement

This work is supported by the Scientific and Technological Research Council of Turkey (TÜBITAK), Grant No: SBAG- 118S552

# Abstract: In vitro serotonin and antioxidants supplementation increase human sperm motility

**Authors:** Elnaz MOSHFEGHI, Yasemin YILMAZER, Necati FINDIKLI

**Affiliations:** 1 Yildiz Technical University, Istanbul, Turkey; 2 Bogazici University, Istanbul, Turkey; 3 Bahceci Health Group, Istanbul, Turkey

## Background/aim

Infertility is a problem affecting nearly 15% of couples in the World. Recent re- searches indicate that about 50 % of infertility cases are exclusively caused by a male factor. Besides concentration and morphology, decrease in motility is directly associated with infertility. We aimed to evaluate the possible beneficial effect of serotonin (5-HT), selenium (Se), Zinc (Zn), vitamin D and E supplementation on sperm motility.

## Materials and methods

Semen samples were obtained from 120 patients between the ages of 28 and 40 years old with 3-5 days of sexual abstinence. Following liquefaction, basal semen analysis was performed according to the WHO manual criteria. Samples were prepared and incubated in Hu- man Tubal Fluid (HTF) media. Containing different concentrations of 5-HT, Se, Zn, vitamin D and E at 37 C for 75 min. At the end of the incubation, sperm concentration and motility were evaluated.

## Results

Progressive motility is an important condition for achieving ovum fertilization in the female reproductive tract during the capacitation. The percentage of fast progressive (+4), slow progressive (+3), non-progressive (+2) and immotile (+1) sperm cells were calculated for different concentrations of each supplement. After 75 min of incubation, there was a significant increase of fast progressive motility compared with control groups. While the mean +4 sperm motility was % 12.10 in control groups, it was increased to % 21.28 in the group of 200 μM 5-HT. Fast progressive sperm motility has also been observed to increase in 2 μg Se, 10 μg Zn, 100 nM vitamin D and 2 mmol vitamin E concentrations.

## Conclusion

Finally, incubation of human spermatozoa with 5-HT, Se, Zn, Vitamin D and E has a beneficial effect on sperm motility. These molecules in sperm preparation can have a potential to increase the outcome of assisted reproduction technologies.

## Keywords

infertility,sperm motility,serotonin

# Abstract: Biotechnological production of an allergenic lipid transfer protein from *Morus alba* pollen

**Authors:** Narmin AGHALAROVA, Narmin MIRZAMAMMADLI, Yunus AKSÜT, Nazli ARDA

**Affiliations:** Istanbul University, Istanbul, Turkey

## Background/aim

Plant lipid transfer proteins (LTPs) assemble a family of small cationic proteins with an ability to bind and transport lipids as well as participate in various physiological processes including defense against phytopathogens. They also form one of the most clinically relevant classes of plant allergens. However, the allergenic properties of LTP in *Morus alba* (white mulberry) have not been studied. This study aimed to produce LTP found in *M. alba* pollens using recombinant DNA technology.

## Materials and methods

After total RNA isolation from *M. alba* pollens, a cDNA library was constructed. The sequence of the LTP gene amplified by PCR from this cDNA library was determined by sequencing and verified by the BLAST analysis. Then PCR product was cloned into the pQE- 2 vector. E.coli BL21 (DE3) cells were transformed with the vector for the recombinant protein synthesis. The recombinant allergen was isolated from transformants, and purified by Ni2+ affinity chromatography. rLTP was detected on SDS-PAGE gel and PVDF membrane by Coomassie blue staining and Western blotting, respectively.

## Results

Recombinant LTP was isolated for the first time from *M. alba* pollen. We identified an approximately 12-kDa protein as an allergen.

## Conclusion

Since LTP allergens from pollen and many foods are known to have a high prevalence in allergic patients, rLTP produced in this study may be used for diagnostic and therapeutic purposes in the future, following further clinical studies.

## Keywords

Pollen allergy,*Morus alba*,lipid transfer protein,recombinant allergen.

## Acknowledgement

This study was funded by Scientific Research Projects Coordination Unit of Istanbul University, Project Number:33524.

# Abstract: Recombinant production of allergenic pectate lyase from cypress pollens

**Authors:** Narmin MIRZAMAMMADLI, Narmin AGHALAROVA, Yunus AKSÜT, Nazli ARDA

**Affiliations:** Istanbul University, Istanbul, Turkey

## Background/aim

Allergic rhinitis is a chronic disease becoming a major health problem, especially in metropolitan cities, mainly due to pollens. The *Cupressus sempervirens* (cypress) pollen is one of the most important causes of pollinosis, and it contains pectate lyase (Cup s 1) as a major allergen of approx. 40 kDa. The aim of this study was recombinant production of Cup s 1 in *Escherichia coli* for further use in the diagnosis and treatment of cypress pollen allergy.

## Materials and methods

Total RNA was isolated from *C. sempervirens* pollens (gathered in Istanbul), complementary DNA (cDNA) was synthesized, and then cDNA was used to amplify the *Cup s 1* gene using PCR technique. The sequence of the *Cup s 1* gene amplified by PCR was determined and verified by the BLAST. PCR product was then cloned into the pQE-2 vector. E. coli BL21 (DE3) cells were transformed with the vector for the recombinant protein synthesis. Recombinant protein was isolated from transformant cells, and purified by Ni^2+^ affinity chromatography. Subsequent to SDS- PAGE, Coomassie staining as well as Western blotting were used for detecting recombinant protein band.

## Results

Recombinant *Cup s 1* was successfully produced in E. coli and purified. Corresponding 40 kDa band was detected in the transformant cells, but not in the host cells following purification.

## Conclusion

Obtained recombinant allergen may be used in the diagnosis and/or treatment of cypress allergy after clinical trials in the future. This study is expected to provide a basis to similar attempts to improve the sensitivity and reliability of diagnosis of allergic diseases, and success of treatment in patients living in Istanbul.

## Keywords

Pollen,*Cupressus sempervirens*,Recombinant allergen,*Cup s 1*

## Acknowledgement

This study was funded by Scientific Research Projects Coordination Unit of Istanbul University, Project Number: 33555.

# Abstract: Immunoproteomic Analysis of Acinetobacter baumannii Surface and Secreted Proteins

**Authors:** Aysegul MURAT, Serife AYAZ-GUNER, Mustafa Burak ACAR, Eda MERT GÖKDUMAN, Gokcen DINC, Aysegul ULU KILIC, Mehmet DOGANAY, Servet OZCAN

**Affiliations:** 1 Erciyes University, Kayseri, Turkey; 2 Ege University, Izmir, Turkey; 3 Abdullah Gul University, Kayseri, Turkey

## Background/aim

Multidrug resistant Acinetobacter baumannii (MDRAB) is one of the most im- portant biothreat agents for the patients especially with warfare and intensive care units. Nowadays, treatment choices are very limited due to resistance of antimicrobial agents and unavailability of vac- cines. This study was aimed to investigate the potential vaccine candidates for A. baumannii infection.

## Materials and methods

A. baumannii standard strains ATCC 17978 (sensitive), ATCC BAA-1710 (multidrugresistant) and two local strain were used in the study. Protein samples of outer-membrane and secretome were collected. A. baumannii proteins pulled down by using patient IgG. Immuno- precitated proteins were identified by using LC-MS/MS and bioinformatic analyses were carried out by Immune Epitope Data-Base.

## Results

Immuno-reacted 280 proteins were identified via patients' sera. 38 of these proteins that were only expressed in resistant strain. By using bioinformatics tools, 9 out of 38 candidates have been selected according to criteria of solubility, transmembrane property and B lymphocyte-specific epitopic region characteristics.

## Conclusion

In order to use these antigenic proteins of A. baumannii as vaccine candidates or diag- nostic tools, these 9 proteins need to be further characterized and tested for protective functions in animal model.

## Keywords

immune-proteomics,A. baumannii,vaccine candidate

## Acknowledgement

This research supported by the Scientific and Technological Research Council of Turkey (TÜBITAK project no. 114S571).

# Abstract: Methylation and expression levels of *GABRB2* gene in oral squamous cell carcinoma.

**Authors:** Sena SEN, Can DORUK, Murat ULUSAN, Semra DEMOKAN

**Affiliations:** Istanbul University, Istanbul, Turkey

## Background/aim

Oral cavity cancers are one of the most common malignancies in the world and the majority (90%) of these cancers is caused by oral squamous cell carcinomas (OSCC). Etiological factors such as smoking and alcohol use, ethnicity, age and geographical region play a role in the development of OSCC. Prof.Dr. Semra Demokan and her colleagues observed that expression of *GABRB2* gene was reduced by promoter methylation in OSCC patients' tumor tissues performed by methylation and gene expression arrays reported in her project "TUBITAK-SBAG-114S497". *Gamma-Aminobutyric Acid Receptor, Beta-2* (*GABRB2*) gene encodes the gamma-aminobutyric acid (GABA) receptor and is related to signal transmission in neurons. In our study, the methylation-based expression loss of *GABRB2* gene was studied in a larger group of patients for further validation. In our study (IU-BAP-TYL-2017-27208) We investigated that whether *GABRB2* gene could be a potential biomarker for prognosis and early diagnosis of OSCC.

## Materials and methods

The methylation and expression status of *GABRB2* gene were analyzed in tumor and corresponding-normal tissue and serum samples of 50 patients and 10 healthy individuals' normal tissue and serum samples by the QMSP and QRT-PCR, respectively. The obtained results were compared with clinical parameters and evaluated by statistical analysis.

## Results

*GABRB2* gene hypermethylation was observed in 14% (7/50) of patients, and decreased expression levels were found in 44% (22/50) of patients. The methylation-dependent expression loss of *GABRB2* was observed in 3 of 7 patients having methylated promoter region. There was a significant decrease in the expression level in all of tumor of the floor of the mouth.

## Conclusion

Our study suggests that there was an association between methylation-based expression loss of *GABRB2* and OSCC' subgroups.

## Keywords

methylation,gene expression,oral squamous cell carcinoma,epigenetic biomarker,*GABRB2* gene

# Abstract: Death domain protein has dual roles in feedback regulation of Wnt/β-catenin signaling and apoptosis

**Authors:** Özgün ÖZALP, Özge ÇARK, Yagmuz AZBAZDAR, Günes ÖZHAN

**Affiliations:** 1 Dokuz Eylül University, Izmir, Turkey; 2 Izmir Biomedicine and Genome Center, Izmir, Turkey

## Background/aim

Wnt/β-catenin signaling regulates proliferation, migration and tissue patterning during embryogenesis and tissue homeostasis. Thus, aberrations in Wnt signaling can cause devel- opmental defects and several types of cancers. Although many components of the Wnt/β-catenin pathway have been characterized, many questions related to the regulation of signaling remain un- answered. Therefore, we aim to unravel the role of a novel neurotrophin associated death domain gene, which we identified as a Wnt/β-catenin target gene, in regulation of Wnt/β-catenin signaling and apoptosis.

## Materials and methods

To understand whether the death receptor controls Wnt/β-catenin signaling, we performed dual luciferase reporter assay in HEK293T and HUH7 cells. Next, we performed An- nexin V-FITC staining by flow cytometry method in same cells to test apoptosis. Finally, to unravel the role of the death receptor in development, capped sense mRNA of the gene was microinjected into wild type and Tg(*hsp70l:wnt8a-GFPw34*) zebrafish embryos. Apoptosis rates were assayed in embryos by whole-mount immunofluorescence using anti-activated Caspase 3.

## Results

Results showed that the death receptor is both a target and a negative regulator of Wnt/β-catenin signaling. Moreover, the death receptor could induce apoptosis in HEK293T and HUH7 cells. Intriguingly, while overexpression of the death receptor or activation of Wnt signaling caused a mild increase in apoptosis rate in zebrafish embryos, overexpression of the death receptor in embryos where Wnt signaling is activated strongly enhanced apoptosis, suggesting that the death receptor synergizes with Wnt/β-catenin signaling in promoting apoptosis.

## Conclusion

Data show that the death receptor gene acts synergistically with Wnt/β-catenin signaling to enhance apoptosis in embryonic development. As pathway modulators that tightly fine-tune signal- ing activation, novel death domain protein of Wnt/β-catenin signaling will significantly contribute to discovery of novel drugs targeting the pathway.

## Keywords

zebrafish,apoptosis,neurotrophin,receptor,Wnt/β-catenin

## Acknowledgement

This project is supported by TÜBITAK (Project no:217Z123).

# Abstract: Identification of Cas9 ubiquitylation

**Authors:** Yusuf Tunahan ABACI, Arda Baran ÇELEN, Umut SAHIN

**Affiliations:** Bogaziçi University, Istanbul, Turkey

## Background/aim

The CRISPR/Cas9 system offers a simple method for genome engineering by utilizing the ability of the bacterial Cas9 enzyme to cleave any desired genomic region under the guidance of a complementary RNA molecule. Although this is a versatile and precise tool for gene editing, there is a lack of knowledge regarding its behavior and regulation in eukaryotic systems. In this study, we aim to elucidate the mechanisms of Cas9 regulation in eukaryotic systems with the specific goal of investigating the potential post-translational modifications (PTMs) on this protein.

## Materials and methods

Several cell biology-based techniques were exploited to elucidate Cas9 ubiquitylation. These techniques are His-pulldown, immunoprecipitation (IP) and Proximity Ligation Assay (PLA). Cas9 and His-Ubiquitin constructs were co-transfected to HEK293T cells for both His-pulldown and IP assays. Ubiquitin pool was pulled down and Cas9 blot was performed in His- pulldown assay, whereas Cas9 was pulled down and ubiquitin blot was performed in IP assays. PLA was performed using Cas9-HK2 cells that are stably expresses Cas9 protein. Primary antibodies against Cas9 and endogenous ubiquitin was used. In order to map ubiquitin modified lysines on Cas9 proteins we prepared samples for LC-MS/MS and sent them to EMBL for analysis.

## Results

All cell biology-based techniques (His-pulldown, IP and PLA) showed strong ubiquitylation on Cas9 protein. Several lysines were mapped to be ubiquitylated by mass spectrometry.

## Conclusion

We expect that these results will lead to a better understanding of the eukaryotic post- translational regulation of Cas9. Our hope is that uncovering the functional implications of these PTMs will contribute to the development of safer therapies and improve the current CRISPR-based applications.

## Keywords

CRISPR-Cas9,PTM,ubiquitylation

## Acknowledgement

European Molecular Biology Organization (EMBO)

# Abstract: Elucidating the putative function of NLRP7 in oncogenesis

**Authors:** Gizem Olay ARTIK, Aybüke GARIPCAN, Özen KAYA, Nesrin ÖZÖREN

**Affiliations:** Bogaziçi University, Istanbul, Turkey

## Background/aim

NLRP7 is a cytosolic protein involved in activation of proinflammatory caspases by participating in inflammasome oligomerization. Mutations of NLRP7 cause Hydatidiform Mole disease, characterized by excessive trophoblast proliferation and lack of embryo development. It is proposed that it may play an oncogenic role in endometrial cancer and testicular seminoma. How- ever, the mechanism(s) behind NLRP7's effect on oncogenesis is currently unknown. Xenograft ex- periments were performed in mice with stably NLRP7-expressing Hec1a endometrial cancer cells. NLRP7 overexpressing cells enhanced tumor growth when compared to control cells. Putative in- teractors were determined through LC-MS analysis and the outcomes focused on the Ras pathway. Several partner proteins were verified with co-IP experiments. Our initial results indicate that NLRP7 might be an effector of two Ras-related GTPases and it may interfere with the DNA damage response via ATM-Brat1-Chk1 axis. We aim to elucidate the mechanisms affected by NLRP7 in cancer forma- tion.

## Materials and methods

Xenograft experiments in SCID mice were performed to learn whether NLRP7 upregulation may trigger tumorigenesis. LC-MS gave us an information about what would be its interaction partners. Crispr-Cas9 system was used to knock out NLRP7. Co-IP experiments showed direct interaction between NLRP7 and putative interactors. Western Blotting is used to ob- serve changes in levels of the key oncoproteins and tumor suppressors in the presence or absence of NLRP7. Phenotypic assays are currently being performed.

## Results

According to xenograft experiment Hec1a cells overexpressing NLRP7 induces bigger tu- mour formation in SCID mice. NLRP7 is directly interacting with two Ras-related proteins, RalB and Rab5, and one DNA damage sensing protein, BRAT1 in endometrial cancer cells, Hec1a.

## Conclusion

NLRP7 enhances tumor formation through an unknown mechanism. We are proposing that NLRP7 is affecting oncogenesis via the Ras pathway and/or DNA-damage repair mechanisms.

## Keywords

NLRP7,endometrial cancer,Ras pathway,DNA damage repair

## Acknowledgement

TUBITAK 112S115

# Abstract: The significance of family study with NGS analysis: Interpretation of novel de novo mutations

**Authors:** Yasemin KENDIR DEMIRKOL, Levent DOGANAY

**Affiliations:** Health Sciences University, Istanbul, Turkey

## Background/aim

The arrival of next-generation sequencing (NGS) technologies coupled with advanced bioinformatics has changed the face of genetic diagnosis by offering faster, more affordable, and higher resolution testing options. The interpretation of variants of data obtained by NGS analysis is performed according to American College of Medical Genetics (ACMG) 2015 criteria. In this report, we present 3 cases whose NGS analysis revealed novel variants and those were classified as variant uncertain significance according to ACGM. However, family segregation study revealed that these variants were de novo mutation which were reclassified as pathogenic.

## Materials and methods

Case 1: 28 years old female has disproportionate short stature, facial dysmorphic features. Her X ray showed platyspondyly, kyphosis, scoliosis, flattened epiphyses. NGS was performed with skeletal dysplasia panel. Case 2: Six month old girl referred with growth and development delay. On physical examination his weight was 4250 (-3.8 SDS), her occipito-frontal diameter 38.5cm (-2.8 SDS). She has dysmorphic feature, hypotonic, joint laxity and congenital cataract. Her superficial veins were prominent. NGS was performed collagenopathy panel. Case 3: Eight years old girl was referred with frequent infection, skin rash, abscess on the scalp. NGS was performed with immunodeficiency panel.

## Results

Anovel heterozygous variant in case 1 in *COL2A1* gene (c.2006G>C), in case 2 in *ALDH18A1* gene (c.377G>A) and in case 3 in STAT3 gene (c.1861 T>G) were detected and segregation within the family showed that the mutations were de novo.

## Conclusion

Clinical evaluation of the patient is essential during generation of a medical report after an NGS analysis. Especially in cases with dominant heredity, segregation study in a family might reveal de novo mutations and this will help reclassifying the variant as pathogenic mutation especially when clinical findings suggest a mutation in that particular gene.

## Keywords

Next generation sequencing,de novo mutations,clinical evaluation

# Abstract: Investigation of possible therapeutic effects of γ-tocotrienol combined therapy on HepG2 cancer cell line

**Authors:** Akin SEVINÇ, Tugçe Nur ERALP

**Affiliations:** 1 Altinbas University, Istanbul, Turkey; 2 Yildiz Technical University, Istanbul, Turkey

## Background/aim

Hepatocellular carcinoma (HCC) is sixth in terms of incidence and second in terms of cancer-related mortality. Owing to its great burden on public health, development of therapeutic strategies for its treatment is very important. Effectiveness of doxorubicin (DOX) in clinical use does not reach desired levels due to side effects and resistance to the drug. For this reason, it is imperative to develop novel combination therapy strategies to increase the effectiveness of Dox. In recent studies, it has been shown that vitamin E group molecules, such as tocopherol and tocotrienols have potential therapeutic potential as adjuvant molecules. In liver cancer cells, γ-tocotrienol (GT3) has been shown to trigger apoptosis and restore sensitivity to chemotherapeutic chemicals. Evidence on the interplay of these effects with the biochemistry of liver cells is controversial. Therefore, we aimed to investigate the possible therapeutic effects of GT3 on liver cancer cell line using molecular biological techniques.

## Materials and methods

In order to investigate the effects of combination therapy on growth characteristics of HepG2 cells, we utilized MTT assay. Combination index was calculated by CompuSyn and impact of single versus combined drug application was investigated using western blot analysis.

## Results

IC50 for GT3 and Dox were calculated as 50 μM and 0.5 μM, whereas in the combination therapy these levels were 50 μM and 0.25 μM, respectively. In immunoblotting experiments, the amount of cleaved PARP (cPARP), active Caspase-3 and -7 increased with Dox application, whereas total PARP (tPARP) amount decreased in combination therapy. Similarly, BCL-XL, Bcl2, MCl1, phosphorylated Bcl2, NFKB, pAkt and pErk amounts decreased with Dox application.

## Conclusion

Our experiments have shown that GT3 combination therapy leads to an increase in the rate of apoptosis compared to Dox administration by altering the internal apoptotic pathways.

## Keywords

Hepatocellular carcinoma,γ-tocotrienol,doxorubicin,combination therapy

# Abstract: The gene mutations detected at our center decreased bone mineral density

**Authors:** Özlem YILDIRIM, Ebru ARSLAN, Hamza POLAT, Ceren ALAVANDA, Ipek YILMAZ, Pinar ATA, Ahmet ARMAN

**Affiliations:** Marmara University, Istanbul, Turkey

## Background/aim

Osteoporosis is a health problem mainly affecting older population which has a genetic background and might be preventable if detected earlier. Our aim is to search the effect of pathogenic mutation types at the patient group with reduced bone mineral density (BMD).

## Materials and methods

We had analyzed twenty six patients who admitted to Marmara University School of Medicine Medical Genetics Department between January 2018 to September 2019 with reduced BMD and pathological fractures. At the outpatient clinics the clinical/phenotypic findings were recorded and patients were analyzed with osteoporosis Next Generation Sequencing (NGS) gene panel covering IFITM5, PPIB, PLOD2, DSPP, CALCR, CRTAP, LEPRE1-P3H1, CTSK, TRAF6, SERPINF1, SERPINH1, FKBP10, ANO5, DOCK9, COL1A1, COL1A2, BMP1, LRP5,ALPL, COL2A1, VDR BMP2 genes. Also for those patients with a pathogenic mutation parental analysis were done.

## Results

There were 10 females and 16 males with age of 2 months to 47 years. We have detected 4 patients with SERPINH1 gene mutations, 5 patients with FKBP10 gene mutations, 5 patients with COL1A1 gene mutations, 2 patients with COL1A2 gene mutations, 3 patients with BMP1 mutations, 2 patients with PLOD2 gene mutations and 5 patients with POLG, C7, DOCK9, DSPP, ANOS and EXT1 genes mutations respectively.

## Conclusion

Our panel is effective for those patients with decreased BMD. Genetic analysis with osteoporosis gene panel is not a screening but rather an important diagnostic tool.

## Keywords

Reduced Bone Mineral Density,Next Generation Sequencing,Diagnostic Tool

# Abstract: Early onset Tay-Sachs disease mouse model shows altered autophagic pathway

**Authors:** Tugçe SENGÜL, Melike CAN, Nurselin ATES, Seçil AKYILDIZ DEMIR, Volkan SEYRANTEPE

**Affiliations:** Izmir Institute of Technology, Izmir, Turkey

## Background/aim

Tay-Sachs disease is one of the lysosomal storage disorder (LSD) caused by mutation in Hexa gene, encoding for the α subunit of lysosomal β-hexosaminidase A (HEXA), responsible for degradation of GM2. HEXA gene deficiency affects the central neurvous system owing to GM2 accumulation in lysosomes which causes neuronal death and progressive neurodegeneration in patients. Recently generated *Hexa-/-Neu3-/-* knockout mouse mimics neuropathological and clinical abnormalities of the classical early onset Tay-Sachs disease. As lysosomes have critical role in autophagy completion, many LSD show defect in autophagic flux and accumulation of autophagic substrates. In this study, we aimed whether accumulated GM2 causes autophagic flux alteration in different brain regions and fibroblasts from early onset Tay-Sachs disease mice model.

## Materials and methods

To understand autophagic flux alteration, cortex, cerebellum, thalamus and hippocampus region and fibroblast of *WT, Hexa-/-, Neu3-/-* and *Hexa-/-Neu3-/-* were studied. mRNA expression level of Atg7, Atg9, p62 and Beclin-1 were determined by RT-PCR analysis. Protein levels of LC3-I, LC3-II, p62 and Beclin-1 were measured by Western blotting. Intracellular localizations of LAMP1, LC3 and p62 were analyzed by immunohistochemical analysis.

## Results

Alterations of autophagic flux markers were detected in four brain region of early-onset Tay- Sachs disease mouse model. In particular, cerebellum and thalamus region of *Hexa-/-Neu3-/-* showed increased accumulation of autophagosomes and autophagic substrates. Interestingly, we observed that rapamycine induction in fibroblast from early-onset Tay-Sachs disease mouse model boosts the completion of autophagy flux.

## Conclusion

In this study we conclude that there is a clear impairment in the fusion of autophagosomes with lysosomes which results in accumulated autophagosomes and block of autophagic pathway in early onset Tay-Sachs disease mouse model.

## Keywords

autophagy,Tay-Sachs disease,mouse model

## Acknowledgement

This research was funded by TUBITAK 215Z083.

# Abstract: Seed priming effect on biochemical changes in model plant *Brachypodium distachyon* under abiotic stress

**Authors:** Beyza KOCAOGLU, Emrah NIKEREL, Bahar SOGUTMAZ ÖZDEMIR

**Affiliations:** Yeditepe University, Istanbul, Turkey

## Background/aim

Abiotic stress has a negative impact on plant metabolism and crop production. Seed priming is one of the most effective seed enhancement techniques that regulate the phases of germi- nation by controlled hydration. Seed priming improves the germination by treating seeds with vari- ous agents. One possible mechanism for this is the cell cycle arrest in G2 phase, owing to priming agents with high osmotic potential. This allows seeds to activate their antioxidant defense mechanism and prepares the plant for future stress conditions. The objective of this study is to investigate the biochemical responses related to seed priming in *Brachypodium distachyon* under salt and drought stress.

## Materials and methods

Seed priming was performed in *B. distachyon* (Bd-21 line) by three priming methods as hydropriming with distilled water, osmopriming with KNO3 and hormonal priming with salicylic acid (SA) in varying concentrations. *B. distachyon* plants were exposed to salinity by irrigation with 320 mM NaCl solution and drought stress by water withholding for 12 days. Relative water content (RWC) and relative membrane permeability (RMP) were performed to observe the effect of seed priming on plant physiology. The activities of antioxidants such as catalase (CAT), peroxidase (POD) and superoxide dismutase (SOD), and proline concentration for seed primed plants were determined under salinity and drought stress.

## Results

Physiological analysis revealed that highest damage occurred in 3% KNO_3_ and all SA primed plants under abiotic stress. The highest proline concentration was measured in 3% KNO_3_ and 0.5 mM SA primed plants. All priming methods increased POD activity while decreased SOD activity. However, CAT activity varied among priming methods.

## Conclusion

This study underlies the positive effect of seed priming (hydropriming at 20 C; osmopriming with 1% KNO_3_ and 2% KNO_3_) on abiotic stress tolerance of *B. distachyon*.

## Keywords

Seed priming,*Brachypodium distachyon*,abiotic stress,proline,antioxidant activity

# Abstract: Melanin concentrating hormone neurons control reward capacity independent of post-ingestive action

**Authors:** Pelin DILSIZ, Iltan AKLAN, Nilüfer SAYAR ATASOY, Yavuz YAVUZ, Gizem FILIZ, Fulya KÖKSALAR, Tayfun ATES, Merve ÖNCÜL, Ilknur ÇOBAN, Edanur ATES ÖZ, Utku CEBECIOGLU, Muhammed Ikbal ALP, Bayram YILMAZ, Deniz ATASOY

**Affiliations:** 1 Istanbul Medipol University, Istanbul, Turkey; 2 Yeditepe University, Istanbul, Turkey; 3 University of Iowa, Iowa City, Iowa, USA

## Background/aim

Evolutionarily, our brains drive great enthusiasm towards palatable nutrients. Craving and consuming palatable foods with high fat or sugar content often overcome homeostatic feeding, which in turn trigger obesity and binge-eating associated diseases with high mortality risk. Physiological hunger and satiety states are regulated by certain brain regions including lateral hypothalamic area where melanin concentrating hormone (MCH) neurons reside. However, neural circuits that control reward seeking by predominating physiological needs remain unidentified.

## Materials and methods

In our study, we used acute optogenetic and chemogenetic manipulation tools for functional characterization of MCH neurons. *Pmch-cre* transgenic mice were transduced with ChR2-eYFP, hM3D(Gq)-mCherry or hM4D(Gi)-mCherry bearing rAAV and probed for acute food intake, glucose sensitivity, locomotor activity and anxiety level. We also investigated rewarding capacity of MCH neurons performing optogenetic manipulation on two different operant tasks.

## Results

Our results indicate that MCH neurons were not involved in short-term appetite control unless stimulation was temporally paired with consummatory period. Inhibition of these neurons ameliorated glucose tolerance and was mildly anxiolytic. Yet, short-term locomotor activity did not alter following acute manipulation. Strikingly, we showed that activation of MCH neurons alone is sufficient to induce reward. Mice gained the reward value solely by having their MCH neurons activated, which in contrast could be obtained by consuming palatable nutrients. MCH neuron activation overrode the pleasure of binge-eating.

## Conclusion

Collectively, our findings address diverse behavioral and physiological functions of MCH neurons and suggest a direct role in reward function independent of post-ingestive actions. MCH neurons elevate the rewarding capacity regardless of chow food consumption rather than fulfilling a short-term appetite control.

## Keywords

Melanin concentrating hormone,reward,optogenetics,chemogenetics

# Abstract: The role RORalpha target genes in postmortem advanced atherosclerotic plaques and patients with CAD

**Authors:** Neslihan COBAN, Aybike Sena OZUYNUK, Aycan Fahri ERKAN, Mehmet CAVLAK, Nihan ERGINELUNALTUNA

**Affiliations:** 1 Istanbul University, Istanbul, Turkey; 2 Ufuk University, Ankara, Turkey; 3 Hacettepe University Faculty of Medicine, Turkey

## Background/aim

ROR-alpha, a transcription factor in the nuclear receptor family, has been found to play a role in the regulation of many biologic processes such as regulation of circadian rhythm, cho- lesterol metabolism, and suppression of inflammatory response. ROR-alpha shows ligand-dependent activity and melatonin and cholesterol are two of the known ligands which also important for athero- sclerosis development. In this study, the aim was to determine the expression levels of RORalpha and its target genes (*ABCA1, CYP19A1*, and *MIF*) in postmortem advanced atherosclerotic plaques. In ad- dition, a polymorphism of *CYP19A1* target gene has been investigated and associated with coronary artery disease (CAD).

## Materials and methods

The expression levels of RORalpha, *ABCA1, CYP19A1* and *MIF* genes in artery samples of postmortem cases with coronary heart disease (CHD) with advanced atherosclerotic plaques (CHDP) and without plaque (CHDnonP) were analyzed using qRTPCR. In addition, DNA samples of 332 Turkish CAD and non-CAD individuals were obtained using the inorganic method from leukocytes and individuals were genotyped for *CYP19A1* polymorphism using hybridization probes with LightCycler480 instrument. The angiographic severity was assessed by using Gensini and SYNTAX scoring systems.

## Results

In this study, it was found that ABCA1 and *MIF gene expressions* were upregulated while *CYP19A1* expression was downregulated in CHD-P. RORalpha expression was found to be slightly downregulated in CHDP when compared to CHDnonP (Fold change, 0.9). In addition, *CYP19A1* rs10046 polymorphism was found significantly associated with the angiographic severity in patients with CAD (p < 0.05).

## Conclusion

In this study, it was concluded that decreased expression of *CYP19A1* may play an ef- fective role in the development of atheroma. The expression levels of RORalpha and its target genes were found to be associated with advanced atherosclerosis in CHD cases which causes sudden death.

## Keywords

Atherosclerosis,Polymorphism,ROR-alpha

## Acknowledgement

This study was supported by the Istanbul University Scientific Research Projects Unit.

# Abstract: Suppression of Hsp60 and Hsp70 increases the anti-tumor effects of resveratrol in human glioblastoma cells

**Authors:** Evren ÜNAY UÇAR, Aslihan SENGELEN, Elif MERTOGLU, Murat PEKMEZ, Nazli ARDA

**Affiliations:** Istanbul University, Istanbul, Turkey

## Background/aim

In glioma cells overexpressed Hsp60 and Hsp70 have been closely associated with tumor cell proliferation. Resveratrol (RSV), a natural compound, has antitumor effects on glio- mas, but there are no reports regarding its effect on Hsp60 and Hsp70 expression in gliomas. The aim of this study was to investigate the effects of Hsp60 and Hsp70 silencing on the anti-cancer effects of resveratrol in U87MG human glioblastoma cells.

## Materials and methods

To determine cytotoxic effects of resveratrol and siRNAs on U87 MG human glioblastoma cells, MTT test was used. Cell transfections with Hsp60- and Hsp70-specific siRNAs were performed at concentrations of 25 and 50 nM. Potential inhibitory effects of resveratrol (10 and 15 μM) alone and in combination with siRNAs on Hsp expression were evaluated by Western blotting. The apoptotic activity was also evaluated by Western blotting using Bax, Bcl-2, caspase-3, PARP antibodies. Statistical analyses were performed using one-way ANOVA and Tukey post-hoc- test.

## Results

Resveratrol treatments inhibited cell proliferation in a dose- and time-depen- dent manner. After siRNA transfection, Hsp60 and Hsp70 protein levels were decreased by 61% and 69%, respectively. Our results show that combined treatments significantly increased the silencing effect on Hsps. In addition, the blocking of Hsp60 and Hsp70 expression made U87MG cells more sensitive to apoptosis.

## Conclusion

Increased induction of apoptosis as a result of siRNA-mediated silencing of Hsp60 and Hsp70 prior to resveratrol treatment may represent a novel therapeutic strategy for glioma patients.

## Keywords

Resveratrol,heat shock protein (Hsp),apoptosis,glioma

## Acknowledgement

The authors declare no conflict of interest. This work was supported by the Istanbul University Research Foundation, Turkey (Project Number: FBA-2018-30938).

# Abstract: Characterization of BRI3 as a novel Wnt/β-catenin pathway target

**Authors:** Izzet AKIVA, Necla BIRGÜL IYISON

**Affiliations:** Bogaziçi University, Istanbul, Turkey

## Background/aim

The Wnt pathway is evolutionary conserved, that has important functions in inver- tebrate and vertebrate developmental stages such as anterioposterior patterning, differentiation and proliferation. The binding of the Wnt ligand activate the pathway and relocate the key transcriptional activator b-catenin into the nucleus where it activates T-cell factor/Lymphoid enhancer factor (Tcf/ Lef) family of transcription factors, that calibrate the expression of developmental and cell cycle-re- lated genes. This pathway is known as one of the most important pathways for cancer development and progression. A number of targets, that have been identified for this pathway, are known to have tumorigenic characters. We verified that BRI3 gene is one of the transcriptional target genes of Wnt/ beta catenin signaling pathway, but it was not known to be associated with cancer, up to now its fun- ction remained largely unknown. One of our main objective is to characterize BRI3 and its biological roles and involvement in cancer progression.

## Materials and methods

BRI3 Overexpression, Reporter gene Assay, Western Blot analysis, Q- and Real Time PCR, Proliferation and Migration Assay, Xenograft Assay in SCID mice, and RNA-Sequ- encing from tumor tissues.

## Results

Overexpression of Huh7 cells show higher proliferative and invasive capacity compared to control cells. Moreover xenograft analysis show that BRI3 gene is responsible for larger tumor sizes in SCID mice. To investigate the eventual interacting pathways, RNA-Seq analysis was carried out from BRI3 expressing tumors from SCID mice.

## Conclusion

BRI3 is found to result in increased tumor formation by overexpression cells. Increase in tumor formation by BRI3 overexpression may be related to Hippo/YAP and NFkB signaling pat- hways.

## Keywords

Wnt/β-catenin pathway,BRI3,Xenograft,RNA-Sequencing

# Abstract: Cancer associated fibroblasts alter tumor microenvironment via influencing macrophage polarization

**Authors:** Gurcan GUNAYDIN

**Affiliations:** Hacettepe University, Ankara, Turkey

## Background/aim

Fibroblast cells differentiate to cancer/tumor associated fibroblasts (CAFs) in cancer tissue. Monocytes polarize to either M1 or M2-type macrophages. Identification of mecha- nisms affecting macrophage plasticity in tumor microenvironment may deliver crucial information concerning diagnostic, therapeutic strategies against cancer. Thus, this study aims to ascertain the effects of CAFs on macrophage differentiation in breast cancer.

## Materials and methods

Collagenase/hyaluronidase was used to isolate normal fibroblasts (NFs) and CAFs; from patients undergoing reduction mammoplasty, total mastectomy. Immunocytochemis- try was performed on CAFs/NFs to determine expressions of several markers (eg. α-Smooth-Muscle- Actin (αSMA), vimentin); to discriminate CAFs/NFs. Conditioned-mediums (CMs) were obtained from CAFs/NFs. Magnetic-bead based selection-protocols were utilized to isolate CD4^+^ T-cells and CD14^+^ monocytes. Flow-cytometric investigations were performed for CD206, CD163. CFSE assay was utilized to determine functional effects of CAF/NF-educated monocytes on CD4^+^ T-lymphocytes (activated by CD3/CD28 beads). Transwell-chambers were used to investigate the effects of CAFs/ NFs on monocyte-recruitment. Western-blot was utilized to examine E-cadherin, vimentin expres- sions. Transwell-inserts were used to analyze the invasion of MDA-MB-231 breast cancer cells.

## Results

CAFs express α-SMA unlike NFs. CAFs effectively recruit monocytes. Monocyte- chemotactic-protein-1 (MCP-1) or stromal-cell-derived-factor-1 (SDF-1) cytokines might be responsible from this recruitment, because inhibition of MCP-1/SDF-1 activity significantly reduced monocyte migration. Expressions of CD163, CD206 (M2-macrophage markers) by CAF-educated cells' were higher than by NF-educated cells. CAF-educated-monocytes suppress T-cell responses. CAF-educated-monocytes augmented invasive capacity of breast cancer cells; and increased vimentin expression, while decreasing E-cadherin expression in breast-cancer cells. CAFs polarized M1-macrophages to M2-like-macrophages; since CD163 expression significantly increased in M1- macrophages due to CAFs.

## Conclusion

CAFs from breast cancer patient tissues polarized monocytes into M2-like pro-tumoral macrophages phenotypically, functionally, ex vivo, unlike NFs. CAFs were very potent in recruiting monocytes. MCP-1 and SDF-1 may prove to be crucial monocyte chemotactic cytokines which are secreted from stromal cells.

## Keywords

CAF,macrophages,tumor microenvironment

# Abstract: Anti-inflammatory effect of *Momordica charantia* on TNBS-induced rat colitis

**Authors:** Asli SEMIZ

**Affiliations:** Pamukkale University, Denizli, Turkey

## Background/aim

Ulcerative colitis (UC) is a chronic disease that diffuses from the rectum to the proximal length without leaving any intact parts and diffuses the colon mucosa with remission and exacerbations. In Turkish folk medicine, *Momordica charantia* (MC) has been frequently used for curing of cutaneous lesions, peptic ulcer and colitis. This study was aimed to examine the anti- inflammatory effect of MC in trinitrobenzene sulfonic acid (TNBS)-induced rat coliti

## Materials and methods

UC was induced in rats by intra-rectal catheterization of 30 mg/0.1 ml TNBS dissolved in 0.5 ml of 50% ethanol. Standard drug prednisolone (PR, 2 mg/kg/day, 7d) was given on the same day by oral route. After 7 d, rats with colitis were treated with MC (4 g/kg/day) for 14 consecutive days by intragastric gavage. The mRNA levels for IL-1β, IL-6, IFN-γ and TNF-α were measured to estimate the effect of MC in colonic tissue.

## Results

MC and prednisolone reduced the body weight loss. Additionally, MC and PR administrations decreased the levels of IL-1β, IL-6, IFN-γ and TNF-α in colon tissue.

## Conclusion

These results indicated that MC showed anti-inflammatory effect in rat colitis and indicated MC may be a therapeutic candidate for the treatment of UC. However, it should be kept in mind that individual dose adjustment is essential. In addition, studies, such as Western blot and histological analysis, are still underway to determine the exact mechanism of MC as an anti- inflammatory agent in colitis.

## Keywords

*Momordica charantia*,ulcerative colitis,rat model,inflammatory cytokines,trinitrobenzene sulfonic acid

## Acknowledgement

This work was supported by a grant from The Scientific and Technological Research Council of Turkey (216Z093).

# Abstract: The effectiveness of pterostilbene on the ototoxicity induced by diabetes mellitus

**Authors:** Sibel ÖZDAS, Bora TASTEKIN, Seren G. GÜRGEN, Talih ÖZDAS, Aykut PELIT, Sanem O. ERKAN, Birgül TUHANIOGLU, Birgül GÜLNAR, Orhan GÖRGÜLÜ

**Affiliations:** 1 Adana Alpaslan Türkes Science and Technology University, Adana, Turkey; 2 Çukurova University, Turkey; 3 Celal Bayar University, Manisa, Turkey; 4 Health Science University Adana City Hospital, Adana, Turkey

## Background/aim

Diabetes mellitus (DM) causes ototoxicity, and various histological changes and hearing loss in the cochlea. The purpose of this study was to examine the anti-ototoxic impact of Pterostilbene (PTS) on the model of *streptozotocin* (STZ)-induced DM in rats.

## Materials and methods

Thirty Wistar albino male rats were examined with the Distortion product otoacoustic emission (DPOAE) tests, and randomly divided into five groups: Control (healthy control), DM (diabetic control) DM+PTS10, DM+PTS20 and DM+PTS40 (as diabetic rats treated with intraperitoneal PTS at 10, 20, or 40 mg/kg/day, respectively). DPOAE test was repeated on four- week experimental period on all the groups. Finally, immunohistochemistry, histopathological and biochemical examination was performed. Quantitative real-time polymerase chain reaction was used to assess the mRNA expression levels of the *Casp-3, Bax*, and *Bcl-2*.

## Results

Adose-dependent increase of the mean DPOAE amplitudes was observed with PTS treatment (P < 0.05 for all). The hair cells of the Corti organ of the DM+PTS treatment groups were observed to have weak the caspase-8, cytochrome-c, and apoptotic-positive cell numbers expression but strong reactions were determined in the DM group (P < 0.05 for all). Also, PTS treatment groups compared to DM, statistically significant an increase in the mRNA expression of the *Bcl-2* but a decrease the Bax and Casp-3 in a dose-dependent manner were observed (P < 0.05 for all). PTS treatment significantly improved the metabolic parameters of the diabetic rats, such as body weight, blood glucose, serum insulin, and MDA levels, consistent with our other findings (P < 0.05 compared to DM for all).

## Conclusion

This study reports the first in vivo findings to suggest that there is a protective effect of PTS on DM-dependent ototoxic rat model

## Keywords

Diabetes mellitus,Pterostylbene,Ototoxicity

# Abstract: Anticarcinogenic effect a novel alkaloid: 1H-indole-2-hydroxy, 3 carboxylic acid

**Authors:** Ozden OZGUN ACAR, Elif KALE, Hatice ORUC, Isil GAZIOGLU, Gulacti TOPCU, Alaattin SEN

**Affiliations:** 1 Pamukkale University, Denizli; 2 Bezmialem Vakif University, Istanbul; 3 Abdullah Gül University, Kayseri

## Background/aim

Cancer is a problem with worldwide importance and is the second most common cause of mortality globally. Phytochemicals isolated from herbs are always the biggest agents in the study of new anti-cancer agents. It was aimed to evaluate the in vitro effects of 1H-indole-2-hydroxy, 3 carboxylic acid (IHCA) isolated from *Capparis ovata* on diverse molecular targets to give proof to its promising potential anti-cancer action for further research.

## Materials and methods

LNCaP and HEK293 cell lines were purchased American Tissue Culture Collection. The cells were cultured in DMEM medium containing 10% FBS, 0.5% penicillin/strep- tomycin at 37 C in a humidified atmosphere comprised of 95% air and 5% CO2. The crystal violet staining assessed the cell viabilityin the conditioned media. The protein and mRNA levels of P53 and ACTB were determined by western blotting and qPCR, respectively. HEK293 cells were transfected with the pGL4.38[luc2P/p53 RE/Hygro] Vector. After 24 hours of transfection, cells were stimulated with IHCAand Doxorubicin (as positive control). Luminescence was detected using the Dual-Glo Luciferase assay system.

## Results

IHCA did not significantly elicit the P53 gene expression in EC25 dose while the increase in the level of P53 protein and mRNA were observed in the cells treated with EC50 dose of IHCA. HEK-293 cells transfected with P53 firefly luciferase (Luc) were treated with doxorubicin and IHCA for the time indicated,and luciferase activities were measured. It was found that doxorubicin (1.2- fold) and IHCA(1.7-fold) significantly increased luciferase activity compared to control.

## Conclusion

These data provide the molecular elucidation for the anti-cancer properties of IHCA and propose that this alkoloid may be a potential agent in treating prostate cancer.

## Keywords

Alkaloid,1H-indole-2-hydroxy,3 carboxylic acid,Anti-cancer

## Acknowledgement

This work is supported by TÜBITAK (The Scientific and Technological Re- search Council of Turkey) 112S187 and Pamukkale University PAUBAP 2017FEBE051.

# Abstract: Possible relationship between mevalonate kinase and cell migration from perspective of mevalonate kinase deficiency

**Authors:** Tayfun Hilmi AKBABA, Z.Yeliz AKKAYA-ULUM, Selcan DEMIR, Seza ÖZEN, Banu PEYNIRCIOGLU

**Affiliations:** Hacettepe University, Ankara, Turkey

## Background/aim

Mevalonate Kinase Deficiency(HIDS/MKD, MIM#260920) isanautoinflammatory syndrome resulted from mutations in MVK gene.Recurrent attacks are seen in patients with abdominal pain, adenopathy in lymph nodes and increased level of immunoglobulin D(IgD).In this study, we aimed to analyze cell migration which is an important process in inflammation by using primary cells of MKD patients.

## Materials and methods

Monocytes were obtained from 2 MKD, 11 FMF patients(as disease control) and 7 healthy controls. We incubated cell with 1 ug/ml LPS to activate inflammation. Transwell experiments were performed in activated cell. Calcein-AM was used as living cell marker and migrating cells counted with ImageJ programme.

## Results

MKD patients'cells did not migrate under both conditions.Number of migrated cells elevated in FMF patients compared to controls. The possible effect of anti-IL-1 treatment, the drug used for MKD patients, on cell migration was eliminated due to the observation of increased cell migration in minor FMF patients having the same treatment.

## Conclusion

Loss of function in mutant mevolanate kinase enzyme leads to a reduction in the amount of geranylgeranyl pyrophosphate required for protein prenylation.The regulation of many biological processes by protein prenylation and the prenylation of actin-associated proteins(Rho, Rac1, Rab, etc.), which play an important role in cell migration, reinforces the hypothesis that mevalonate kinase may be effective in this process. This study is the first study to demonstrate the possible relationship between mevalonate kinase and cell migration from the MKD perspective.

## Keywords

Mevalonate Kinase Deficiency,cell migration,prenylation

## Acknowledgement

This project was supported by Hacettepe University Scientific Research Coordination Unit(Project Number: TYL-2018-17354).

# Abstract: Silencing of HSF1 increases the apoptosis induction in human glioblastoma cells upon rosmarinic acid treatment

**Authors:** Aslihan SENGELEN, Evren ÜNAY UÇAR

**Affiliations:** Istanbul University, Istanbul, Turkey

## Background/aim

Glioblastomas (GBM) are the most malignant primary brain tumors. Elevated lev- els of heat shock proteins (Hsp) and their transcription factor heat shock factor 1 (HSF1) in gliomas are effective in tumor cell-proliferation. Rosmarinic acid (RA), a natural compound, is known to exert anticancer effects for gliomas. However, there is no evidence about its effect on HSF1 expression. Herein, we aimed to investigate the effect of RA on HSF1 levels and apoptosis induction in GBM cells.

## Materials and methods

HSF1 was silenced using small interfering (si)RNA. MTT assay was used to examine the cytotoxic effects of RA and siRNAs. RA (80 and 215 μM) was administered alone or in combination with siRNAs (25 and 50 nM) to U87MG human GBM cells. The effects of the treat- ments on the levels of HSF1, several Hsps and apoptosis-related proteins were evaluated by Western blot analysis. Differences among groups were tested by oneway ANOVA followed by Tukey post- hoc-test.

## Results

We found that RA blocks cell proliferation in a dose- and time-dependent manner. HSF1 siRNA administration alone caused a 70% reduction in HSF1 levels, while combined treatment with RA resulted in almost depletion of the protein. All treatments caused the downregulation of the tested Hsp expression levels and triggered apoptosis. Moreover, our results showed that HSF1 inhibition makes U87MG cells extremely sensitive to apoptosis before RA treatment.

## Conclusion

Taken together our findings suggest that combined treatment (RA + HSF1-siRNA ad- ministration) has the potential to be used as a candidate for GBM treatment.

## Keywords

Rosmarinic acid (RA),heat shock factor 1 (HSF1),heat shock protein (Hsp),apoptosis,glioblastoma (GBM)

## Acknowledgement

This work was supported by the Istanbul University Research Foundation, Turkey (Project Number: FDK-2019-32757).
